# Pneumococcal population structure influences the effects of air pollution on invasive disease risk in South Africa

**DOI:** 10.1038/s41564-026-02458-5

**Published:** 2026-09-14

**Authors:** Sophie Belman, Cebile Lekhuleni, Jackie Kleynhans, Giovenale Moirano, Daniela Lührsen, Cristina Carnerero, Susan Meiring, Stephanie W. Lo, Mignon du Plessis, Anne von Gottberg, Rachel Lowe

**Affiliations:** 1 Barcelona Supercomputing Center (BSC), Barcelona, Spain; 2 Parasites and Microbes, Wellcome Sanger Institute, Hinxton, UK; 3 Centre for Respiratory Diseases and Meningitis, National Institute for Communicable Diseases of the National Health Laboratory Service, Johannesburg, South Africa; 4 Department of Clinical Microbiology and Infectious Diseases, School of Pathology, Faculty of Health Sciences, University of the Witwatersrand, Johannesburg, South Africa; 5 School of Public Health, Faculty of Health Sciences, University of the Witwatersrand, Johannesburg, South Africa; 6 Department of Medical Sciences, University of Turin, Torino, Italy; 7 Division of Public Health Surveillance and Response, National Institute for Communicable Diseases of the National Health Laboratory Service, Johannesburg, South Africa; 8 European Molecular Biology Laboratory–European Bioinformatics Institute, Cambridge, UK; 9 Division of Medical Microbiology, Department of Pathology, Faculty of Health Sciences, University of Cape Town, Cape Town, South Africa; 10 Catalan Institution for Research and Advanced Studies (ICREA), Barcelona, Spain; 11 Centre on Climate Change and Planetary Health and Centre for Mathematical Modelling of Infectious Diseases, London School of Hygiene and Tropical Medicine, London, UK; 12 Present Address: Environmental Health Sciences, Yale School of Public Health, New Haven, CT USA

**Keywords:** Climate-change ecology, Bacterial infection, Bacterial genes, Risk factors

## Abstract

*Streptococcus pneumoniae* is highly diverse, comprising over 100 serotypes and hundreds of genomic lineages amid widespread vaccination. While it can cause invasive pneumococcal disease (IPD) which exhibits pronounced seasonal spikes, the interplay between pneumococcal diversity and environmental drivers remains unexplored. Here we analysed 59,017 IPD cases over 19 years from South Africa, incorporating 4,350 genome-sequenced isolates, using Bayesian spatiotemporal models to link environmental exposure and pneumococcal diversity. Cumulatively, across an 8-week period, moderate relative humidity (33–49%) and cold minimum temperatures (4–10 °C) increased IPD risk by 5% and 4%, respectively. Conversely, warm maximum temperatures (27–38 °C) were associated with up to a 10% increased risk within a week of exposure. There was a positive association between air pollution (PM_2.5_) and IPD, although it varied by age, disease presentation, and most notably serotype and lineage. Specifically, the lag time between PM_2.5_ exposure and disease onset varied by serotype, with only serotypes 4, 8 and 23F conferring an immediate IPD risk. High prevalence of GPSC21 lineage (serotype 19F) also modified the pollution response, shifting the lag structure to produce immediate risk of disease following high PM_2.5_ exposure. Our results demonstrate that pneumococcal population structure shapes air quality risk which in turn can shape the fitness landscape of microbial populations. Integration of these data may inform public health policy.

## Abstract (web summary)

Air quality and climate factors alter the risk of invasive pneumococcal diseases such as meningitis. Importantly, the lineage and serotype (population structure) of circulating bacteria can alter how quickly disease risk rises in the weeks following exposure.

## Main

*Streptococcus pneumoniae* (pneumococcus) is asymptomatically carried in the human nasopharynx but can progress to non-invasive disease (for example, otitis media) or invasive pneumococcal disease (IPD) (for example, bacteraemia or meningitis) when it breaches the epithelium^1^. Pneumococci are highly diverse, comprising hundreds of genomic lineages (Global Pneumococcal Sequence Clusters; GPSCs)^2^ and over 100 serotypes^3^. Progression through asymptomatic carriage to invasive disease is mediated by a combination of environmental drivers, microbial virulence and host immunity. There is known variability in virulence, carriage durations and sociodemographic populations affected by different lineages and serotypes^2,4^. Despite high vaccination coverage, IPD shows consistent seasonal spikes each year^5^. Some serotypes are targeted by vaccines such as the pneumococcal conjugate vaccines (PCV) and despite overall IPD reductions, the years following vaccine implementation have seen replacement by non-vaccine-targeted serotypes (NVTs) globally^4,6–8^. Efforts to reduce IPD burden focuses on adapting vaccination strategies to varied sociodemographic contexts and pneumococcal colonization densities^9,10^.

However, the role of air quality and meteorological factors, such as air pollution, temperature and humidity, in pneumococcal colonization and disease dynamics are typically overlooked. Many geographic regions with the highest pneumococcal disease burden also have populations with extensive exposure to polluted air^11^. Existing evidence around the impact of environmental factors on pneumococcal disease is heterogenous^12^. Impacts of temperature and humidity on pneumococcal disease vary by climate, with inconsistent global patterns^13–16^. Similarly, air pollution is widely considered to increase IPD risk but the mechanisms are poorly understood^12,17^. Most studies have relied on correlations, few assessed delayed effects (lag time from exposure to outcome), or controlled for confounding meteorological effects^16,18,19^.

Important gaps remain in understanding nonlinear and lagged effects, and how risks vary by age, disease type, serotype and lineage. Accounting for pneumococcal diversity together with environmental factors can uncover the biological mechanisms behind environment–disease interactions ultimately towards improved vaccination strategy modelling and hospital capacity preparations with changing climates.

Here we investigate the relationship between a range of air quality and meteorological factors on IPD using case data from over 500 hospitals across 19 years in South Africa. This is a setting with pneumococcal colonization estimates ranging from ~40–60%^20,21^, and marked geographic and climatic diversity. Major public health interventions occurred during the study period (PCV7 introduction in 2009, PCV13 in 2011, and widespread antiretroviral therapy (ART) for treatment of people living with HIV (PLHIV), since 2005) (Supplementary Table 1). We focus on a major component of air pollution, particulate matter <2.5 μm in size (PM_2.5_) and the associated IPD risk, while accounting for pneumococcal phenotypic and genomic data, to understand interactions with underlying microbial ecology across different serotypes and lineages. We apply Bayesian hierarchical models to capture spatial and temporal variability, incorporate delayed and nonlinear relationships between environmental exposure and disease onset, and allow for stratification by age group and disease type. These models enable a better understanding of the interaction between environmental factors and pneumococcal populations, and how it plays a role in IPD dynamics.

## Results

**Fig. 1:**
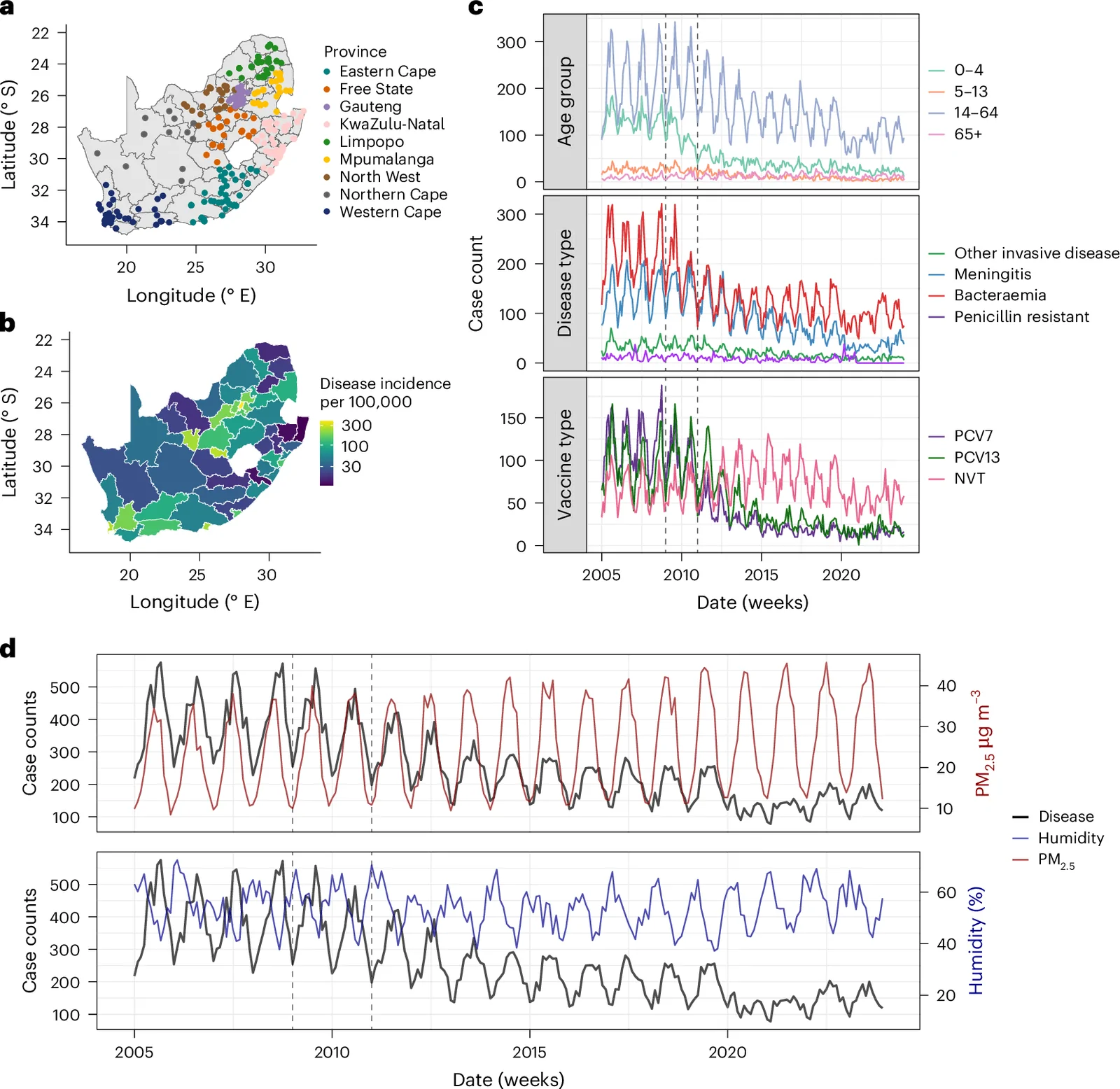
Summary of epidemiological and environmental datasets in South Africa. **a**, Hospital locations (*n* = 531) coloured by province (*n* = 9). **b**, log10 incidence rate per 100,000 from 2005 to 2023 by district (*n* = 52) in South Africa. **c**, Monthly case counts by age group, disease type (bacteraemia, meningitis, other IPD types, penicillin-resistant) and vaccine type (PCV7, 7-valent vaccine type serotypes; PCV13, additional 6 serotypes in the 13-valent vaccine type; NVT, non-vaccine types). **d**, Monthly pneumococcal disease case counts (black) with mean monthly particulate matter <2.5 μm concentration (PM_2.5_, μg m^−3^) (red) and mean relative humidity (blue) across South Africa from 2005 to 2023; left axis is the monthly case counts, right axis is the concentration (μg m^−3^) of particulate matter <2.5 μm (top) and percent relative humidity (bottom). Vertical dashed lines in **c** and **d** indicate years of PCV7 (2009) and PCV13 (2011) implementation. Source data

**Extended Data Fig. 1:**
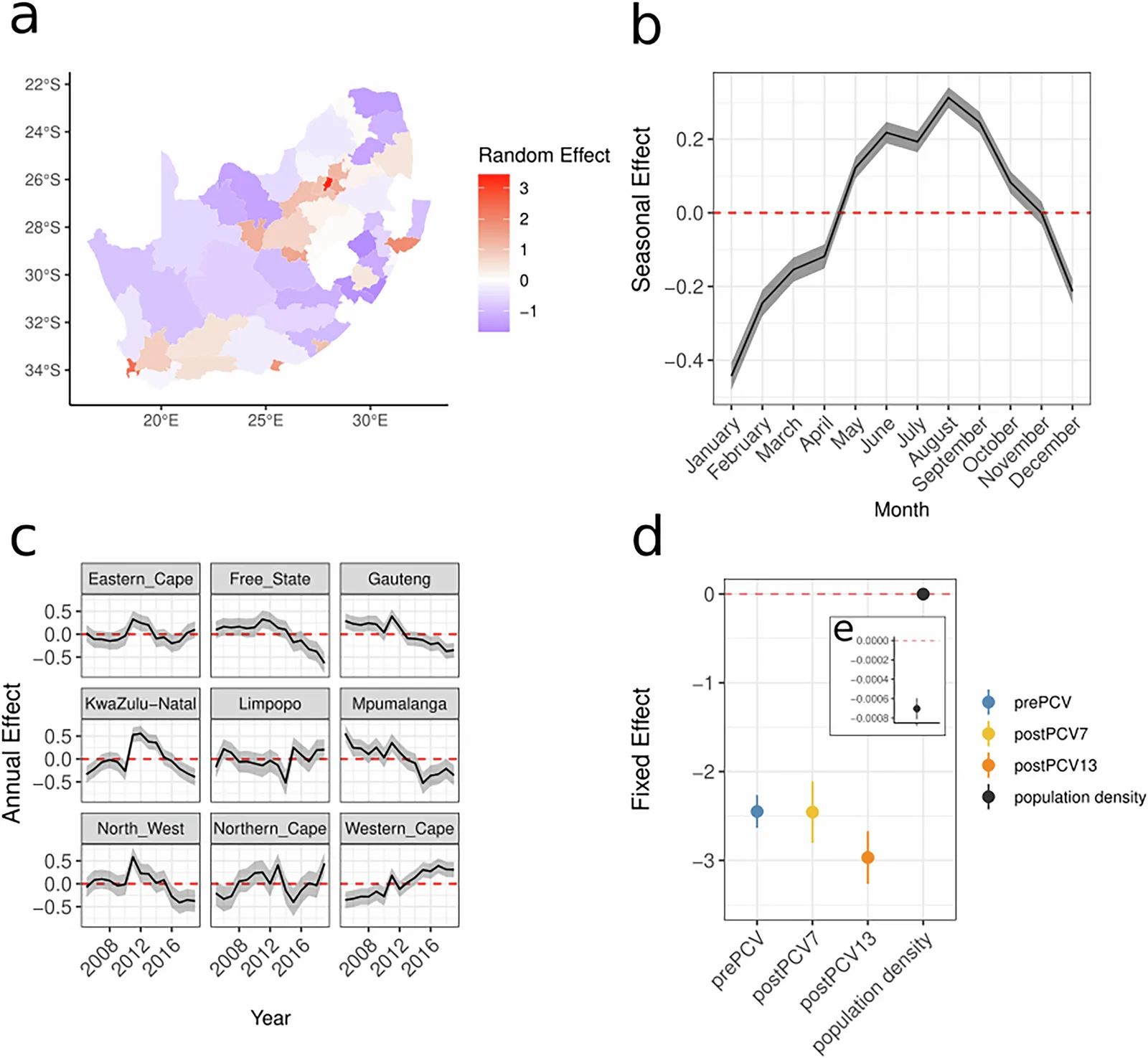
Spatial, seasonal, and interannual effects from the sociodemographic base model. (**a**) Median spatial random effects across 52 districts where a positive effect (more cases than expected on average) is red and a negative effect (fewer cases than expected on average) is blue, (**b**) median monthly random effects across the 12 months of the year, the red line denotes effect size of 0, (**c**) median yearly random effects 2005-2019 replicated across the 9 provinces of South Africa (**d**) median fixed effects of vaccination for each vaccine period pre-PCV7 (2005-2008) (blue), post-PCV7 (2009-2011) (yellow), and post-PCV13 (2012-2019) (orange), and median population density fixed effect (black) (**e**) the same population density fixed effect shown in d but with y-axis reduced (-8×10^−4^,0) to highlight the effect. The credible intervals for the spatial random effects in a are reported in Table S14. The shading and error bars for b-e represent 95% credible intervals. Case counts across the time period by vaccine type are included in Fig. 1. Source data

A total of 59,017 identified IPD cases were included from 531 hospitals in South Africa between 2005 and 2023. Most IPD cases were found in urban centres where the majority of the South African population resides, including 41% of total IPD from Gauteng province, 18% from Western Cape province, as well as 11 and 9% from KwaZulu-Natal and Eastern Cape provinces (Fig. 1a,b). Disease cases included bacteraemia, meningitis and other atypical IPD types such as pericarditis and empyema. The majority of cases occurred in people aged 15–64 years (62%), followed by children under 6 years (23%). Of cases, 4.8% were from >64-year-olds. There was a well-characterized decrease in serotypes targeted by PCVs (Supplementary Table 1). We observed NVT persistence (Fig. 1c) and consistent seasonality in IPD cases. Cases peaked in colder months across the time period coinciding with the seasonality of PM_2.5_ concentration and inversely correlating with relative humidity (Fig. 1d, Extended Data Fig. 1, and Supplementary Fig. 1 and Table 2).

### Associations between environmental factors and pneumococcal disease risk

**Extended Data Fig. 2:**
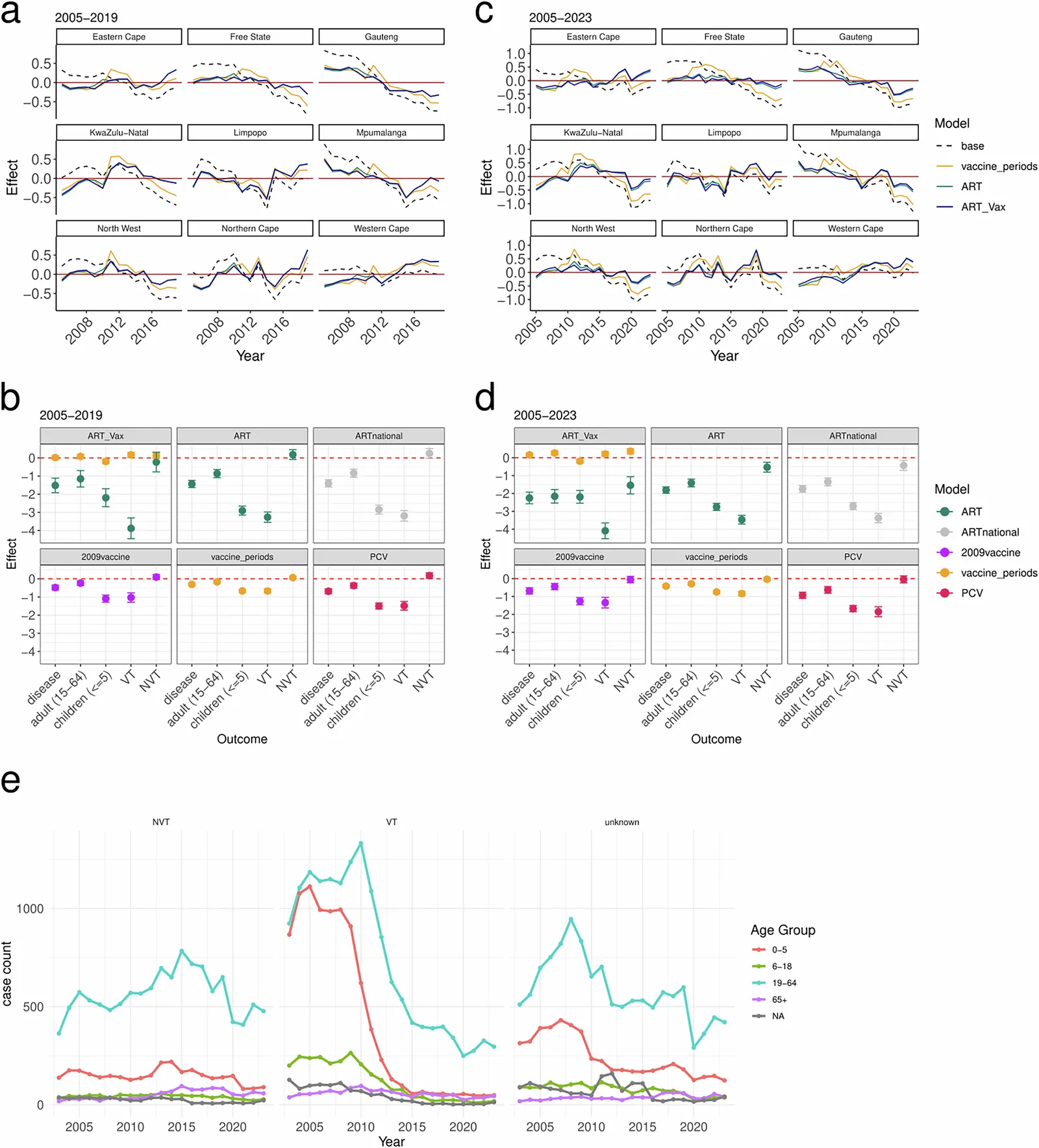
**Effects from implementation of ART and vaccine included in the models** **with years** (**a-b**) **2005-2019 and** (**c-d**) **2005-2023.** (**a**) and (**c**) show the interannual random effects with no perturbations (black, dashed), for a model with vaccination periods 2009 and 2011 (yellow), for ART coverage per province (green), and for a model including both vaccination periods and ART coverage per province (blue). (**b**) and (**d**) include a model with ART_Vax: both ART coverage per province (green) and vaccination periods (yellow), ART: a model with ART coverage per province (green), ARTnational: ART coverage nationally (grey), 2009vaccine: a model with a categorical variable indicating pre-PCV7 (2005-2008) and post-PCV (2009-2023) (purple), vaccine_periods: a categorical covariate indicating the pre-PCV, PCV7 (2009-2010), and PCV13 (2011-2023) periods (yellow), and a model with PCV coverage nationally (pink). These each were run for outcomes of all IPD, IPD in adults 15-64, children ≤5, VT serotypes, and NVT serotypes independently and error bars represent 95% credible intervals reported around the median, (**e**) shows the case count across years and by age group including 0-5 (pink), 6-18 (green), 18-64 (blue), 65+ (purple), and absent age data (NA) (grey) for disease from NVTs (left), VTs (middle), and unknown serotype (right). Source data

**Extended Data Fig. 3:**
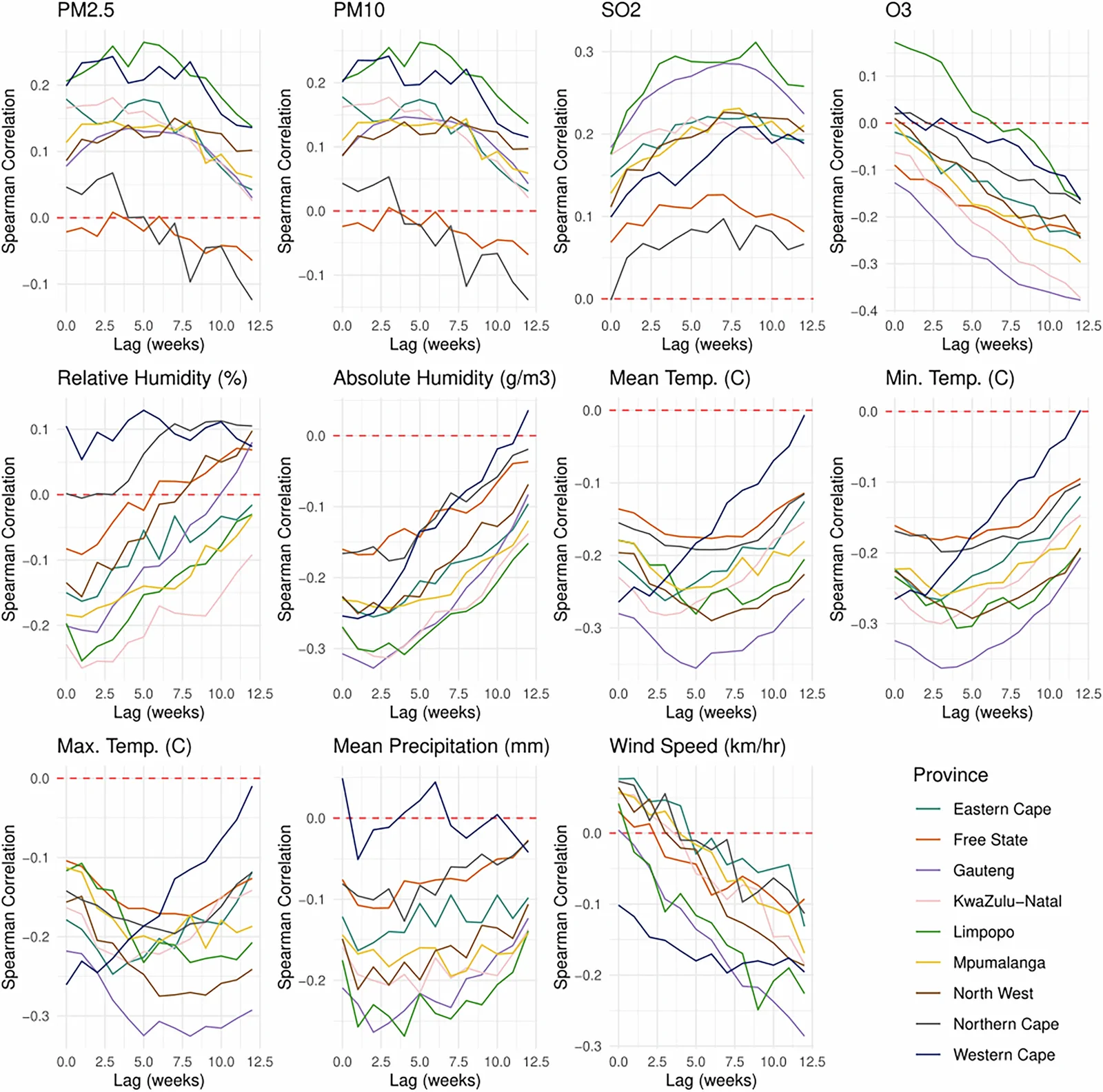
Spearman correlation between each lagged variable and invasive pneumococcal disease case counts. Across lag weeks with disease case counts for all environmental variables colored by province across 12 weeks of lag. Source data

**Extended Data Fig. 4:**
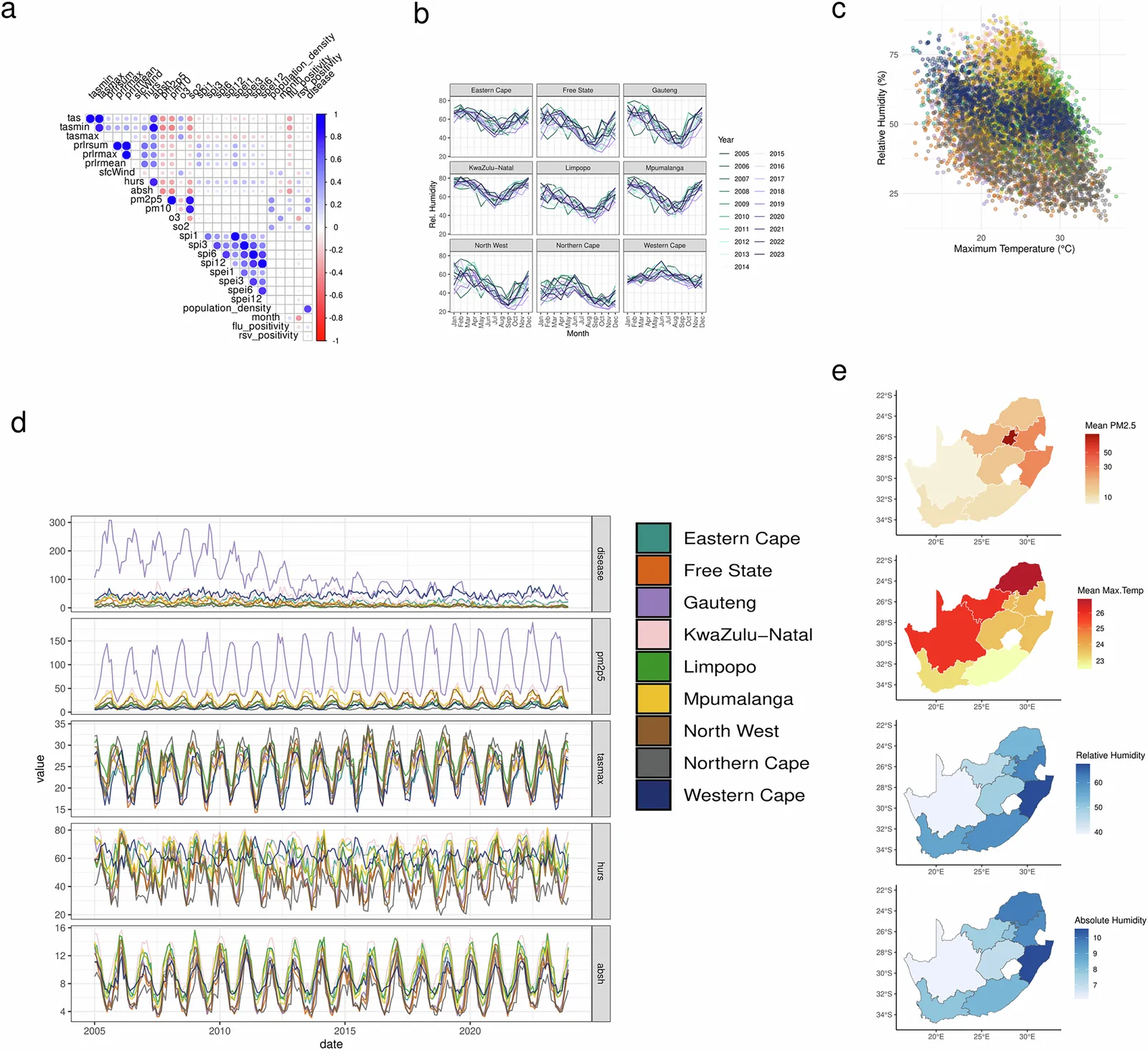
Summaries of included environmental variables. (**a**) Correlation plot with Pearson correlation between each of the external variables included. (**b**) relative humidity across months colored by the years of the study faceted by province. (**c**) Correlation between maximum temperature and relative humidity colored by province (same legend as d). (**d**) time series of pneumococcal disease case counts and (**e**) mean of each environmental variable per the 9 provinces of South Africa (**d-e**) PM_2.5_ concentration (μg/m^3^), maximum temperature (celsius), relative humidity (%), and absolute humidity (g/m^3^ water) across 19 years colored by province. Source data

We developed a sociodemographic base model to estimate IPD incidence, which included spatial autocorrelation across districts, seasonal effects and interannual random effects replicated across provinces, as well as pneumococcal vaccine implementation periods and population density (Extended Data Figs. 1 and 2). This model was extended to estimate IPD risk from environmental factors using distributed nonlinear models (DLNMs), which allowed us to account for delayed and nonlinear effects up to 8 weeks after environmental exposure (Supplementary Table 3, and Extended Data Figs. 3 and 4)^8,16,17,19^.

**Fig. 2:**
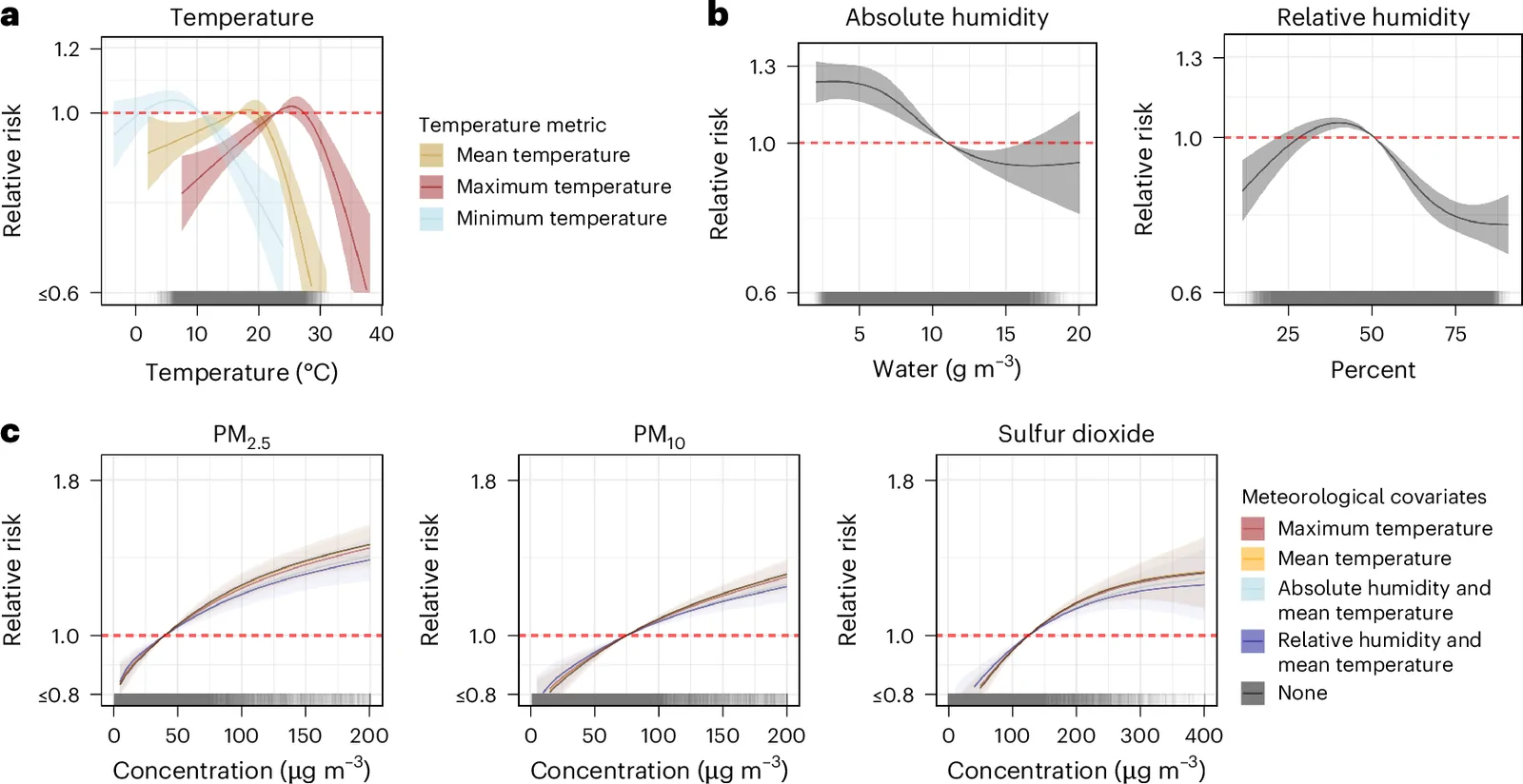
Cumulative relative risk across 8 weeks of lags for meteorological and air pollution models. **a**,**b**, Meteorological models. **a**, Exposure–response curves for maximum (pink), mean (yellow) and minimum (blue) temperature in °C. **b**, Exposure–response curves for absolute humidity (g m^−3^) (left) and % relative humidity (right). **c**, Air pollution models. Exposure–response curves for PM_2.5_ (left) PM_10_ (middle) or sulfur dioxide (SO_2_) (right) including models with combinations of meteorological covariates (temperature metrics with linear formulations and humidity metrics with nonlinear formulations): maximum temperature (red), mean temperature (yellow), absolute humidity and mean temperature (light blue), relative humidity and mean temperature (dark blue), and none (black) with 3-week lags for humidity and no lag for temperature. These models were fitted to weekly district-level data from 2005 to 2019 across South Africa. The DLNM cross-basis for the humidity variables has 3 degrees of freedom, while the rest of the DLNMs have 2 degrees of freedom. At the bottom of **a**–**c** are rug plots with the distribution of the variables in the DLNM. In **a**, the rug plot is for mean temperature. Shading represents 95% credible intervals and points indicate the median. Source data

**Extended Data Fig. 5:**
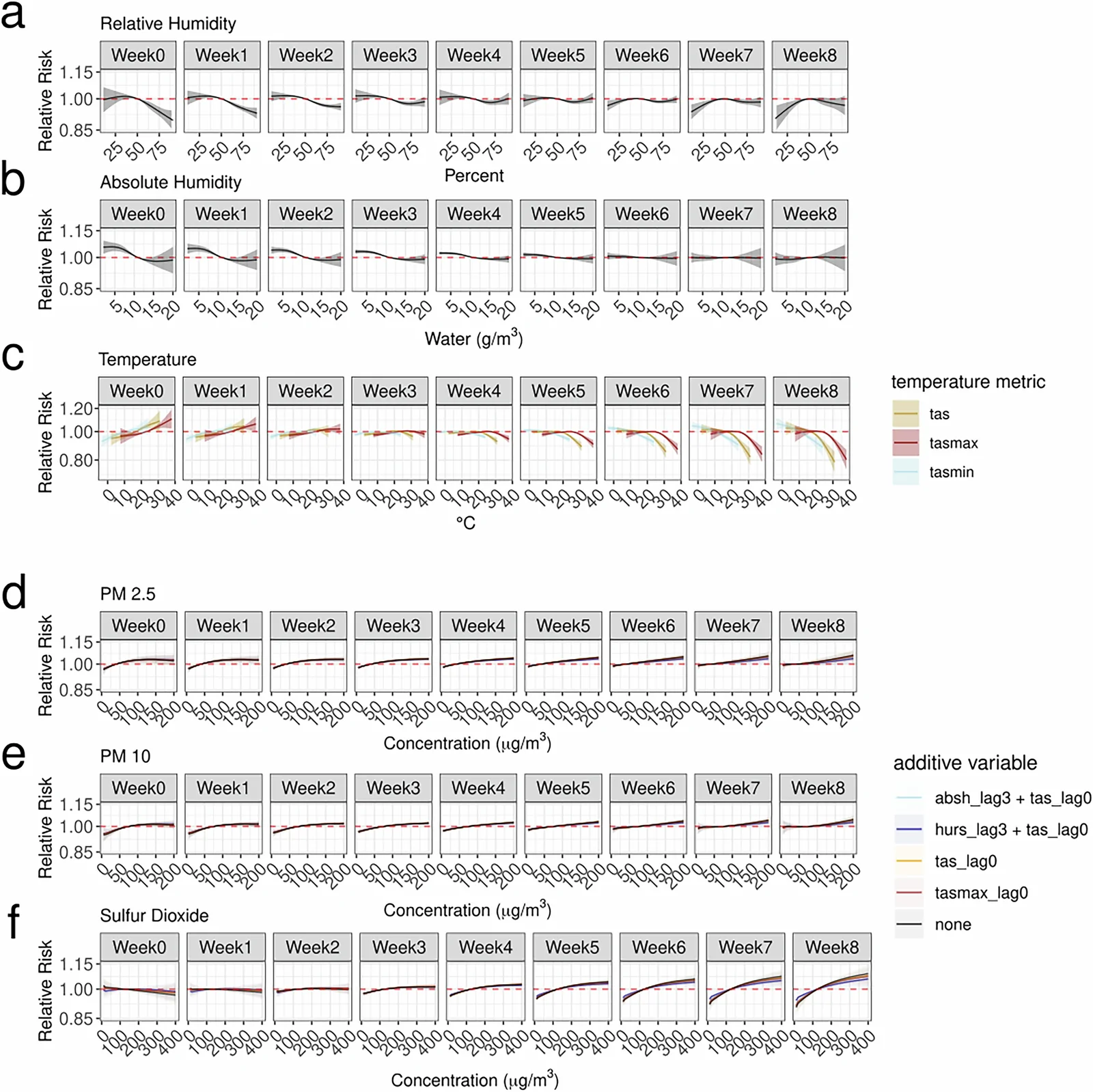
**Relative risk across 8-weeks of lags for relative, and absolute humidity, temperature, and air pollutants PM**_**2.5**_**, PM**_**10**_ **SO**_**2**_, with additional multiplicative variables which account for vaccination period and population density, as well as residual spatial, seasonal, and interannual random effects. a) Exposure response curves for (**a**) absolute humidity (g/m^3^) and (**b**) % relative humidity. (**c**) Exposure response curves for maximum (tasmax) (pink), mean (tas) (yellow), and minimum (tasmin) (blue) temperature in celsius (°C). Exposure response curves for (**d**) PM_2.5_, (**e**) PM_10_, or (**f**) sulfur dioxide including models with combinations of meteorological covariates: maximum temperature (tasmax_lag0) (red), mean temperature (tas_lag0) (yellow), absolute humidity and mean temperature (absh_lag3 + tas_lag0) (light blue), relative humidity and mean temperature (hurs_lag3 + tas_lag0) (dark blue), and none (black). These models are fit to weekly district level data from 2005-2019 across South Africa. In the DLNM the humidity variable cross basis has 3-degrees of freedom (df), while the pollution and temperature variables have 2. The additional multiplicative effects are included as fixed effects except the humidity variables which are non-linear with 5 cuts. The shaded error bars represent 95% credible intervals and the points indicate the median. Source data

Meteorological models showed a nonlinear cumulative risk for temperature (Fig. 2a), with underlying divergent lag structures: warm temperatures triggered acute risks, whereas cold-related risks were delayed. Only cold temperatures (4–10 °C) showed a significant cumulative increase (6 °C relative risk (RR): 1.04; 95% credible intervals (CI) 1.01–1.06), although in warmer weeks (31–38 °C) we found a 5–11% risk increase relative to the median (Extended Data Fig. 5c and Supplementary Fig. 2).

Relative humidity provided a better model fit (Δ Watanabe–Akaike information criterion (WAIC) 77.7) than absolute humidity (the absolute amount of vapour in air, g m^−3^). Absolute humidity had a stronger association at low values (4 g m^−3^, cumulative RR 1.23: 95% CI 1.16–1.30) with an acute increase in disease risk until a 2-week lag time. For relative humidity, the cumulative risk was nonlinear. The highest risk occurred at moderate humidity levels (34–47%) with a maximum cumulative RR of 1.05 (95% CI 1.03–1.07) at 39.8% relative to the median (Fig. 2b). However, at 74.8% relative humidity there was a strong protective effect (RR 0.77: 95% CI 0.73–0.88) (Fig. 2b). Weaker effects observed for low relative humidity probably reflect dependence on temperature (indicating that temperature mediates humidity-related effects) (Fig. 2b, Extended Data Fig. 5 and Supplementary Table 4).

**Extended Data Fig. 6:**
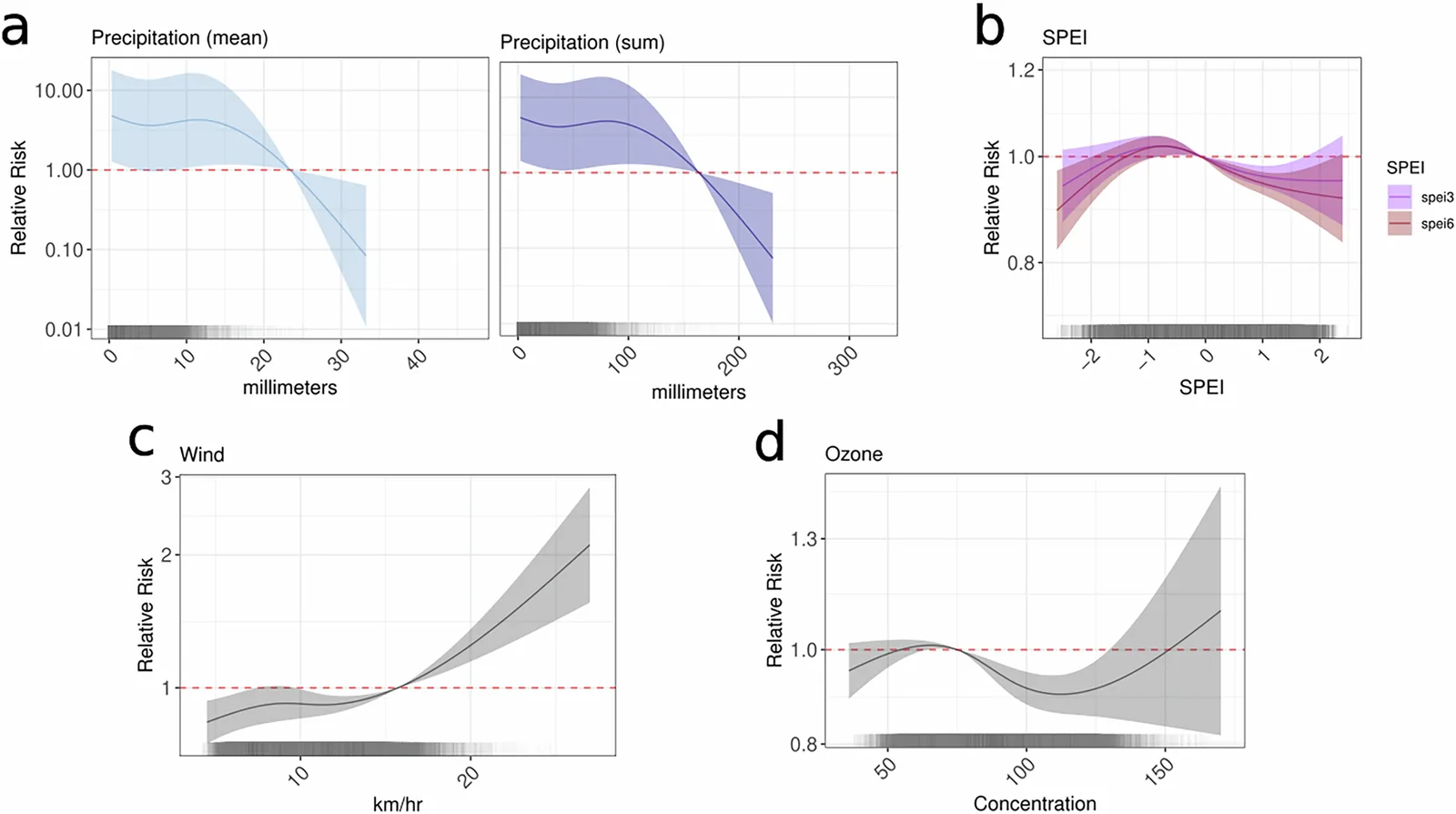
Cumulative 8-week relative risk with models fit to weekly district level IPD case data for precipitation, standard evapotranspiration index, and ozone. Cumulative 8-week relative risk with models fit to weekly district level IPD case data with the sociodemographic base model and meteorological DLNMs for (**a**) precipitation mean (left; light blue) precipitation sum (right; dark blue), and (**b**) standard precipitation evapotranspiration index (SPEI) 3 months (purple) and 6 months (brown) (**c**) wind kilometers per hour (km/hr) (**d**) ozone (μg/m^3^). The shaded error bars represent 95% credible intervals and the points indicate the median. Source data

Increasing precipitation and a higher standardized precipitation and evapotranspiration index (SPEI) (reduced drought conditions) decreased IPD risk (negative associations) (Extended Data Fig. 6a,b). Increasing average wind speed increased cumulative IPD risk (positive association), while increasing ozone concentrations (ozone increases with increasing temperatures) decreased cumulative IPD risk, concordant with decreasing risk in the summer months (Extended Data Fig. 6c,d and Supplementary Fig. 2).

We estimated the risk of disease above the 2016–2029 National Ambient Air Quality Standards (NAAQS) thresholds in South Africa^22^ for PM_2.5_ (40 μg m^−3^), PM_10_ (75 μg m^−3^) and SO_2_ (125 μg m^−3^) (Supplementary Tables 5 and 6). To account for meteorological confounding in air pollution models, we evaluated multiple formulations and lag structures of temperature and humidity. The final air pollution models were adjusted for mean temperature in the same week as the pollutant and absolute humidity at lag 3 (3 weeks earlier) (Supplementary Fig. 3 and Tables 4 and 7). PM_2.5_, PM_10_ and SO_2_ all had positive associations with IPD (Fig. 2c and Extended Data Fig. 5).

**Extended Data Fig. 7:**
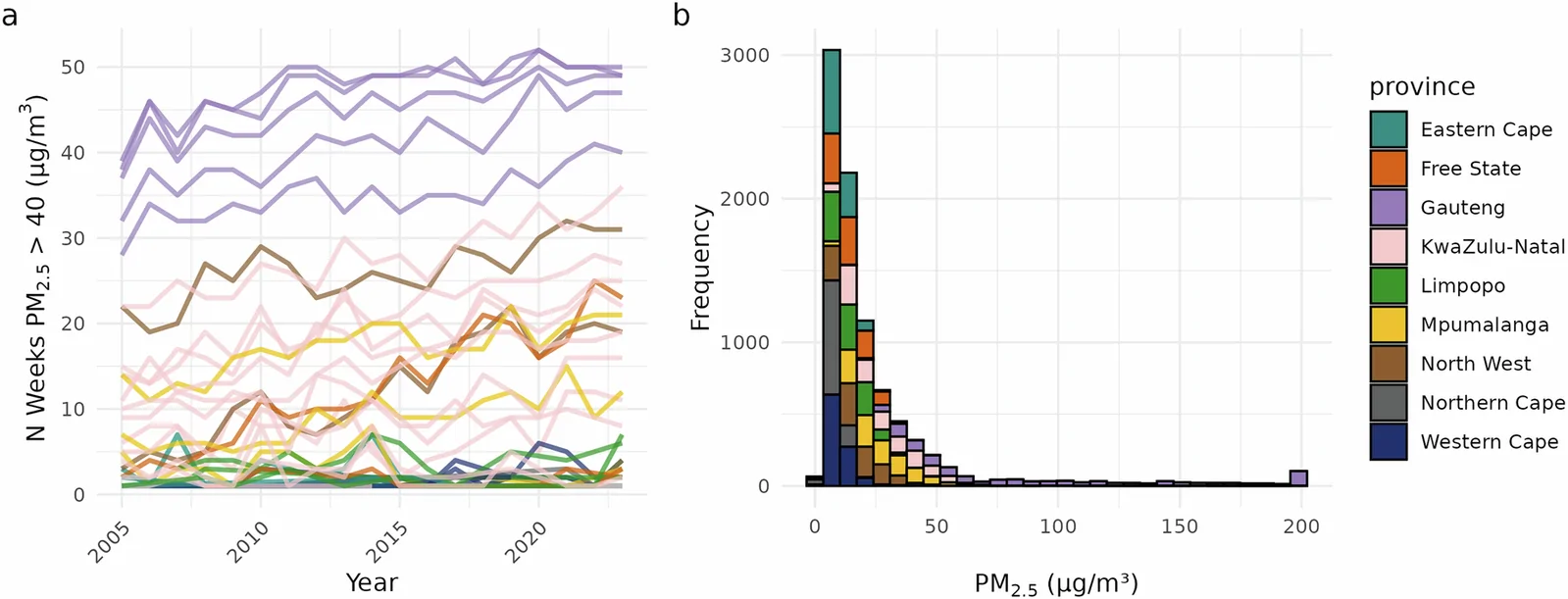
Particulate matter < 2.5 μm distributions across districts. Particulate matter < 2.5 μm distributions (**a**) weeks with >40 μg/m^3^ (the NAAQS threshold for PM_2.5_) per year and district colored by province across the time period. The grouping is by district whereby in Western Cape (dark blue) and Northern Cape (grey) have no weeks exceeding the threshold. Eastern Cape (blue) and Limpopo (green) only have districts with <10 weeks (blue), Free State includes Fezile Dabi (orange), Gauteng includes the City of Johannesburg, City of Tschwane, Ekurhuleni, Sedibeng, and West Rand (purple), KwaZulu-Natal includes Amajuba, eThekwini, Harry Gwala, iLembe, Ugu, uMgungundlovu, uMzinyathi, and uThukela (pink), Mpumalanga includes Gert Sibande and Nkangala with >10 weeks exceeding threshold (yellow), North West includes Bojanala Platinum and Dr Kenneth Kaunda with > 10 weeks (brown) (**b**) histogram of particulate matter distribution across provinces. Both (**a-b**) colored by province. Source data

With a weekly average of 50 µg m^−3^ of PM_2.5_, there was a 3.4% 8-week cumulative increased risk of IPD (cumulative RR 1.03: 95% CI 1.02–1.05). Strikingly, there was an 18% increased IPD risk (cumulative RR 1.18: 95% CI 1.12–1.23) with a weekly PM_2.5_ of 100 µg m^−3^ (Fig. 2c). The risks from high (100 µg m^−3^) concentrations of PM_2.5_ were immediate (lag week 0 RR 1.02: 95% CI 1.01–1.04) and persisted for 6 weeks (Extended Data Fig. 5). These patterns were robust to models fitted to Gauteng province alone, and nationally excluding Gauteng (Fig. 2a, and Supplementary Figs. 4–7 and Table 4). We also explored the potential for fine-scale spatiotemporal confounding of the PM_2.5_ effect by allowing spatial effects to vary by year. This showed high correlation with the interannual province-level effects from the sociodemographic base model, with a few urban districts as outliers. However, the PM_2.5_ exposure–response relationship was robust to this analysis, indicating that fine-scale spatiotemporal confounding was not influential (Supplementary Fig. 8). The city of Johannesburg regularly exceeded weekly averages of 100 µg m^−3^ of PM_2.5_ (32 weeks in 2023) and across South Africa, the weekly PM_2.5_ concentration was at least 50 µg m^−^^3^ in ~23 districts a year (14 weeks per district average) (Extended Data Fig. 7 and Supplementary Table 4). In 2019, Gauteng province had a mean PM_2.5_ concentration of 117 µg m^−3^ and 718 observed IPD cases. The population attributable fraction, based on weekly PM_2.5_ concentrations was 2.19%. This implies that if PM_2.5_ had been maintained at or below 40 µg m^−3^, 16 of the 718 cases could theoretically have been averted; if reduced to the more stringent WHO daily threshold of 15 µg m^−3^, 24.8 cases (3.45%) would have been averted.

A weekly PM_10_ concentration of 100 μg m^−3^ increased the cumulative 8-week risk of disease to 5.6% (cumulative RR 1.06: 95% CI 1.04–1.07) and had a lower acute risk (lag week 0) than for PM_2.5_ (Extended Data Fig. 5). With a weekly SO_2_ concentration of 150 μg m^−3^, the risk of IPD began only 3 weeks after exposure and remained persistent up to 8 weeks after exposure (weekly SO_2_ of 150 μg m^−3^ cumulative RR 1.04: 95% CI 1.03–1.06) (Extended Data Fig. 5, and Supplementary Figs. 7 and 16).

### Risk from PM_2.5_ varies by age, disease and pneumococcal serotype

To assess the varying impact of air pollution across outcome types, we stratified IPD outcomes by particular demographic groups, disease outcomes and pneumococcal serotypes. We employed the same air pollution model formulation described above, which included the sociodemographic base model with meteorological confounders (temperature and humidity), and a DLNM for PM_2.5_, PM_10_ and SO_2_, respectively. We focus here on PM_2.5_ due to its majority anthropogenic sourcing in South Africa^23–25^ and clear positive association with pneumococcal disease.

**Fig. 3:**
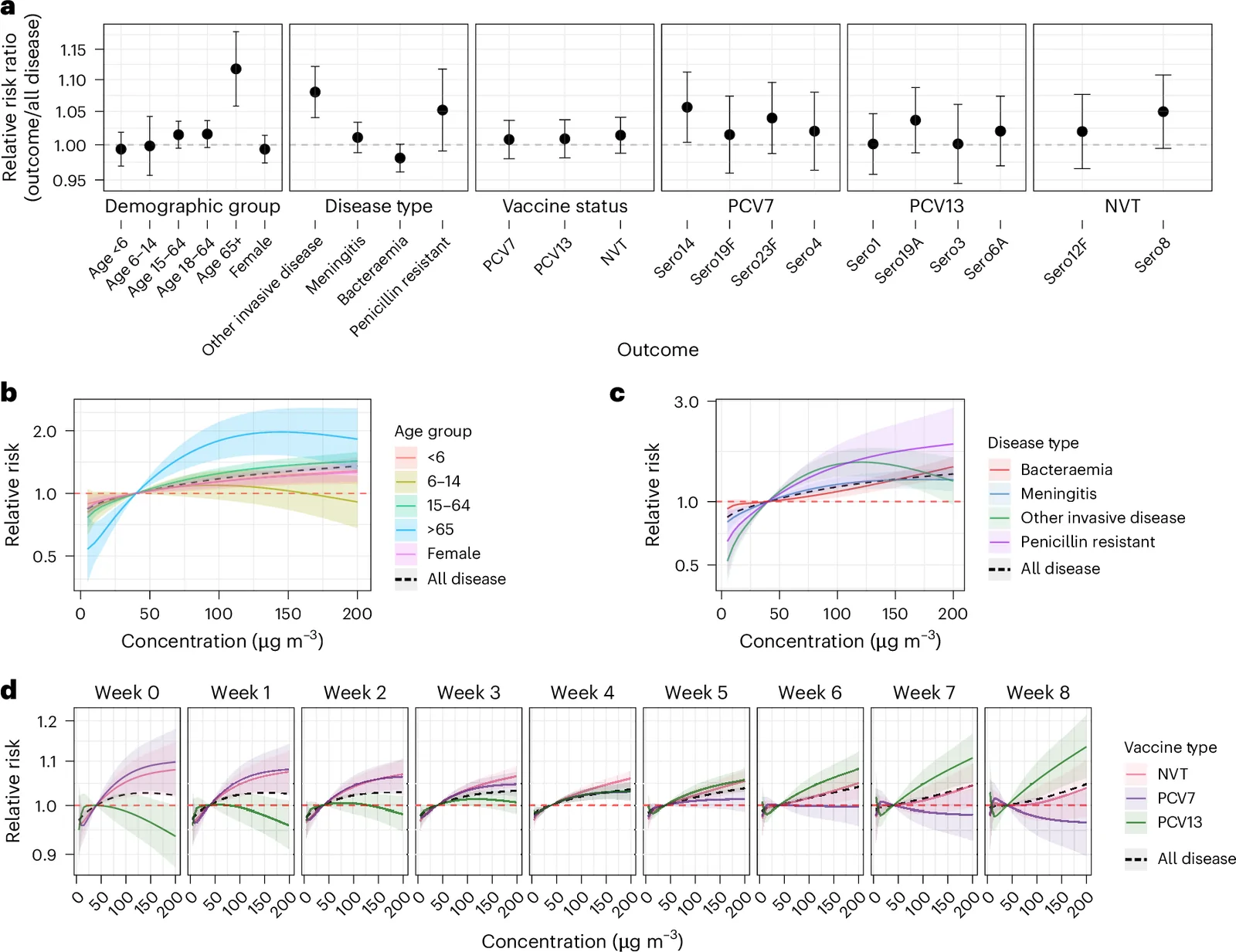
Risk of specific sub-outcomes of IPD given weekly averages of particulate matter <2.5 μm (PM_2.5_) in the air pollution model framework. These models were all fitted to case data at the district level from 2005 to 2019. **a**, Relative risk ratio (RR) of each specific model compared to the model including all disease at 50 μg m^−3^ (exceeding the NAAQS threshold for PM_2.5_ by 10 μg m^−3^) where point estimates indicate the ratio of the median outcome-specific cumulative RR against the reference model median. Error bars represent 95% confidence intervals, derived from pooled, back-calculated standard errors assuming independence between models. **b**, Relative risk across age groups including age ≤5 (red), age 6–14 (light green), age 15–64 (green), and age 65 and older (blue), and including only females (purple). **c**, Relative risk of PM_2.5_ across disease types for bacteraemia (red), meningitis (blue), other invasive disease types (green), penicillin-resistant infections (purple) and all disease (black). Weekly lag times for **b** and **c** are available in Supplementary Fig. 9. **d**, Models for disease caused by serotypes targeted by PCV7 (purple), additional 6 serotypes in PCV13 (green) and NVT (red) with a 0–8-week lag time from PM_2.5_ to disease for each vaccine type group (pcv7, purple; pcv13, green; nvt, pink). Cumulative vaccine type exposure–response curves are given in Supplementary Fig. 10. In **b**–**d**, the RRs from the model including all disease cases (black dotted) are included; shading and error bars represent 95% credible intervals, and point estimates represent the median. Numbers of case counts per outcome group are reported in Supplementary Table 2. Source data

The highest risk from PM_2.5_ was for those aged ≥65 years, with a cumulative RR for 50 μg m^−3^ weekly average PM_2.5_ of 1.16 (95% CI 1.10–1.22) (Fig. 3b). There was a 12% higher risk for this age group compared with the main model (all IPD) (Fig. 3a). PM_2.5_ had a more acute impact (RR > 1 at early lag weeks) for those between the ages of 15 and 64, with risk increasing in the same week as high PM_2.5_ concentrations (lag 0), while all other demographic groups showed similar lag patterns with the highest risk 3–4 weeks after exposure. This may reflect the higher prevalence of HIV among 15–64-year-olds^26^. The risk for children ≤5 years old was not negligible, with an 8-week cumulative RR of 1.03 (95% CI 1.01–1.05) at 50 μg m^−3^ weekly average PM_2.5_, which, while similar to the average risk of IPD across the population, translates to 3% more children with IPD at high pollution levels. This highlights that PM_2.5_ does not only pose an increased disease risk for adults (Supplementary Fig. 9). Models for particular disease outcomes (bacteraemia, meningitis, other invasive disease and penicillin-resistant disease) had similar risk patterns with exposure to weekly average 50 μg m^−3^ PM_2.5_. However, there was a higher cumulative risk (RR 1.12: 95% CI 1.08–1.16) and a more acute risk for other invasive disease types (such as pericarditis or empyema) (Fig. 3c and Supplementary Fig. 9). The risk with a 50 μg m^−3^ weekly average was higher for meningitis (RR 1.05: 95% CI 1.03–1.06) than for bacteraemia (RR 1.01: 95% CI 1.00–1.03) (Fig. 3c), possibly indicating different mechanisms of effect across IPD types. Penicillin-resistant IPD had a 5% higher risk (RR ratio 1.05: 95% CI 1.00–1.12) than the main model (Fig. 3a), with an 8.9% risk (RR 1.09: 95% CI 1.03–1.15) at 50 μg m^−3^ weekly average PM_2.5_ (Fig. 3c).

**Extended Data Fig. 8:**
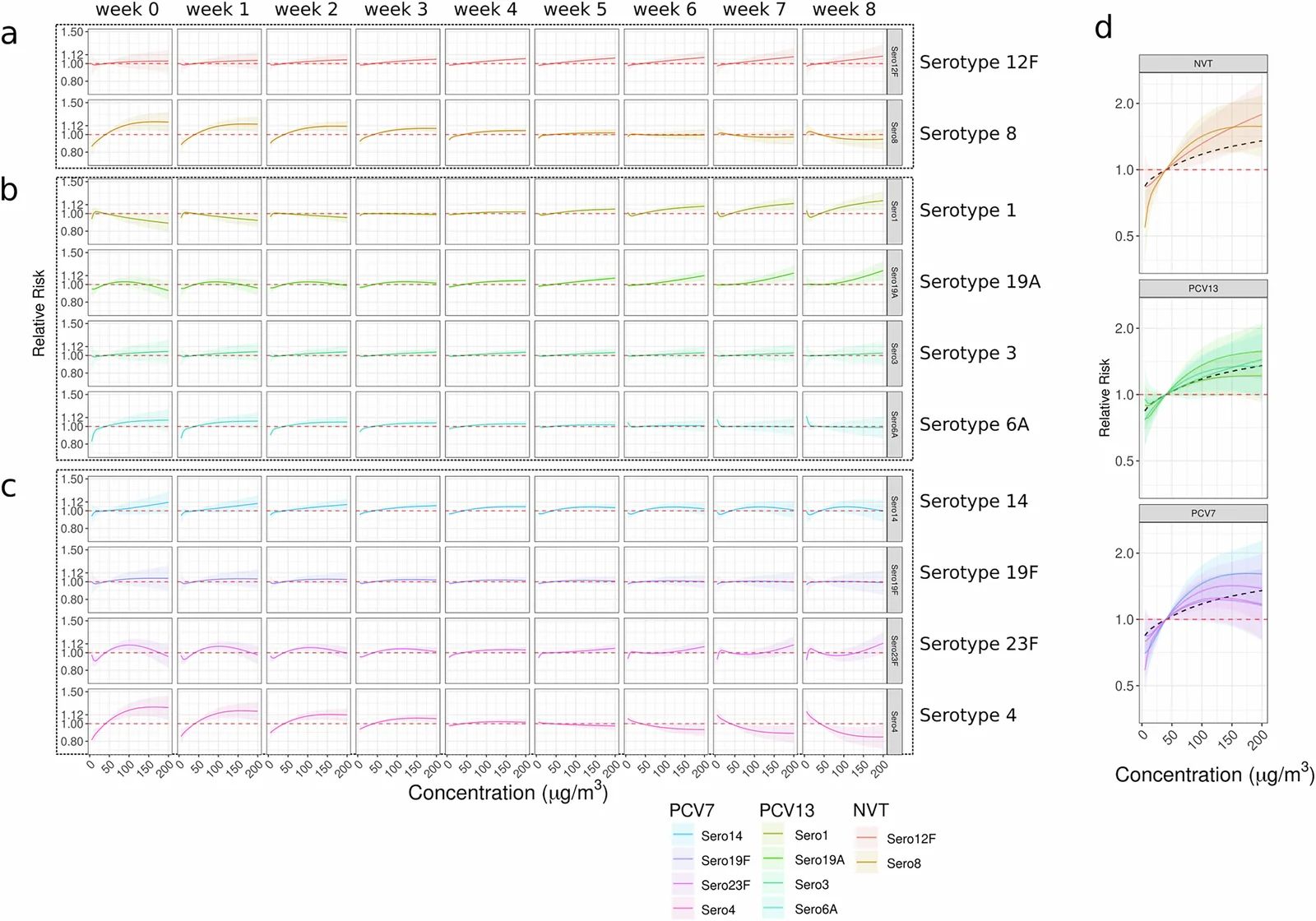
**Exposure-response curves across different weekly exposure to PM**_**2.5**_ **(concentration μg/m**^**3**^**) including serotype stratifications of invasive disease including** (**a**) NVTs serotype 12 F and serotype 8, (**b**) PCV13 serotype 1, serotype 19 A, serotype 3, serotype 6 A, and (**c**) PCV7 serotype 14, serotype 19 F, serotypes 23 F, and serotype 4. (**d**) includes the cumulative 8-week relative risk for each of these serotype models grouped by vaccine type. The legend is shared across all figures. The shaded error bars represent 95% credible intervals and the point estimates indicate the median. Source data

The cumulative risk from PM_2.5_ across all vaccine types was similar across the 8 weeks; there was a varied lag pattern with an immediate effect for NVTs and PCV7 serotypes but a longer time to effect for the additional serotypes in PCV13 (Fig. 3d). These effects persisted when we stratified by pre- and post-PCV periods, implying a difference in mechanism beyond vaccine associated changes in serotype proportions (Supplementary Fig. 10a). Serotype-specific stratification revealed distinct dynamics: we found acute risks from PM_2.5_ for NVT serotype 8 (94% GPSC3 in this dataset), and PCV13 serotypes 23F (68% GPSC14) and 4 (58% GPSC70) (Extended Data Fig. 8). In addition, we found a greater 8-week cumulative risk for some serotype-specific IPD than from the main model. With 50 μg m^−3^ average weekly PM_2.5_ concentration, there was a higher risk of IPD with PCV7 serotype 14 (RR ratio 1.06: 95% CI 1.00–1.11; 49% GPSC10), PCV13 serotype 19A (RR ratio 1.04: 95% CI 0.99–1.09; 92% GPSC17) and NVT serotype 8 (RR ratio 1.05: 95% CI 0.99–1.11; 94% GPSC3) (Fig. 3a, Extended Data Fig. 8a–c, and Supplementary Tables 8 and 9). These results underscore the sensitivity of some invasive serotypes to air pollution^5,27^.

### Timing of air quality effects is modified by lineage diversity

We estimated the impact of PM_2.5_ on IPD at the province level according to particular GPSC-lineage prevalence, accounting for an 8-week lag time for the years 2005–2019. In these models, we included IPD as the outcome and incorporated genomic lineages into the model (*n* = 4,350) by including the proportion sequenced at each space–time increment as an effect modifier (Supplementary Fig. 11). We interacted the cross-basis for each environmental factor with the proportion of each dominant genomic lineage (*n* ≥ 100 and GPSC8) among sequenced isolates (Supplementary Fig. 12 and Table 2). Lineage diversity interaction effects improved goodness-of-fit metrics (Supplementary Fig. 13 and Table 4).

**Fig. 4:**
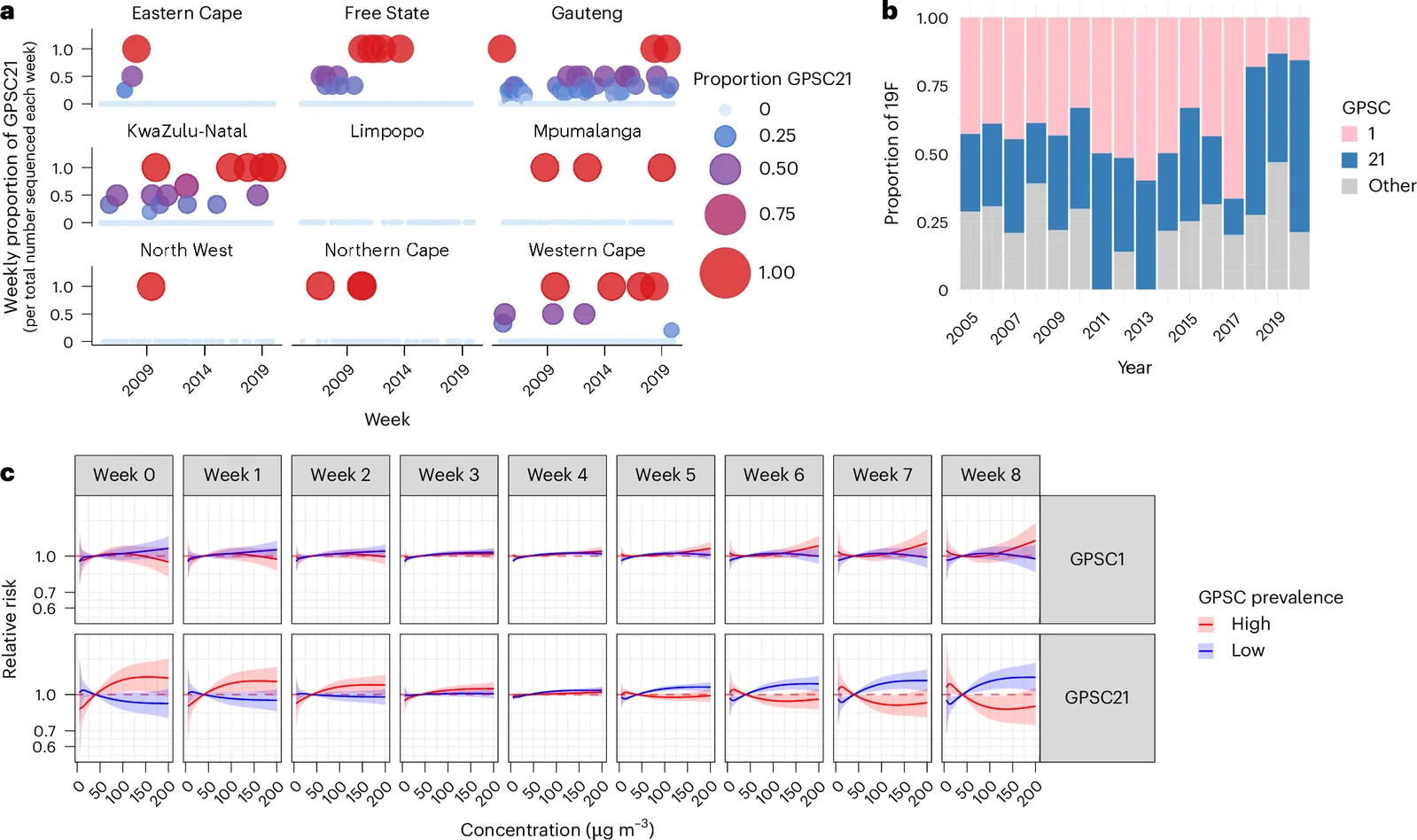
Incorporating GPSC21 by modifying the effect of PM_2.5_ on IPD in the air pollution model framework. **a**, Proportion of sequences that were GPSC21 in each week and province per total number sequenced each week. Where less than half (0.50) of the total sequenced per week and province were GPSC21 is indicated with a smaller circle size (blue shade) and greater than half (0.50) are indicated with a larger circle size (red). **b**, Proportion of serotype 19F isolates found on each GPSC per year, highlighting GPSC1 (pink) and GPSC21 (blue). **c**, Exposure–response curves for the relative risk of province-level IPD across concentrations of PM_2.5_ for GPSC1 (top) and GPSC21 (bottom), including curves for high prevalence (red) and low prevalence (blue) of each lineage. Shading represents 95% credible intervals and point estimates represent the median. Source data

**Extended Data Fig. 9:**
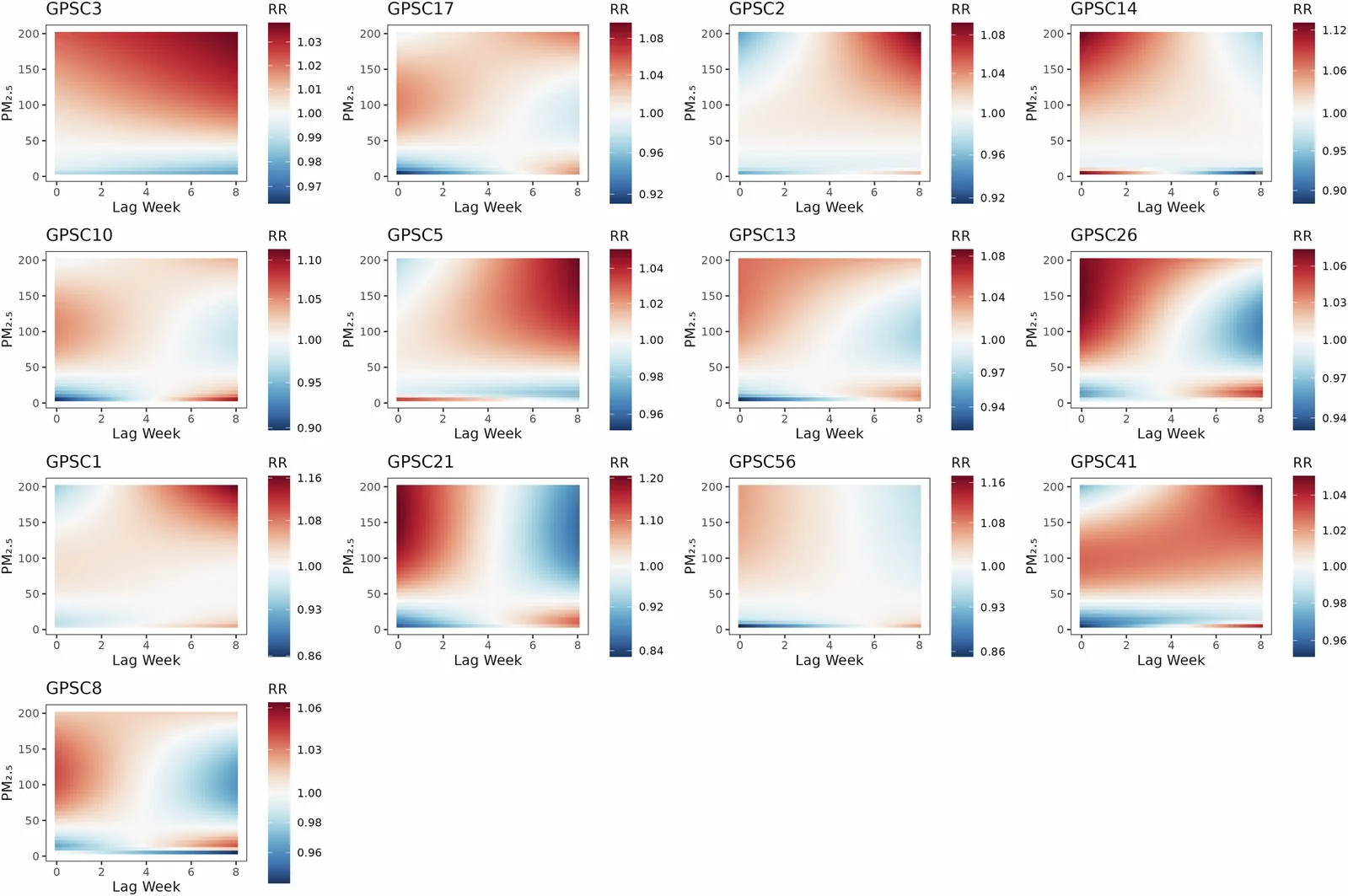
**Median relative risk (RR) surfaces from the air pollution models.** where risk >1 is red and <1 is blue with consistent limits across RR’s, for different concentrations of PM_2.5_ concentration μg/m^3^ (y-axis) and 8-weeks of lag (x-axis) when there is high prevalence of each of the GPSCs in order of total count across the time period. Source data

We explored the risk of IPD given the GPSC prevalence across concentrations of PM_2.5_ (where prevalence is across quantiles of GPSC proportions over the entire time period; low=0.1 and high=0.9) (Fig. 4a and Supplementary Table 10). While the 8-week cumulative effect did not vary with lineage diversity, there was variation in risk across lags and GPSCs (Supplementary Fig. 13a,b and Extended Data Fig. 9). There was an acute effect for GPSC21 whereby the risk of IPD was elevated given the high prevalence of these lineages. The cumulative effect with a GPSC21 interaction also had different exposure–response curves between high and low prevalence (Fig. 4c). We found an immediate effect whereby locations with a high prevalence of GPSC21 had an acute risk of IPD in the same week as high concentrations of PM_2.5_, whereas times and locations with low prevalence of GPSC21 had an increased risk starting at 5 weeks after the exposure (Fig. 4c). In this dataset, GPSC21 comprised serotype 19F (*n* = 90) with one instance of 19A in 2005. Serotype 19F is targeted by PCV7 and is characterized in South Africa by an initial GPSC1 lineage (*n* = 114) which decreased post PCV, when GPSC21-19F became predominant (Fig. 4b)^5^. GPSC21 interaction models consistently modified PM_2.5_ effect timing (Supplementary Fig. 14). Although the mechanism remains unclear, circulating lineage diversity evidently plays a role in environment–disease relationships.

## Discussion

In this study, we applied spatiotemporal Bayesian hierarchical models to investigate the relationship between meteorological factors, air pollution and IPD risk. We identified complex, delayed and nonlinear effects from meteorological factors and heterogeneous effects from PM_2.5_ across demographic groups, disease outcomes and serotypes. Genomic lineage prevalence modified the PM_2.5_–IPD association; specifically, we found a heightened, short-lag PM_2.5_ effect when GPSC21-19F had high prevalence. Our study corroborates previous evidence for increased risk at lower humidity, temperature and precipitation, contributing to a growing pool of evidence across sociodemographic and climatic regions^12^. Temperature effects were nuanced: higher temperatures conferred immediate risk while cold effects were delayed, possibly reflecting differing mechanistic relationships with IPD. Effect sizes for temperature and air pollution were modest (below 10%) while cumulative humidity effects were larger (25% risk reduction). We confirmed a positive relationship of PM_2.5_, PM_10_ and SO_2_ with IPD.

We observed the most immediate effect from PM_2.5_ in individuals aged 15–64 years, while the cumulative effect was greatest for those ≥65 years old. These patterns are consistent with previous studies demonstrating heightened susceptibility among older adults^16,28,29^, possibly reflecting cumulative exposure and reduced lung function^16,30^ triggering increased risk for IPD from PM_2.5_. We found no differences with sex stratification, although the lack of household air pollution data may mask gendered effects^31,32^.

We identified heterogeneous effects of PM_2.5_ across serotypes, with immediate increases in risk for some (including NVT serotype 8 and PCV serotypes 4 and 23F) and lagged effects for others. This may reflect differences in colonization and invasion dynamics across serotypes^33^. These patterns could be partly explained by vaccine-driven shifts in carriage. Serotypes with higher population prevalence may respond immediately to air pollution exposure, whereas those with reduced post-vaccine carriage (PCV13 types) may exhibit delayed risk^5,34,35^. Mechanistically, the effect of PM_2.5_ on IPD is probably related to both the host (for example, increased susceptibility to colonization^36^, decreased mucociliary clearance^37^) and the bacterium (for example, increased transformation capacity^38^ and biofilm formation)^17,39^. Previous studies have identified increased epithelial adhesion, biofilm formation and bacterial burden with air pollution^30,33,36,37^, while others found a decrease in carriage acquisition with increasing PM_2.5_ concentrations^13,39^. The acute effects observed for serotype 4, 19A and 23F suggest divergent serotype-specific invasion pathways. For example, the persistence of serotype 4 in adult IPD^5,40,41^ may reflect unique environmental sensitivity driven by its prevalent *psrP* virulence gene, which could interact with PM_2.5_-induced microabrasions^42^ to accelerate invasion with minimal lag time^41,42^.

GPSCs also have different carriage durations and demographic propensities^4,6^. GPSC21-19F is a persistent vaccine-targeted strain in South Africa and its acute response to PM_2.5_ could explain its persistence. Serotype 19F is especially prevalent among 5–18-year-olds and behavioural differences may contribute to the acute effects of PM_2.5_ on GPSC21 IPD^5^. Direct experimental evidence linking lineage-specific data to PM_2.5_ would be helpful to further elucidate the environmental–GPSC21 pattern we find here, alongside interrogating the effect in other geographic regions with this lineage.

These heterogenous effects from PM_2.5_ are consistent with known variation in pneumococcal virulence, carriage dynamics and host susceptibility across serotypes and lineages^4,7,43^_._ Our results suggest that high air pollution favours certain serotypes and lineages, supporting the premise that environmental factors can confer microbial fitness advantages. This relationship is further shaped by the inextricable, bidirectional link between PM and climate change, whereby particulate matter influences atmospheric chemistry, while climatic shifts affect the formation and distribution of PM^44^. In high-exposure regions, such as the Gauteng province in South Africa where the concentrations of PM_2.5_ and PM_10_ are extremely high, these environmental pressures are particularly acute. The current daily threshold aim for PM_2.5_ in South Africa is 40 μg m^−3^ whereas the WHO threshold is 15 μg m^−3^. We found in the year 2019 that 15.7 cases might have been averted if the South African threshold was met (24.8 cases averted by meeting the WHO threshold). More stringent air pollution goals could directly mitigate IPD.

This study provides microbial diversity-aware estimates of environmentally driven IPD risk, demonstrating that the risk is not uniform across host or microbial populations. These relationships should be studied across different regions with varied climatic conditions and demographic structures. To improve our understanding of how environmental factors affect different serotypes or lineages, stratified epidemiological analysis and mechanistic experiments are needed to define the biological interactions between microbial diversity, host susceptibility and the environment. The time period, high spatial resolution and extensive metadata of our study is an asset for robust space–time ecological modelling studies. Our approach could be leveraged for other infectious diseases to incorporate microbial genomic data into environmental epidemiological models.

**Extended Data Fig. 10:**
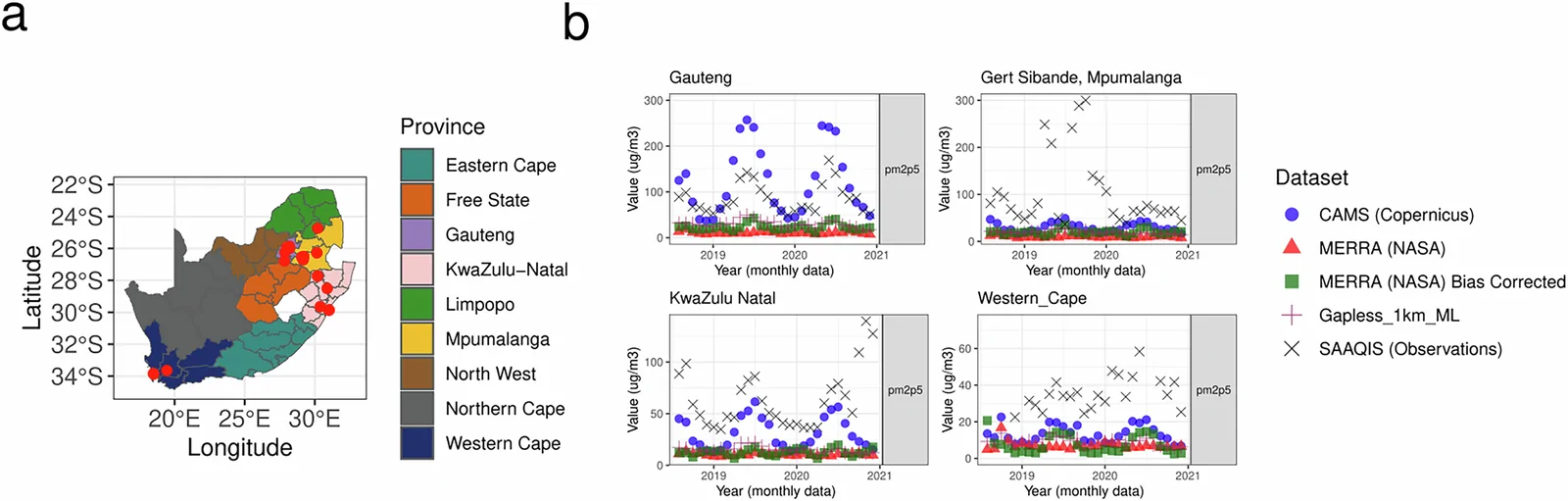
Air pollution reanalysis data compared to observations in South Africa. (**a**) Map of air quality monitoring stations included from SAAQIS. Colored by province with lines distinguishing districts. Locations of observation stations are indicated in red dots. (**b**) The concentration of weekly PM_2.5_ in μg/m^3^ from Gauteng across reanalysis products from Copernicus (CAMS), MERRA-2 from NASA, MERRA-2 with a bias adjustment and a machine learning gapless product at 1 km grid squares. Source data

The reanalysis and observational datasets for PM_2.5_ are imperfect. Our study highlights the critical need for regional bias-corrected models to accurately assess the impact of air pollution on infectious diseases globally (Extended Data Fig. 10). Our use of South African NAAQS baselines and weekly means may underestimate air pollution risks relative to more stringent WHO 24-hour standards. While the lack of granular indoor air pollution data is a limitation, the strong correlation between indoor and ambient pollutants suggests that our results still capture the primary exposure–outcome association^31,45^. Furthermore, we used proxy variables (for example, population density and spatiotemporal random effects) to account for unobserved risks from indoor household factors or extreme weather events such as flooding and associated population changes^46^. Subdistrict-level models could better elucidate these fine-scale effects.

Despite our inclusion of the entire GERMS-SA dataset across the time periods, sources of ascertainment bias include access to care and accurate processing of specimens. Our data are from hospital locations, so with long-distance travel to hospitals, cases may be attributed to a spatial grid different from the patient’s home location. While audits were conducted to capture unreported cases, these excluded serotype data, and the genomic sequences (while encompassing a large proportion) did not encompass the entire epidemiological dataset. We did not include carriage isolates in this analysis, as the focus of GERMS-SA is on invasive disease. Consequently, our results pertain only to IPD, excluding non-bacteraemic pneumonia. This exclusion may be skewing the impact of some of our environmental associations as a whole. Understanding the relationships between environmental factors and bacteraemic versus non-bacteraemic pneumonia is an important direction for future work.

In summary, environmental factors, specifically temperature, humidity and air pollution, influence IPD risk in South Africa. We identified delayed and nonlinear associations which vary by serotype or lineage. While PM_2.5_ was associated with IPD overall, the highest risk is among those ≥65 years old, and there is increased risk for particular serotypes with variable exposure–response patterns. The variable lag effects suggest that the biological mechanisms driving PM_2.5_-related risk differ given the underlying microbial population and sociodemographic factors. Bacterial populations respond non-uniformly to the environment. Our findings suggest that climate change and urbanization may shift IPD dynamics. Meeting established air pollution policy thresholds remains a critical priority to protect human health.

## Methods

### Data processing

#### Pneumococcal disease datasets

##### Epidemiological cases

The case counts of invasive pneumococcal disease (IPD) were provided by the National Institute for Communicable Diseases (NICD) in South Africa as part of national surveillance encompassing all nine provinces (GERMS-SA). We included the entire dataset which comprised culture-positive for pneumococcus with viable isolates available for further analysis, culture-positive with no viable isolates available and culture-negative with metadata results from PCR. Isolates were phenotypically characterized by the Quellung method using serotype-specific antisera (Statens Serum Institute, Copenhagen, Denmark) as previously described^5,47^, with >97% concordance with in silico typing for all serotypes of focus (PCV7 serotypes 14, 19F, 23F, 3; PCV13 serotypes 1, 19A, 3, 6A; NVT serotypes 12F and 8)^48^ (Supplementary Table 9). We utilized the phenotypic serotypes for our analysis. Antimicrobial resistance was predicted from the genomes utilizing the CDC pneumococcal typing pipeline for the detection of resistance determinants and in silico minimum inhibitory concentration (MIC) predictions^5,47^. Penicillin resistance was determined using the meningitis MIC cut-off value^49^. Finally, these included pneumococcal disease lines list cases (*N* = 59,017) between 2005 and 2023 collected from 531 hospitals across 52 districts in South Africa (Fig. 1a,b). These excluded *n* = 661 cases from the original dataset without hospital geolocation. Most cases were found in Gauteng province with 24,303 cases. Northern Cape reported the least number of cases, with 1,067 (Supplementary Fig. 1c). Disease types included 32,639 bacteraemia (55.3%), 21,167 cases of meningitis (35.9%) and 5,211 other IPD cases (8.8%), such as empyema, septic arthritis, endophthalmitis, peritonitis or pericarditis. These were classified given specimen type whereby meningitis cases were isolated from cerebrospinal fluid specimens, bacteraemia from blood specimens, and other invasive disease classes were from other typically sterile sites. Bacteraemic cases include bacteraemic pneumonia but not non-bacteraemic pneumonia. The median age was 32 years with 13% of cases reported in children <1 year old. There was missing age data for 4.3% of cases and serotype information was missing for 37.8% of cases (Fig. 1c). We included data from 2005 to 2023 in concordance with the environmental datasets for the initial model testing. As the genomic dataset ran until 2020, the year marking the beginning of the COVID-19 pandemic (with a substantial decrease in IPD counts), we restricted the models to the period 2005–2019 (*n* = 52,437) so as to overlap with epidemiological cases and exclude this case reduction. In the overall environmental epidemiology models, we included all data; for the model stratifications by outcome, we included all reported data for each outcome. The outcomes we assessed included different age groups, disease types and serotypes.

##### Genomic sequences

The genomes included in this study were sequenced as part of the Global Pneumococcal Sequencing project, GPS^50^, which is a global genomic survey of *S. pneumoniae*. Isolates were collected by the NICD in South Africa from 2005 to 2020 (*n* = 4,912) from 48 of the 52 districts. We only included genomes through the year 2019 (*n* = 4,350). Isolates were randomly sampled using a previously described protocol whereby ~300 invasive-disease isolates from each year (2005–2014) were selected with a specific target age breakdown (50% from <3-year-olds, 25% from 3–5-year-olds, 25% from >5-year-olds) in the first phase. In the second phase of sequencing, 100 disease isolates from 2005 to 2010 from children <5 years old were additionally randomly sequenced. The sampling for 2015–2020 was similar to phase 1, but the sampling strategy was not adjusted for the reduction in IPD cases following PCV (thus included more >5-year-olds)^5,8,51^. Most sequences are from the districts with the highest population sizes (Supplementary Fig. 11). By incorporating the genomic lineages as a modifying proportional interaction effect, and normalizing this by the total number of sequences in each space–time increment, we attempted to limit any potential biases associated with their selection. Assembly quality control parameters included a minimum average sequencing depth of 20× and an assembly length of 1.9–2.3 Mb. Sequences with more than 220 heterozygous single nucleotide polymorphic (SNP) sites were excluded. The genomes were assembled using Spades assembly software^52^ and the gene annotations were made using Prokka^53^. PopPunk^2^ was used to assign each genome to a global pneumococcal sequence cluster (GPSC). We included each GPSC with an *N* > 90 and included GPSC8 due to its public health relevance^5^. We included the proportion as the number of each GPSC over the total number of sequences per week per province (Supplementary Fig. 12).

##### Sociodemographic data

The sociodemographic data were extracted from multiple sources. Annual district-level population estimates for the period 2005–2023 were obtained from Statistics South Africa mid-year population estimates (Statistical Release P0302). Specifically, we utilized the district municipality population datasets generated from the 2023 projection series, which applies demographic assumptions (births, deaths and migration) to project forward from the 2022 South African National Census baseline while retrospectively modelling historical annual trends^54,55^. From the same report we included per province ART coverage, HIV prevalence, life expectancy, growth rate, % children (<15) and the % elderly over time (60+). We also included the aging index and dependency ratio. These are defined by Statistics South Africa as the number over age 65 per 100 youths and the number <15 years old combined with the number 60+ over the number of working-age population (15–64), respectively^55^. PCV vaccination has a 2 + 1 schedule in South Africa and was first introduced in 2009 (PCV7: serotypes 4, 6B, 9V, 14, 18C, 19F, 23F), replaced by PCV13 (additional serotypes 1, 3, 5, 6A, 7F, 19A) in 2011 (Supplementary Table 1). PCV coverage for 1, 2 and 3 doses was included from the WHO Immunization Data report. ‘Three doses’ is defined as the percentage of surviving infants who receive the 3rd dose of PCVs. We included the official coverage estimates as well as the WHO/UNICEF Estimates of National Coverage (WUENIC)^56^ (Supplementary Fig. 1d). The official estimates are initially based on surveys and other administrative messages, which, due to biases and inaccuracies, are adjusted by the national authorities to provide estimates of the most likely coverage.

#### Environmental datasets

South Africa comprises temperate and arid climates according to Köppen classification^57^. Gauteng and Western Cape provinces (where the majority of our case data comes from) are subtropical highland and Mediterranean climates, respectively.

##### Meteorological data

The environmental datasets spanned 2005–2023 (inclusive). We included temperature, precipitation, relative and absolute humidity metrics from the global reanalysis dataset ERA5-Land^58^ aggregated for weekly time points at the district level. This is gridded data with 0.1° resolution (accounting for the mean latitude of South Africa at −29°, so each grid square is ~108 km^2^). This resulted in a mean of 218 grid cells per district, ranging from 15 in Johannesburg, Gauteng, to 1,180 in Namakwa, Northern Cape. Daily temperature metrics were retrieved and aggregated to weekly, such that we included the weekly mean of the daily mean, maximum or minimum temperature. Precipitation was initially retrieved as hourly average metres (m) per second precipitation loss rate (prlr). We converted it to daily millimetres (mm) and then tested inclusion of the weekly total precipitation and the mean daily precipitation.

We calculated the standardized precipitation and evapotranspiration index (SPEI) at 3-month and 6-month time periods using the above-mentioned precipitation and temperature data from ERA5-Land. The packages used for this calculation were SPEI^59^, ClimProjDiags^60^, CSindicator^61^ and CSTools^62^.

Absolute humidity (absh) is defined as the mass of water vapour in a volume of air (in g m^−3^). Relative humidity (hurs) is the water vapour present in the air relative to saturated air and defined as the partial pressure of water vapour as a percentage of the saturation pressure (%). Differences in apparent effect magnitude between absolute and relative humidity should be interpreted cautiously, as they depend on the distribution of each variable (Fig. 1d).

##### Air pollution data

The air pollution datasets spanned 2005–2023 (inclusive). To determine which estimates of air pollution to include, we compared air pollution estimates obtained from the Copernicus Atmosphere Monitoring Service (CAMS) global reanalysis dataset and NASA’s MERRA-2 (refs. ^63,64^). We performed an assessment of the correlation between the gridded air quality reanalysis models against observational data made available by the South African Air Quality Information Services (SAAQIS)^65^. PM_2.5_ and PM_10_ are not directly included in MERRA-2, so they were computed using methods detailed in the MERRA-2 documentation^66^ (Fig. 1d and Supplementary Table 5).

We included the MERRA-2 product from NASA for the entire time period and also included the bias-corrected MERRA-2 product from 2020 to 2023^67^. The CAMS global datasets are at ~80 km^2^ resolution while the MERRA-2 datasets are at 50 km^2^ spatial resolution. We also included a gapless 1 km gridded machine learning model from ref. ^68^.

We extracted observational data from air quality monitoring stations provided by SAAQIS in South Africa. We calculated the mean absolute error, root mean square error and Spearman’s correlation between the reanalysis products and the mean across observations in the district of Gert Sibande, Mpumalanga, Gauteng province and KwaZulu-Natal province (Extended Data Fig. 10a). These were the locations with the most-consistent observations across the time period available in SAAQIS. We performed a formal comparison of the datasets against observational data and found that CAMS was the best-performing reanalysis model (Extended Data Fig. 10b). CAMS performed best despite its poor performance in the Gert Sibande region where none of the products performed well, either due to the observations being flawed, or a bias across all reanalysis products impacting this region. We included the air quality reanalysis data from CAMS in our study (Supplementary Table 6).

As we demonstrate in our comparison, air quality reanalysis models do not correlate well with the observational data across all sites. The available observational data from SAAQIS across this time period are sparse, which limits our ability to select an ideal reanalysis product. The correlation between the observations and CAMS was better than that with MERRA-2 and other bias-corrected datasets. The absence of reanalysis models which are bias corrected to this region and the lack of observational data points for many less-urban regions highlight the limitations of addressing the environmental impact of high-burden infectious diseases in low-income settings with uncorrected environmental data^69,70^. To improve understanding of the environment’s impact on high-mortality infectious diseases, efforts must be made to improve air pollution global reanalysis models^71^. In addition, we used weekly means rather than the 24-hour means for air quality metrics, so these thresholds are lenient (for example, if 1 day within a 7-day period has a 24-hour concentration exceeding the threshold but the other 6 days do not, the weekly mean concentration will likely be below the threshold).

The R package startR was used to retrieve and reformat all multidimensional environmental datasets^72^.

### Epidemiological model specification

#### Bayesian hierarchical mixed models using integrated nested Laplace approximation

In brief, here we summarize our modelling strategy for identifying meteorological and air pollution factors that drive patterns of pneumococcal disease, with an in-depth analysis of the effects of PM_2.5_ on different outcomes (for example, serotype, age group, disease type).

To assess the risk from environmental factors on IPD, we adopted the following modelling strategy*:*

1. We first developed a sociodemographic base model which accounted for population density and vaccination period as well as residual seasonal, spatial and interannual effects.
2. We tested different metrics for vaccination and antiretroviral therapy to assess their impact on disease patterns. We chose a sociodemographic base model given a balance between model fit and tractable complexity, ultimately including confounding effects of vaccination as a categorical variable (across 3 vaccination periods), and population density as a continuous variable, alongside the residual random effects.
3. We then developed a meteorological model that utilized DLNMs to assess the risk from weekly, district-level, meteorological factors *(x*_*s*,*t*_*)* across lag periods. These included absolute and relative humidity, mean, minimum and maximum temperature, precipitation, wind speed, and the SPEI (a drought indicator). We evaluated model fit and effect size across 8 weeks of lag time from exposure.
4. To elucidate the risk from air pollution, we developed an air pollution model that included a DLNM cross-basis for PM_2.5_, PM_10_ and SO_2_ (aq_*s*,*t*_), including the same sociodemographic base model and meteorological confounding factors. We tested different formulations for inclusion of the meteorological confounding, including linear and nonlinear covariates of humidity and temperature with different lag times. We also evaluated the inclusion of a temperature cross-basis alongside the PM_2.5_ cross-basis, and temperature as a modifying interaction with the PM_2.5_ cross-basis. We found the WAIC to be similar across the temperature covariate model, the temperature cross-basis model and the interaction model. The PM_2.5_ effect sizes were also unchanged across these model types. The final air pollution model included a nonlinear lag 3 humidity covariate and linear lag 0 covariate for temperature.
5. Using our final air pollution model, we then focused on PM_2.5_ to determine how the risk from PM_2.5_ varies given different outcomes (for example, serotype, age group, disease type).
6. Finally, we incorporated a modification by different underlying genomic lineages with PM_2.5_ in the same model to elucidate how the underlying microbial population modifies these PM_2.5_ effects. The PM_2.5_ air pollution models all accounted for the potentially confounding effects of temperature, humidity, population density and vaccination.

#### Statistical analysis

All statistical analysis and visualizations were conducted using R (v.4.4.2)^73^ with key packages including R-INLA^74^, dlnm^75^, dplyr^76^ and sf^77^. A glossary of symbols and variables used is included in Supplementary Table 11.

##### Sociodemographic base model development

We began by building a model to account for residual spatiotemporal effects and important sociodemographic covariates such as population density, PCV or ART (treatment for HIV). We modelled weekly or monthly pneumococcal disease counts in South Africa between 2005 and 2023 or for 2005–2019 (excluding the post-COVID-19 period), by specifying a Bayesian hierarchical mixed model using pneumococcal disease case counts at the district (*n* = 52) or province (*n* = 9) level as the response variable. To account for overdispersion, we assumed that case counts, *y*_*s*_,_*t*_, where *s* represents a spatial unit and *t* represents time step (1–987 weeks, or 228 months), follow a negative binomial distribution such that:

1 $${y}_{s,t}|{\mu }_{s,t}\sim \mathrm{NegBin}({\mu }_{s,t},\kappa )$$

2 $$\mathrm{log}({\mu }_{s,t})=\mathrm{log}({\mathrm{pop}}_{s,a(t)})+\mathrm{log}({\rho }_{s,t})$$

where *μ*_*s*_,_*t*_ is the expected number of cases and *κ* is the overdispersion parameter. The distribution mean, *μ*_*s*_,_*t*_, is defined as the product of the population, pop_*s*,*a*(*t*)_, (per 100,000 people) in a given spatial unit (district or province), *s*, and year, *a*(*t*), with the disease incidence rate *ρ*_*s*,*t*_ estimated via a combination of covariates and random effects.

For all sociodemographic model formulations, we evaluated different spatial (district or province) and temporal (weekly or monthly) resolutions, ultimately only including the weekly district-level models in our main analysis (Supplementary Table 12).

##### Spatiotemporal random-effect assessment

We evaluated model performance by defining the response variable both at district and province level for weekly and monthly case counts and across various random and fixed-effect combinations, comparing them to the intercept-only model (Supplementary Table 12). These models included an intercept *α* as well as temporal random effects accounting for unknown and unmeasured monthly variation (calendar months January (1) to December (12)), *δ*_*m*__(__*t*__)_, and interannual effect *γ*_*a*__(__*t*__)_ at the national level. We tested both the monthly variation, *δ*_*m*(pr*(s*),*t*)_, and interannual effect, *γ*_pr(*s*),*a*(*t*)_, replicated by province pr(*s*) (*n* = 9 provinces). We also included spatial effects with a Besag–York–Mollié (BYM-2) model^78^, with both structured (*u*_*s*_) and unstructured effects (*v*_*s*_) at the district- and province-level models. In the province-level model, $s={\rm{pr}}(s)$. We additionally evaluated including the spatial random effects with a yearly replication to adjust for fine-scale spatiotemporal confounding (Supplementary Fig. 8).

##### Impact of pneumococcal vaccine and ART on interannual variation

We assessed different formulations of pneumococcal vaccination, antiretroviral therapy and population density. We tested inclusion of a single categorical covariate with three phases to account for decreasing case counts after the implementation of the PCV (PCV_periods_): pre-PCV7 (years before 2009), PCV7 (2009–2010 inclusive) and PCV13 (2011 onwards). We alternatively assessed models including only two periods for PCV (PCV_2009Vaccines_): pre-PCV (years before 2009, as above) and then a single post-PCV period (2010 onwards). We also evaluated models with national-level PCV coverage (%) per year (PCV_coverage,*a*(*t*)_), as well as a model accounting for two time periods for COVID-19 (covid): pre-covid (2005–2019) and post-covid (2020 onwards). To assess the impact of the uptake of ART across South Africa, we included a variable for ART coverage. These included a national-level ART coverage (%) (ART_coverage,*a*(*t*)_) and province-level ART coverage (%) (ART_coverage,pr(*s*),*a*(*t*)_). We tested inclusion of the intervention variables (PCV, ART, covid) independently and together, where *f* indicates a nonlinear function. We also included district-level human population density (pd) as a continuous variable due to its particular relevance as a confounding factor with air pollution.

In summary, the main models tested included: (1) PCV as a categorical variable with 2 categories, (2) PCV_2009Vaccines_ (M10), (3) PCV_periods_ (M11), (4) PCV_coverage,*a*(*t*)_ (M13), (5) ART_coverage,*a*(*t*)_ (M14), (6) ART_coverage,pr(*s*),*a*(*t*)_ (M15) and (7) ART_coverage,pr(*s*),*a*(*t*)_ and PCV_periods_ (M16) (Supplementary Table 13). M12 is the same as M11 with additional inclusion of population density. These were all run with and without replication of the interannual effect across provinces (Supplementary Table 3). We also ran different models for different types of IPD to assess the impact of these perturbations across different groups. These included children ≤5 years old, adults between 15 and 64 years old, adults 65 years old and above, IPD from only NVTs and from only VTs. We also ran these models including disease from 2005 to 2019 (excluding the post-COVID-19 period) and from 2005–2023 (Supplementary Table 3).

We found an increasing goodness-of-fit with increasing complexity (Supplementary Table 12). We included M12 as our sociodemographic base model due to the balance of complex random effects and goodness of fit. This model was formulated as equation (3):

3 $$\mathrm{log}({\rho }_{s,t})=\alpha +{\beta }_{\mathrm{pd}}{\mathrm{pd}}_{s,a(t)}+f({\mathrm{pcv}}_{\mathrm{periods}})+{\delta }_{m(t)}+{\gamma }_{\mathrm{pr}(s),a(t)}+{u}_{s}+{v}_{s}$$

##### Sociodemographic and spatiotemporal effect assessment

Our sociodemographic base model, which included spatial autocorrelation across districts, seasonal effects and interannual random effects, was replicated across provinces, as well as pneumococcal vaccine implementation periods and population density (Supplementary Table 13). We found similar effects for models spanning 2005–2023 and 2005–2019 but due to the dramatic decrease in IPD cases during the COVID-19 pandemic period (2020 onwards), we proceeded with models restricted to the 2005–2019 period throughout (Supplementary Tables 12 and 14). These models were fitted to weekly invasive disease incidence across South Africa district levels (*n* = 52) (Supplementary Fig. 1a,b). The Gauteng and Western Cape provinces had a higher risk of IPD than the national average. The RR derived from the spatial effect for Gauteng province was 13.35 (95% CI 4.19–44.34) and 1.54 (95% CI 0.84–2.70) for Western Cape, representing the area-specific risk relative to the average across all provinces. The risk in Western Cape was largely driven by the increased risk in the Cape Town district alone (relative to all-district average) (RR 10.16: 95% CI 7.89–13.09). All districts in Gauteng province exhibited elevated disease risk relative to the national average (RR > 1). Across the other provinces, districts that host major cities and transport hubs had twice the risk of IPD compared to the average. Some of the exacerbated risk in the main cities, such as those around Johannesburg in Gauteng, may reflect travel to hospitals from more rural areas (Extended Data Fig. 1a, and Supplementary Fig. 1c and Table 14). There was a seasonal effect with an increased disease risk between the months of May and October, with peak risk in August (RR 1.37: 95% CI 1.33–1.41). In the interannual effect, we found a reduction in risk over time even while accounting for vaccination for Gauteng, Free State, Mpumalanga, KwaZulu-Natal and North West provinces (Extended Data Fig. 1c and Supplementary Table 14). This may be attributed to ART uptake also being influential there (Supplementary Fig. 1d).

In developing the sociodemographic base model, we examined the impact of ART and vaccination coverage. They were correlated (Pearson *R*^*2*^ = 0.84) and model formulations that included them had reduced interannual effect variance (Supplementary Fig. 1d and Extended Data Fig. 2a). We found no effect of ART or PCV on NVT serotypes alone in the 2005–2019 period. A lack of PCV effect on NVTs is expected, as the vaccine does not protect against these serotypes. In contrast, ART is expected to confer protection against all pneumococcal serotypes by improving immune function in PLHIV, particularly among adults, who represent the group most likely to be receiving ART in this high HIV-burden setting^79,80^. In the 2005–2019 period, NVT IPD cases were skewed towards children and older adults, but extending the analysis to 2020–2023 allowed an ART effect on NVTs to emerge. In this time period, there was a higher number of IPD cases among adults aged 18–64 years who, in this setting, are the primary recipients of ART. This increase in adult cases might have resulted from the resurgence of IPD after the COVID-19 pandemic and a gradual decline in indirect vaccine protection over time (Extended Data Fig. 2c–e)^81–83^. Overall, the impact of ART on opportunistic infections cannot be understated^26,79,80,84^. Due to the nuanced impact of ART, and the established direct relationship between pneumococcal vaccination and IPD, we included the three PCV periods (pre-PCV7, post-PCV7 and post-PCV13) as the intervention covariate in the final sociodemographic base model (equation (3)) (Extended Data Fig. 1d and Supplementary Table 3).

#### DLNMs to estimate disease risk with environmental factors

We estimated IPD risk using DLNMs built on the sociodemographic base model described in equation (3). Correlations between environmental variables were assessed with Pearson correlation, while the relationship between exposure lag times and disease was explored using Spearman correlation (Extended Data Fig. 3). We tested all models including both the province of Gauteng alone and excluding Gauteng province, due to it being the most populous and having the highest incidence of IPD and highest maximum concentrations of PM_2.5_, PM_10_ and SO_2_ (Supplementary Figs. 4–6). We ran weekly models at both province (adm1) and district (adm2) levels. Sensitivity analyses across 6-, 8- and 12-week windows for all variables and administrative regions revealed little variation across the cumulative-effects estimates; the marginal lag-response plots (Supplementary Figs. 15 and 16) demonstrated that associations typically stabilized towards null with an 8-week lag time from exposure. Previous literature identified environmental IPD influences up to 4 weeks after environmental exposure^16,17,19^, and our cross-correlation analyses indicated maximum association extending to a 5-week lag (Extended Data Fig. 3). Given this statistical evidence together with the estimated 30-day generation time for pneumococci, we selected an 8-week lag window as a robust time frame to capture both the statistically and biologically relevant effects^8^. While we evaluated many models across different spatial and temporal scales, our final meteorological and air pollution models presented in the main text were at the weekly district level from 2005 to 2019 (Supplementary Table 4).

##### Meteorological and air pollution model DLNM specifications

We estimated the exposure–lag-response association using the crosspred() function of the dlnm package for all DLNMs to generate estimates and Gaussian-approximate credible intervals derived from the INLA posterior confidence intervals. Although crosspred() typically reports confidence intervals, in this case the uncertainty reflects the Bayesian posterior distribution because the supplied coefficients and covariance matrix were obtained from INLA. To model the nonlinear and delayed effects of environmental factors, we employed natural cubic splines for both the exposure–response (argvar) and lag-response (arglag) functions. We conducted sensitivity analyses to determine the optimal degrees of freedom (d.f.) for these splines (Supplementary Fig. 17 and Table 15). Some of these specification adjustments induced numerical instability in the exposure–response curves. Specifically, for absolute humidity, and maximum and minimum temperature, the hyper-flexible argvar specification produced spurious oscillations. These oscillations did not have any epidemiologically plausible explanation and probably reflected overfitting rather than meaningful structure. We ultimately included the more-constrained nonlinear specification which preserved overall shape while avoiding artificial fluctuations. The final DLNM cross-basis used a natural cubic spline for the lag response with 2 d.f. (covering an 8-week lag period), except for relative humidity for which 3 d.f. was used to better capture potential nonlinear lag structures^85,86^. For each exposure–response function, we utilized a natural cubic spline with 3 d.f., placing internal knots at the 33rd and 67th percentiles. For precipitation, two knots were placed at equally spaced intervals on the log-transformed distribution to account for its right skew. These specifications were consistent with established DLNM approaches and maintained model parsimony with sufficient flexibility to capture delayed and nonlinear exposure–response associations without overfitting^87,88^ (Supplementary Table 4).

##### Meteorological models

We first developed our meteorological model to assess the effect of meteorological factors on pneumococcal disease dynamics. We added DLNMs for all meteorological factors to the sociodemographic base model (equation (4)). These included the weekly average of the daily temperature mean (tas), minimum (tasmin) and maximum (tasmax) temperature, absolute (absh) and relative humidity (hurs), the weekly precipitation mean (prlrmean) and sum (prlrsum), the SPEI (drought indicator) for 3 months (SPEI3) and 6 months (SPEI6), as well as weekly mean of the daily average windspeed (sfcWind)^75,89^. We centred and scaled all meteorological factors. We centred the effect estimations at the median of each variable’s distribution, which provided a neutral benchmark to account for a meteorological baseline. Including a centre at the median is recognized when a biological optimal exposure point is not defined for a health outcome, as is the case for IPD^75^.

4 $$\begin{array}{l}\log ({{{\rho }}}_{{{s}},{{t}}})={\rm{\alpha }}+{{{\beta }}}_{{\rm{pd}}}{{\rm{pd}}}_{{{s}},{{a}}({\rm{t}})}+{\rm{f}}\left({{\rm{pcv}}}_{{\rm{periods}}}\right)+{{\rm{CB}}}_{x}({x}_{{{s}},{{t}}-{\ell}})\\\qquad\qquad+\,{{\rm{\delta }}}_{{{m}}({{t}})}+{{\rm{\gamma }}}_{{\rm{pr}}({{s}}),{{a}}({{t}})}+{{{u}}}_{{{s}}}+{{{v}}}_{{{s}}}\end{array}$$

##### Air pollution models

We developed an air pollution model to assess the risk from air pollution on IPD. We included the same sociodemographic base model as above, including vaccination period and population density covariates as well as residual spatiotemporal effects, and additionally tested different formulations of confounding meteorological effects. Meteorological factors are known confounders of air pollution and their inclusion also ensures that our model is applicable across varied climates. We developed our meteorological covariates given the known interactions between temperature and air pollution, and humidity and temperature^13,17,86^. Our final air pollution model included mean temperature as a linear term (lag 0) and absolute humidity as a nonlinear term (lag 3), implemented at a random walk of order 2 (equation (5)). We chose this formulation given the effect sizes of mean temperature and absolute humidity at these lag times and after comparison against other model formulations for the different confounders (described below) (Supplementary Fig. 3 and Table 4). For all air pollution models, we included 3 d.f. for the lag-response spline and equally distributed knots at the 33rd and 67th percentile for the exposure–response spline (Supplementary Figs. 16 and 17).

We assessed air pollution models with the confounding effects of mean or maximum temperature alone, as well as with the additional effects of absolute or relative humidity. We formulated the humidity and temperature models including both variables at no-lag time, a 3-week lag time, or a mixture of one at 3 weeks and another at 0. We included the humidity variables as nonlinear effects using random walk 2 models with 5 cuts and included the temperature variables as linear effects given their exposure–response curves from the meteorological models (Fig. 3 and Extended Data Fig. 5). We also assessed directly interacting a PM_2.5_ cross-basis with a mean temperature covariate, as well as including an additional mean temperature cross-basis together with an absolute humidity covariate, finding similar goodness of fit and risk estimates across all different formulations including both humidity and temperature (ΔWAIC < 4) (Supplementary Tables 4 and 7). For parsimony, and given the goodness-of-fit metrics, we proceeded with the covariate model including lag-week 3 absolute humidity and mean temperature covariates with no lag-time (equation (5)).

5 $$\begin{array}{l}{\mathrm{log}}({\rho }_{s,t})={\rm\alpha }+{{\beta }}_{\mathrm{pd}}{\mathrm{pd}}_{s,a(t)}+f({\mathrm{pcv}}_{\mathrm{periods}})+{\beta }_{\mathrm{temp}}{\mathrm{temp}}_{s,t}+f({\mathrm{hum}}_{s,t-3})\\\qquad\qquad+\,{\mathrm{CB}}_{\mathrm{aq}}({\mathrm{aq}}_{s,t-{\mathcal{l}}})+{\delta }_{m(t)}+{\gamma }_{\mathrm{pr}(s),a(t)}+{u}_{s}+{v}_{s}\end{array}$$

We centred the air quality metrics at the 24-h NAAQS for South Africa (PM_2.5_ = 40 μg m^−3^, PM_10_ = 75 μg m^−3^ and SO_2_ = 125 μg m^−3^). This contrasts with the 24-hour WHO standards set in 2021 (PM_2.5_ = 15 μg m^−3^, PM_10_ = 45 μg m^−3^ and SO_2_ = 40 μg m^−3^). This centering strategy was chosen to align our risk estimates with national regulatory frameworks, providing a direct assessment of health risks associated with standard exceedance rather than an arbitrary minimum value. We utilized the 24-hour exceedance threshold rather than the annual threshold as we are examining an acute IPD effect (Supplementary Tables 4 and 16).

##### PM_2.5_ effect across age, disease and vaccine status

To identify differences in risk from PM_2.5_ across different stratification groups, we used our air pollution model formulation but changed the outcome from IPD overall to disease incidence per 100,000 people among each group for age group, disease outcome, vaccine status (whether cases were serotypes that were included in the PCV7 vaccine, PCV13 vaccine, or were non-vaccine type) and by specific serotype. The specific models we tested included (1) age ≤5 years old, (2) age 6–14 years old, (3) age 15–64 years old, (4) age ≥65, (5) bacteraemia, (6) meningitis, (7) other invasive disease types and (8) penicillin-resistant cases (measured at the meningitis threshold). We also included serotype stratifications by (9) NVT, (10) PCV7 and (11) PCV13. Models 12–29 included stratification by IPD from particular serotypes (Supplementary Tables 2 and 4). To estimate the relative risk ratio for each subgroup (serotype, disease type, age group) compared to the overall population, we crosscut the relative risk from the exposure–response curve at 50 μg m^−3^ concentration for each. We then included the relative risk for each subgroup in turn as the numerator compared to the relative risk from the overall model to estimate the increased (>1) or decreased (<1) risk of IPD in each subgroup, from PM_2.5_ compared to the risk for pneumococcal disease overall (Fig. 3a).

#### DLNMs interacting with lineage prevalence

To assess the impact of underlying lineage diversity on the environmental effects, we utilized the same air pollution model framework as above (comprising the sociodemographic base model (M12): seasonal, spatial and province-replicated interannual random effects, together with vaccination periods and population density, and adjustment for temperature and humidity) (Supplementary Table 13). We included all GPSCs with *N* ≥ 100 as well as GPSC8 due to its epidemiological importance in South Africa^8,51^. We then included an interaction between the exposure cross-basis and a modifying variable representing GPSC-specific proportions per total number sequenced each week of dominant GPSCs. We ran these models at the province level weekly and thus included a province-level spatial effect for these interaction models. To ensure model interpretability, we scaled and centred this variable (Supplementary Table 10).

We evaluated the exposure–response relationship at three representative values of the effect modifier, corresponding to the 0.1, 0.5 and 0.8 quantiles of the centred distribution of the modifying variable (excluding zeros). For each quantile value *z*, we derived level-specific coefficient vectors and corresponding variance–covariance matrices that reflect the combined main and interaction effects.

To account for uncertainty in the interaction effects across different levels of the modifying variable, we computed level-specific coefficient vectors and their corresponding variance–covariance matrices. For a given value *z* of the centred interaction variable, the coefficient vector was calculated as:

6 $${\beta }_{z}={\beta }_{0}+z {\beta }_{\mathrm{int}}$$

and the associated variance–covariance matrix as:

7 $${V}_{z}={V}_{0}+{z}^{2} {V}_{\mathrm{int}}+2z {V}_{\mathrm{cross}}$$

where ${\beta }_{0}$ and ${V}_{0}$ are the estimated coefficients and variance–covariance matrix for the main (non-interaction) effect, ${\beta }_{\mathrm{int}}$ and ${V}_{\mathrm{int}}$ are those for the interaction term, and ${V}_{{cross}}$ represents the covariance between main and interaction coefficients. These were then passed to the crosspred() function of the DLNM package to generate estimates and Gaussian-approximate credible intervals derived from the INLA posterior at each value of z (Supplementary Table 10).

We performed a sensitivity analysis whereby for GPSC pairs, which share the same dominant serotypes, we split the dataset into the pre- and post-vaccine period to ensure that the effect was not just driven by serotype replacement in the time series but by some underlying evolutionary process (Supplementary Fig. 14).

To estimate goodness of fit for each model, we calculated the WAIC^90^ and the mean absolute error. We also calculated the sum of the log_10_ conditional predictive ordinate for each observation to evaluate the model’s out-of-sample predictive performance. We additionally computed a pseudo-*R*^2^, defined as the proportionate reduction in deviance compared to an intercept-only model^91,92^. This measured the ability of each model to account for variation in disease incidence (0–1) whereby a model with *R*^2^ = 1 would indicate a model accounting for all of the variation in the data. This helped evaluate the improvement in model fit achieved by including additional covariates.

### Reporting summary

Further information on research design is available in the Nature Portfolio Reporting Summary linked to this article.

## Supplementary information

Supplementary Information (Figures) Supplementary Figs. 1–17.

Reporting Summary

Peer Review File

Supplementary Tables 1–17 Supplementary Tables 1–17.

## Source data

Source Data Fig. 1 Fig. 1a–d source data: incidence, hospital locations, summarized sociodemographic and environmental data across time. Sheets labelled Fig. 1a, Fig. 1b, Fig. 1c and Fig. 1d.

Source Data Fig. 2 Fig. 2a–c source data: cumulative fits for final models. Sheets labelled Fig. 2a, Fig. 2b1, Fig. 2b2, Fig. 2c1, Fig. 2c2 and Fig. 2c3.

Source Data Fig. 3 Fig. 3a–d source data: relative risk ratios, cumulative fits for age group and disease type, and lag fits for vaccine types. Sheets labelled Fig. 3a, Fig. 3b, Fig. 3c and Fig. 3d.

Source Data Fig. 4 Fig. 4a–c source data: proportions by year and province for GPSC21, proportion of GPSC1 and GPSC21 of serotype 19F, fits for GPSC1 and GPSC21 over 8 weeks of lags. Sheets labelled Fig. 4a, Fig. 4b and Fig. 4c.

Source Data Extended Data Fig. 1 Extended Data Fig. 1a–e source data: spatial effects medians, seasonal effect medians and CIs, interannual effects, fixed-effects vaccine periods and population density effect. Sheets labelled ED Fig. 1a, ED Fig. 1b, ED Fig. 1c and ED Fig. 1d,e.

Source Data Extended Data Fig. 2 Extended Data Fig. 2a–e source data: fits for models with different perturbations, including the interannual effects and the fixed effects for each, case count by vaccine type by age over time. Sheets labelled ED Fig. 2a, ED Fig. 2b, ED Fig. 2c, ED Fig. 2d and ED Fig.2e.

Source Data Extended Data Fig. 3 Extended Fig. 3 source data: Spearman correlations across lags for each environmental variable by province. Sheet labelled ED Fig. 3.

Source Data Extended Data Fig. 4 Extended Data Fig. 4a–e source data: Pearson correlations between all variables, seasonal relative humidity over time, maximum temperature and relative humidity relationship, environmental variables over time by province, and mean spatial value for environmental variables. Sheets labelled ED Fig. 4a, ED Fig. 4b, ED Fig. 4c, ED Fig. 4d and ED Fig. 4e.

Source Data Extended Data Fig. 5 Extended Data Fig. 5a–f source data: fits with weekly lags for environmental variables in final models. Sheets labelled ED Fig. 5a, ED Fig. 5b, ED Fig. 5c, ED Fig. 5d, ED Fig. 5e and ED Fig. 5f.

Source Data Extended Data Fig. 6 Extended Data Fig. 6a–d source data: cumulative fits for non-main model environmental variables precipitation, drought, wind and ozone. Sheets labelled ED Fig. 6a1, ED Fig. 6a2, ED Fig. 6b, ED Fig. 6c and ED Fig. 6d.

Source Data Extended Data Fig. 7 Extended Data Fig. 7a,b source data: number of weeks per province with PM2.5 ≥ South African PM2.5 threshold and frequency of PM2.5 concentrations by province overall. Sheets labelled ED Fig. 7a and ED Fig. 7b.

Source Data Extended Data Fig. 8 Extended Data Fig. 8a–d source data: weekly and cumulative fits for each serotype model of air pollution (PM2.5). Sheets labelled ED Fig. 8a–c and ED Fig. 8d.

Source Data Extended Data Fig. 9 Extended Data Fig. 9 source data: extracted from the crosspred object for the surface plots of PM2.5 risk over time modified by each GPSC. Sheet labelled ED Fig. 9.

Source Data Extended Data Fig. 10 Extended Data Fig. 10a,b source data: location of observations of air quality and air quality concentrations in 3 provinces and 1 district across 4 models and observations over time. Sheets labelled ED Fig. 10a, ED Fig. 10b_Gaut, ED Fig. 10b_GS, ED Fig. 10b_KZN and ED Fig. 10b_WC.

## Data Availability

All datasets and models are available on Zenodo (https://doi.org/10.5281/zenodo.17877255)^93^. Genome accession numbers are in Supplementary Table 17. Source data are provided with this paper.

## Code availability

All code is available in GitHub (https://github.com/sophbel/env_sa_manuscript)^94^.
