# Supplementary material for: Pneumococcal population structure influences the effects of air pollution on invasive disease risk in South Africa
Source: Nat Microbiol. 2026 Sep 14;11(10):2723–37. doi: 10.1038/s41564-026-02458-5 (PMC13716828; doi:10.1038/s41564-026-02458-5)
Supplement: Supplementary file 1 [file 41564_2026_2458_MOESM1_ESM.pdf]

nature microbiology

Supplementary information https://doi.org/10.1038/s41564-026-02458-5

Pneumococcal population structure influences the effects of air pollution on invasive disease risk in South Africa

In the format provided by the authors and unedited

Supplementary Figures

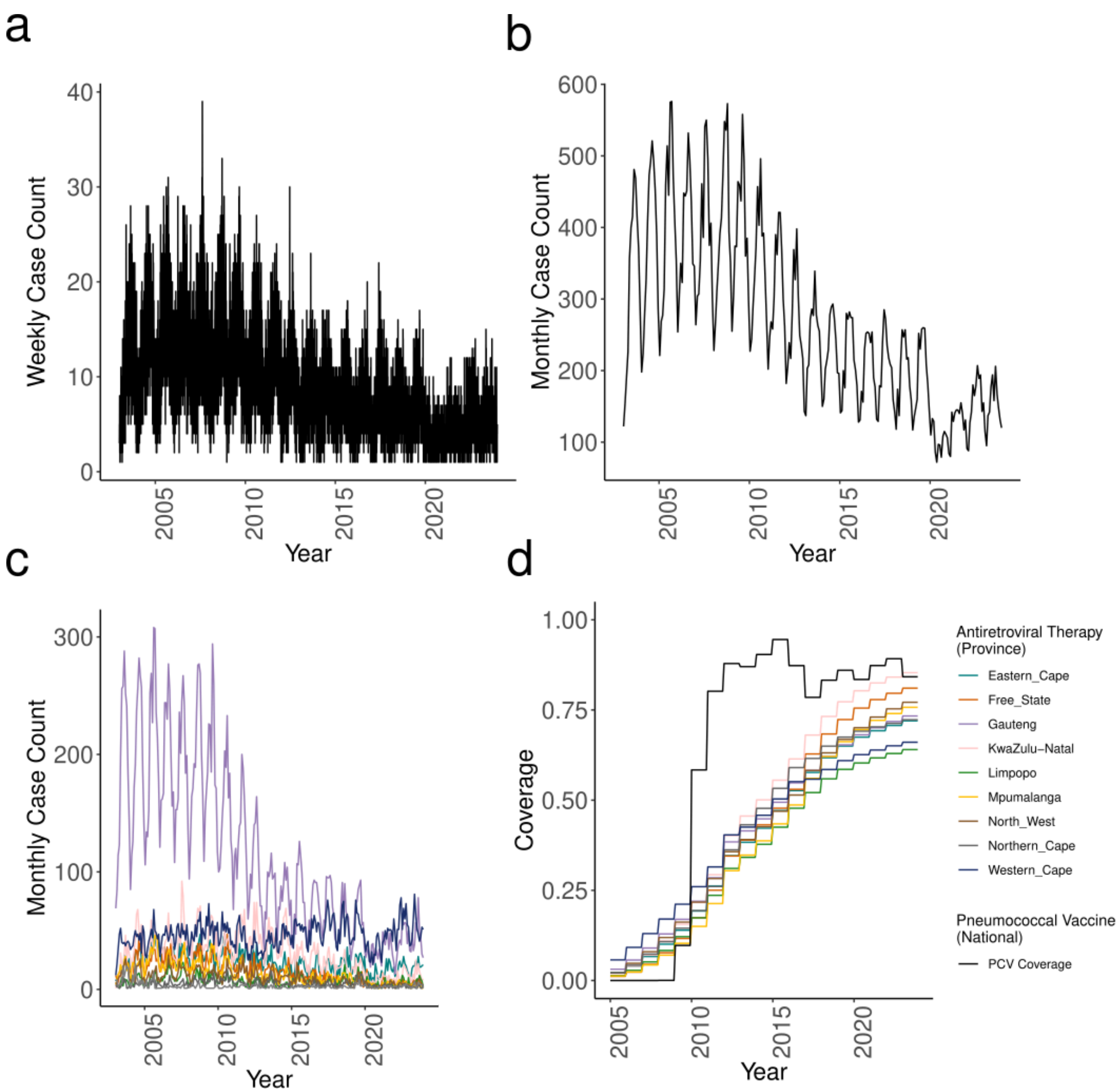

Figure S1. Pneumococcal case counts (a-c) and intervention coverage (d) (a) Weekly (a) and Monthly (b) pneumococcal disease case counts. (b) Disease counts monthly colored by province. (c) pneumococcal conjugate vaccine (PCV) coverage nationally and antiretroviral therapy coverage by province over time.

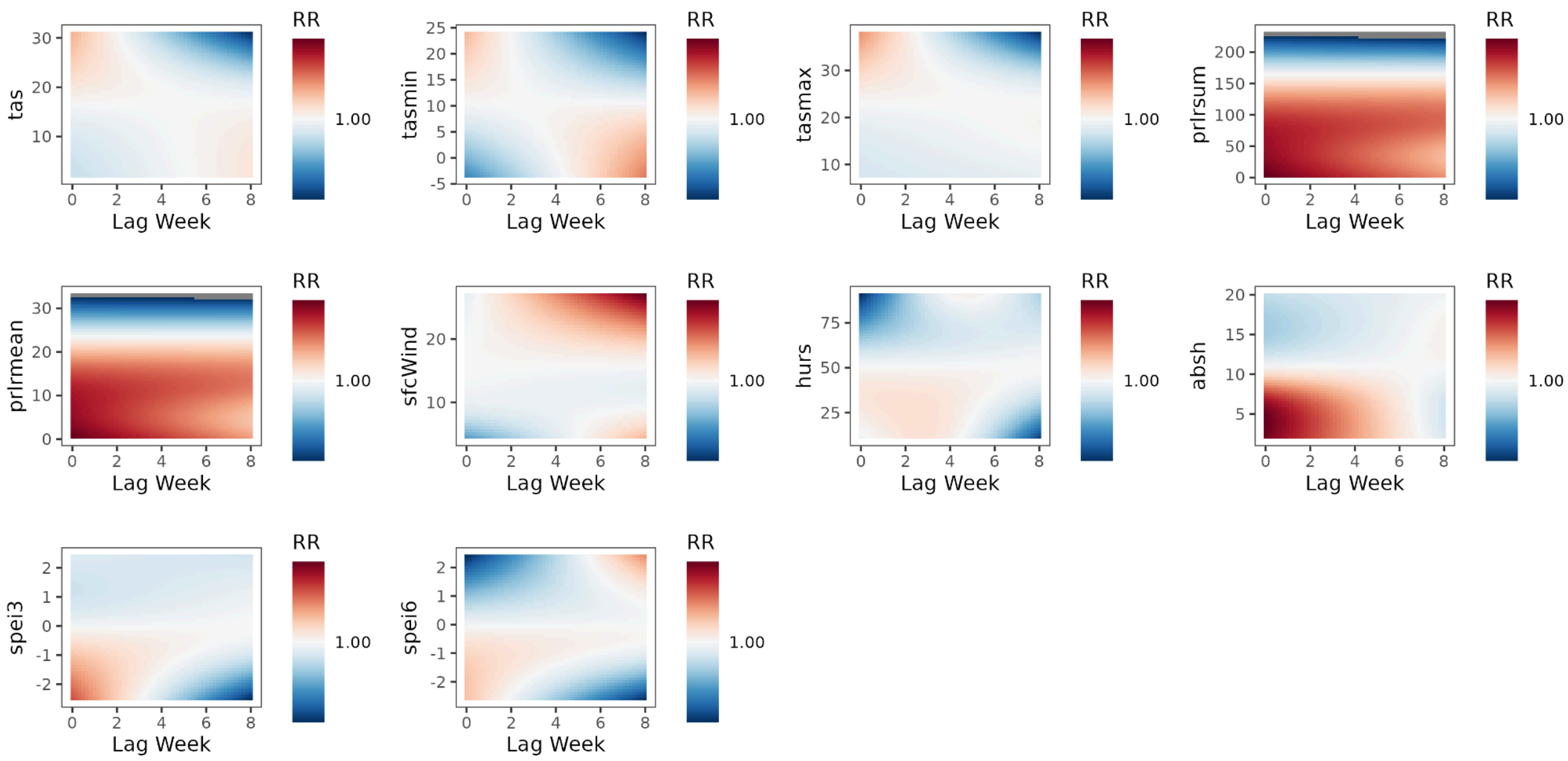

Figure S2. Mean relative risk (RR) surfaces from meteorological models where mean risk >1 is red and <1 is blue for temperature (celsius; mean = tas, maximum = tasmax, minimum = tasmin), precipitation (millimeters; sum = prlrsum, mean = prlrmean), wind speed (km/hour; sfcWind), relative humidity (% hurs), absolute humidity (g/m^3; absh), standard precipitation evapotranspiration index (3 months = spei3, 6 months = spei6).

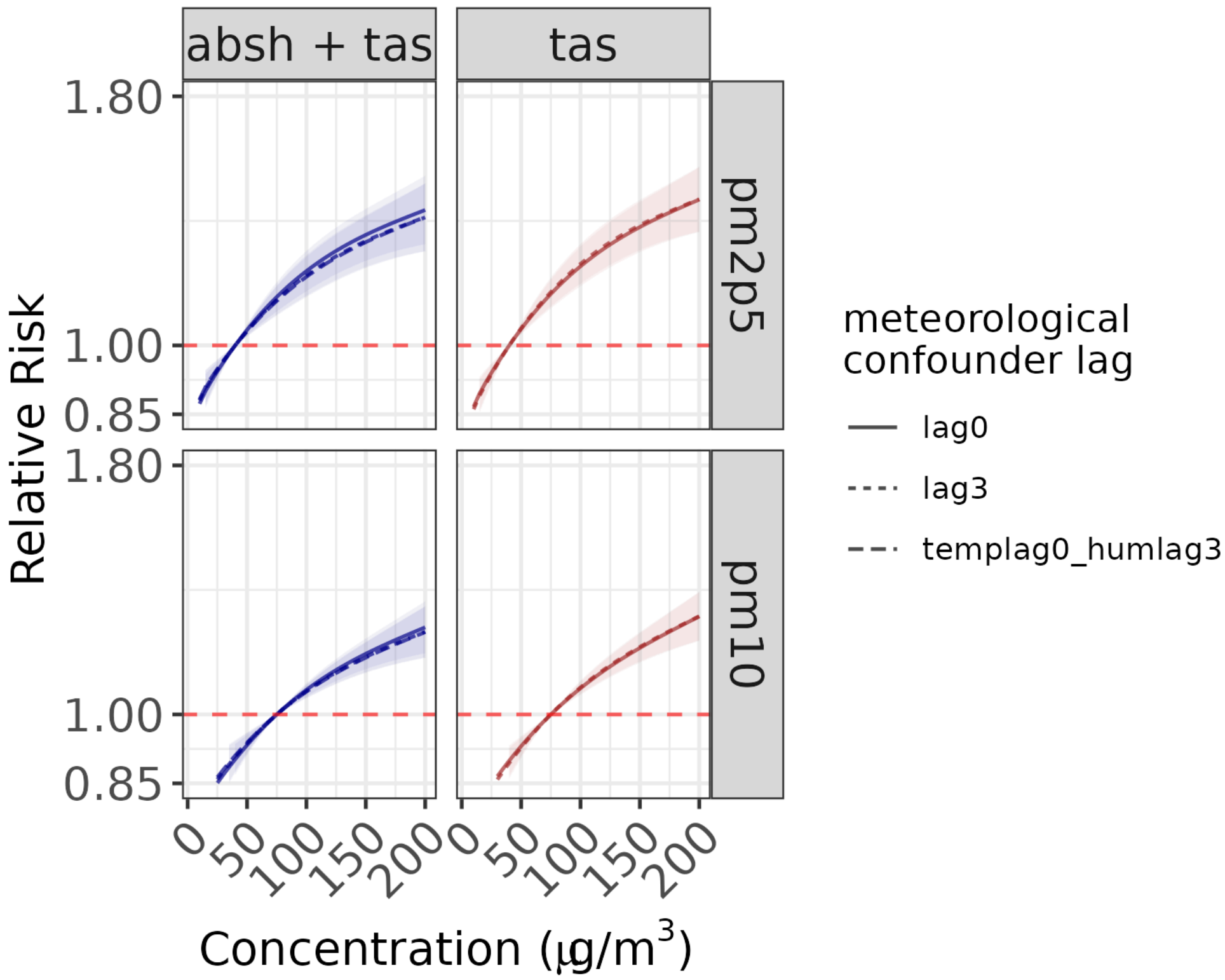

Figure S3. Exposure-response curves across 8 weeks of lags for the relative risk (y-axis) from each $PM_{2.5}$ (top) and PM_{10} (bottom) (x-axis) in DLNM models. These models are fit to weekly district level case data with the sociodemographic base model and $PM_{2.5}$ or PM_{10} DLNMs with additional absolute humidity (absh) and mean temperature (tas) incorporating meteorological confounding covariates at different lag times. Lag times incorporated here include mean temperature with no lag time (solid) and a 3-week lag (dotted) time and absolute humidity with temperature both with no lag time, both at a 3-week lag time, and with humidity at a 3-week lag time and mean temperature with no lag time (dashed). The best model formulation is the one with the mixture of humidity at 3 weeks and temperature with no lag. The shaded error bars represent 95% credible intervals and the points indicate the median.

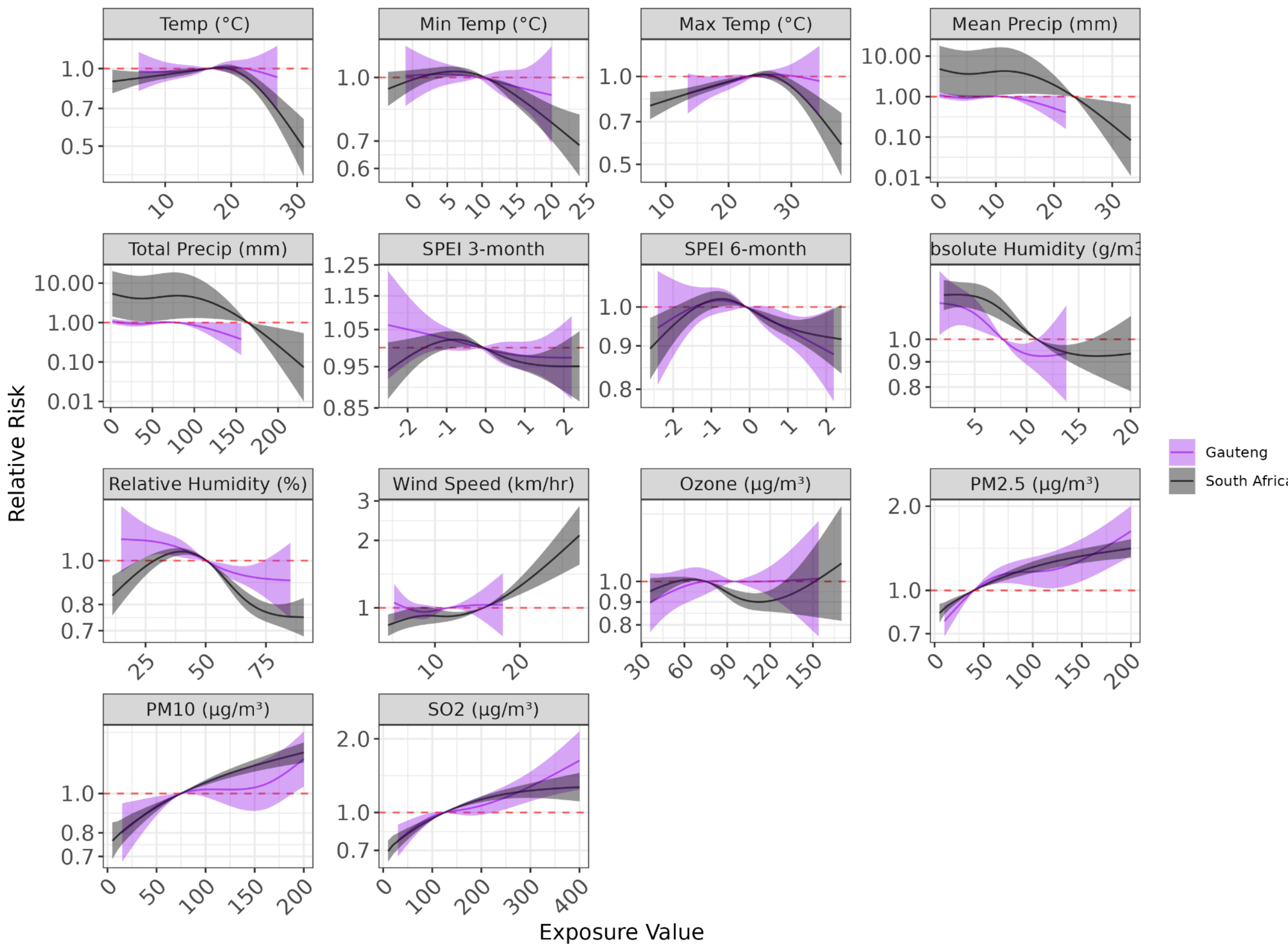

Figure S4. Cumulative exposure-response curves across 8 weeks of lags for the relative risk (y-axis) from each environmental variable (x-axis) in DLNM models for Gauteng alone (purple) and South Africa overall (black). These models are fit to weekly district level case data including the sociodemographic base model and distributed lag non-linear models (DLNMs) for each variable. Included variables in order are temperature (mean, minimum, and maximum celsius), precipitation (weekly mean and sum total millimeters (mm)), SPEI 3-month and 6-month, Humidity (absolute and relative), wind speed (km/hr), and ozone. For $PM_{2.5,}$ PM_{10}, and SO_2 confounding covariates mean temperature and absolute humidity are included. (a) absolute humidity (absh) in g/m^3 water (b) percent relative humidity (c) ozone (o3) (d) particulate matter <10µm (pm10) (e) particulate matter <2.5µm (pm2p5) (f) mean precipitation millimeters (g) cumulative precipitation in millimeters (h) mean wind speed in km/hour (i) sulfur dioxide (so2) (j) drought index including evapotranspiration across previous 3 months (k) drought index including evapotranspiration across previous 6 months (l) maximum temperature (celsius) (m) minimum temperature (celsius). The shaded error bars represent 95% credible intervals and the points indicate the median.

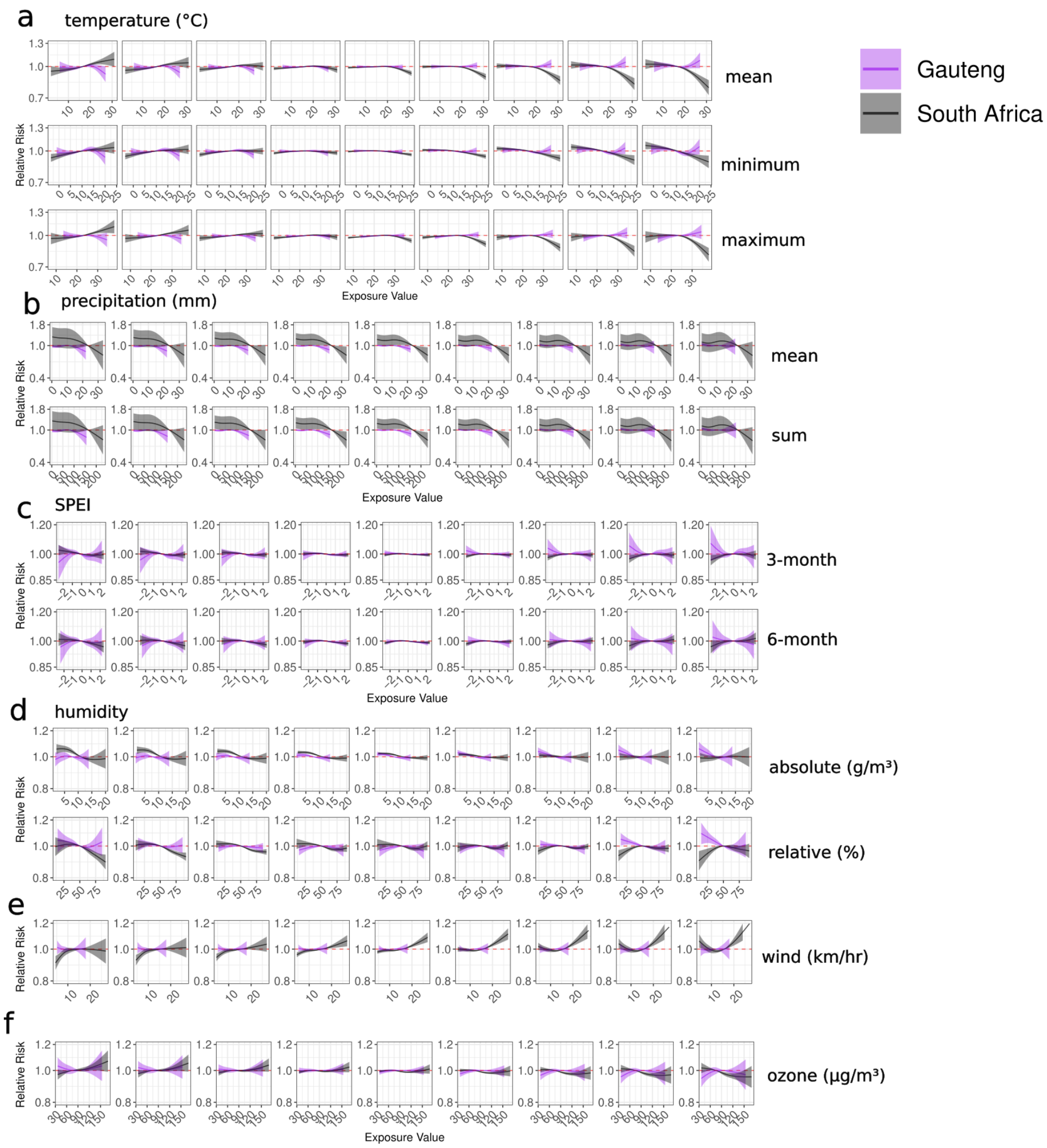

Figure S5. Exposure-response curves across 8 weeks of lags for the relative risk (y-axis) from each environmental variable (x-axis) in DLNM models for Gauteng alone (purple) and South Africa overall (black). These models are fit to weekly district level case data with the sociodemographic base model and additional environmental variable DLNMs. The metric for each exposure value is on the x-axis and the relative risk is on the y-axis. (a) mean (top), maximum (middle), and minimum (bottom) temperature in celsius. (b) millimeters precipitation mean (top) and sum (bottom) (millimeters; mm). (c) Standard precipitation evapotranspiration index (SPEI) for 3-months (top) and wind (kilometers per hour; km/hr), and (f) concentration of ozone (μg/m^3). The shaded error bars represent 95% credible intervals and the points indicate the median.

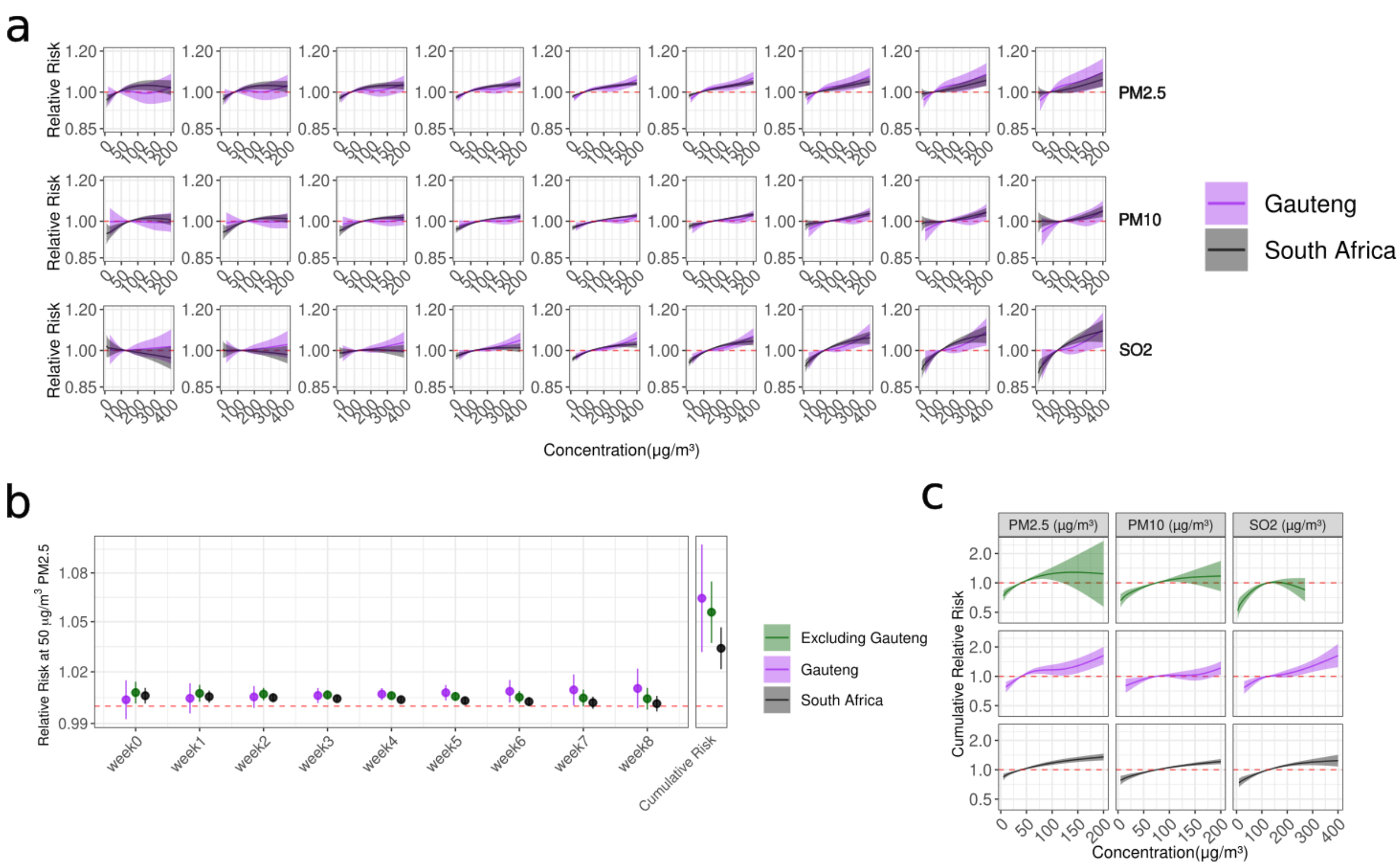

Figure S6. Comparison of models run on all of South Africa (black), Gauteng alone (purple) and for b-c excluding Gauteng (green). These models are fit to weekly district level case data with air pollution model (including the sociodemographic base model, temperature and humidity confounders and the DLNM crossbasis for each pollutant)**.** (a) exposure-response curves across 8 weeks of lags for relative risk from air pollutant exposure. for Gauteng alone (purple) and South Africa overall (black) for $PM_{2.5}$ (top), PM_{10} (middle), and SO_2 (bottom). (b) Relative risk at 50μg/m^3 of $PM_{2.5}$ for each of the lag weeks from 0-8 and the cumulative risk. (c) The cumulative relative risk for each of $PM_{2.5}$ (left), PM_{10} (middle), and SO_2 (right) excluding Gauteng (green), Gauteng alone (purple), and for South Africa overall (black). The shaded error bars represent 95% credible intervals and the points indicate the median.

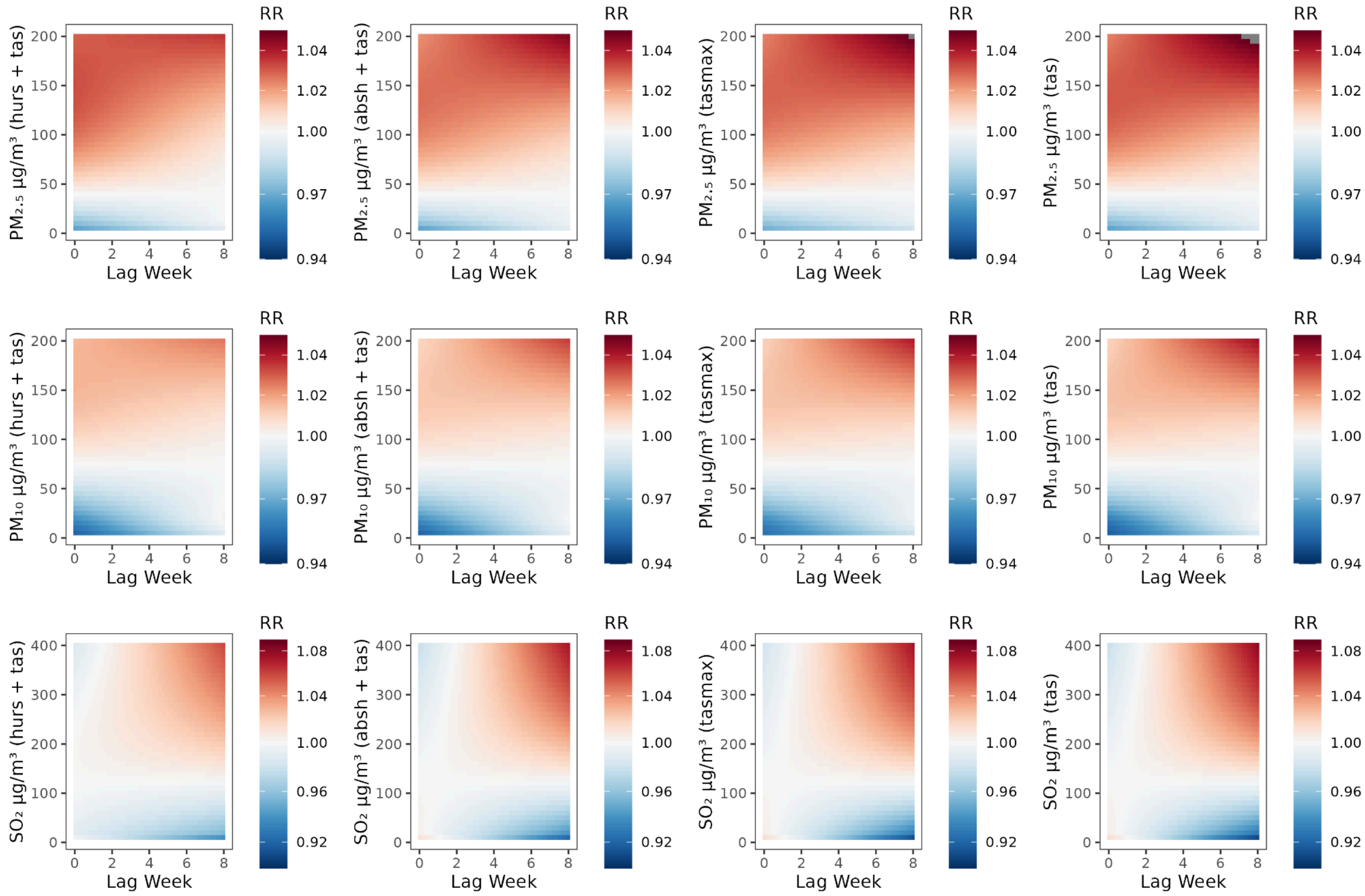

Figure S7. Median relative risk (RR) surfaces from the air pollution models where risk >1 is red and <1 is blue for $PM_{2.5}$ (top row), PM_{10} (middle row), and SO_2 (bottom row) concentration μg/m^3 including adjustment for different formulations of meteorological confounders from left to right for (i) relative humidity and temperature, (ii) absolute humidity and temperature, (iii) maximum temperature, and (iv) mean temperature. The humidity variables are included as non-linear at lag week 3 and the temperature variables are linear at lag week 0. The main air pollution model includes an adjustment for absolute humidity and mean temperature.

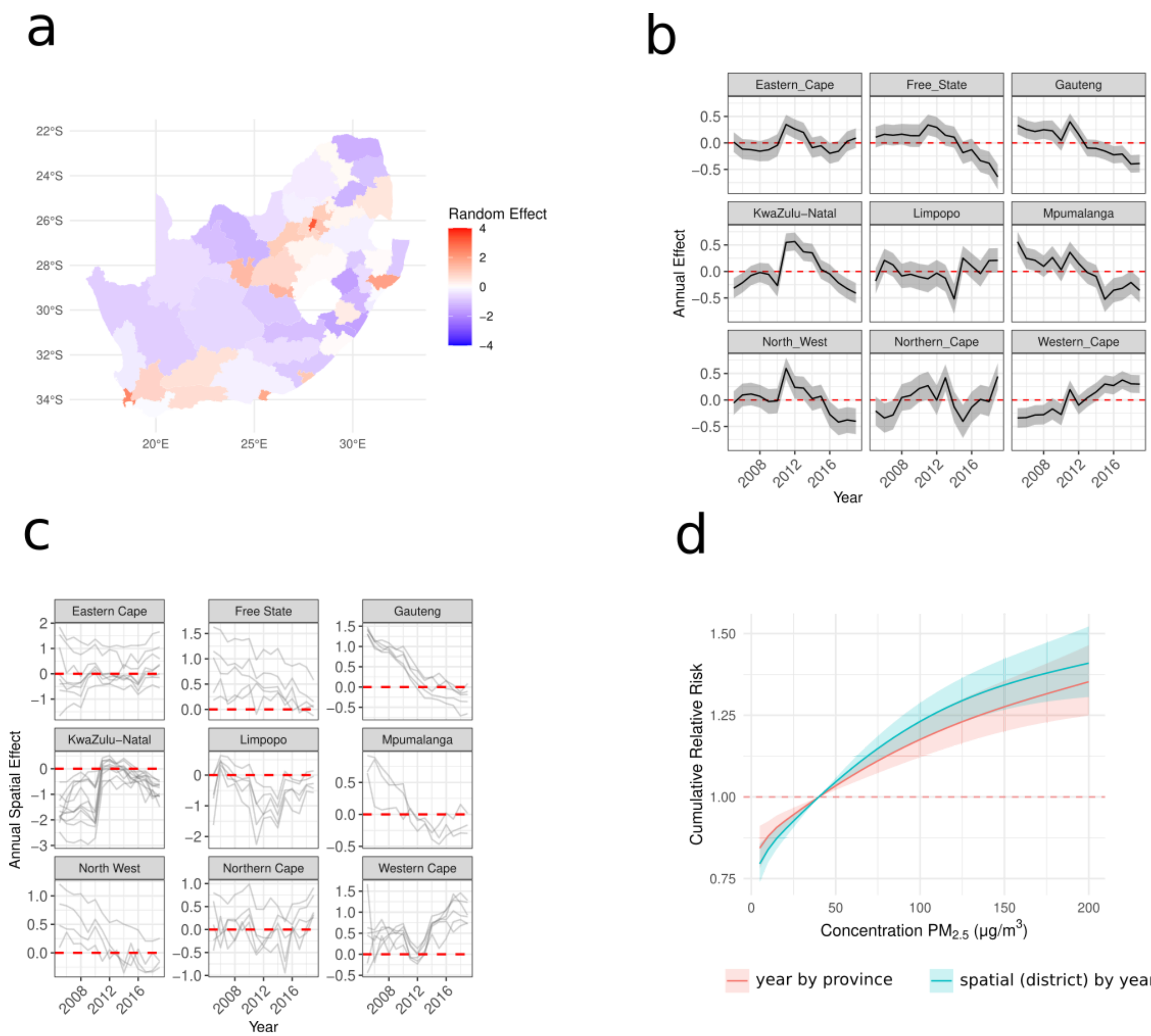

Figure S8. Model sensitivity with time varying spatial effects. (a) static spatial random effect from equation 1. (b) interannual effect replicated by province from equation 1. (c) spatial effect replicated by year for each district from equation 2. (d) Cumulative exposure-response curve across 8-weeks with the relative risk (y-axis) across weekly μg/m^3 of $PM_{2.5}$ (x-axis) comparing the air pollutant model from the main text (pink) ($\log(\rho_{s,t}) = \alpha + \beta_{pd}pd_{s,a(t)} + f(pcv_{periods}) + \beta_{temp}temp_{s,t} + f(hum_{s,t\text{-}3}) + CB_{aq}(aq_{s,t-\ell}) + \delta_{m(t)} + \gamma_{pr(s),a(t)} + u_s + v_s$) to a model which varies the spatial variation by year (blue) ($\log(\rho_{s,t}) = \alpha + \beta_{pd}pd_{s,a(t)} + f(pcv_{periods}) + \beta_{temp}temp_{s,t} + f(hum_{s,t\text{-}3}) + CB_{aq}(aq_{s,t-\ell}) + \delta_{m(t)} + u_{s,a(t)} + v_{s,a(t)}$).

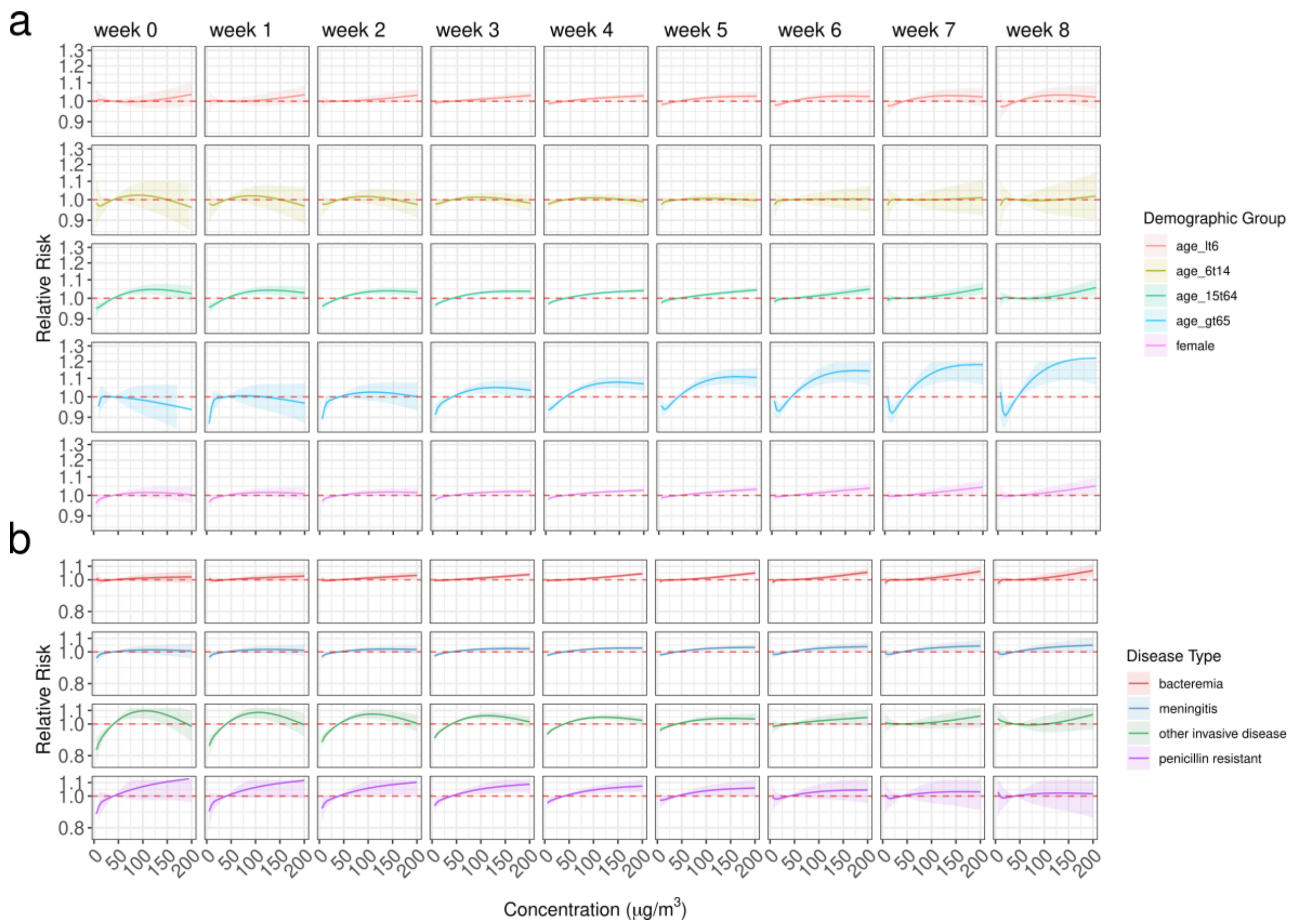

Figure S9. Exposure-response curves across different weekly exposure to $PM_{2.5}$ (concentration μg/m^3) including different stratifications of invasive disease including (a) demographic factors such as age <6 (red), age 15-64 (green), age >64 (blue), and females alone (purple) and for (b) different disease types including bacteremia (red), meningitis (blue), and other invasive disease types (dark green) all across 8 weeks of lags at the weekly district level. The shaded error bars represent 95% credible intervals and the points indicate the median.

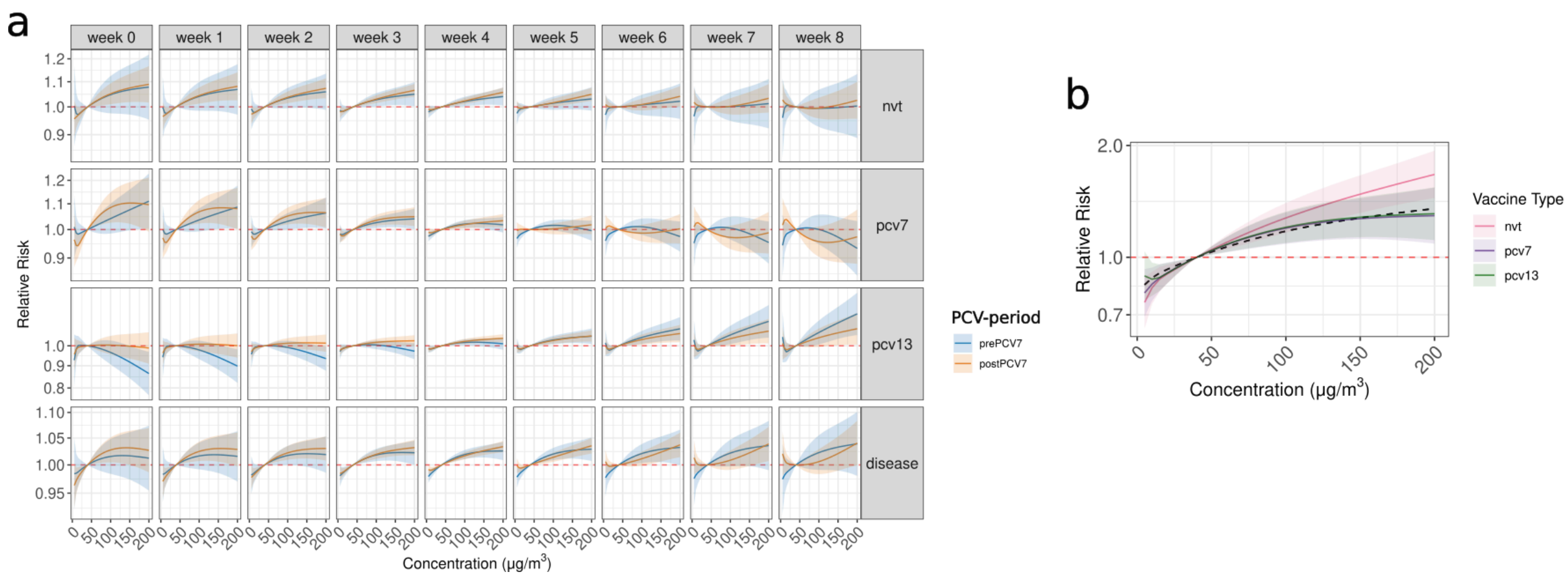

Figure S10. Exposure-response curves across different weekly exposure to $PM_{2.5}$ (concentration μg/m^3) (a) including different stratifications of invasive disease including that which is caused by NVTs (top), PCV7 (second-down), PCV13 (third-down), or across all disease cases (bottom). These are further stratified by the pre-PCV7 period (2005-2009) (blue) and the post-PCV7 period (2009-2019) (orange) in a model run at the weekly-district level (b) cumulative effect across the entire period for each vaccine group. The shaded error bars represent 95% credible intervals and the points indicate the median.

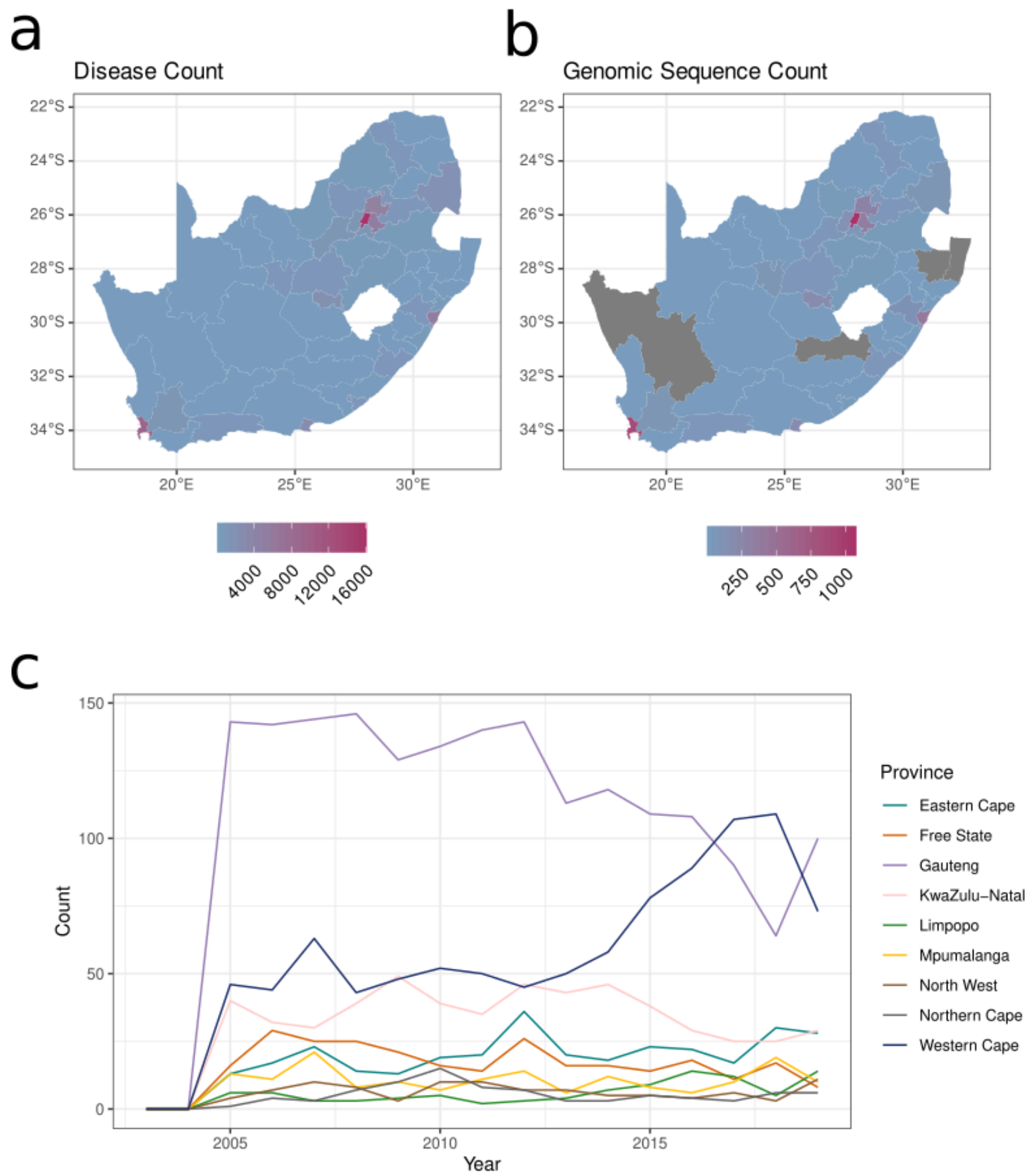

Figure S11. Summary of GPSC sequences as compared to case count and by province (a) Number of disease cases from each district and (b) the number of genomic sequences from each district. There were no sequences from 4 districts including: Namakwa, Joe Gqabi, uMkhanyakude, Zululand. (c) The number of pneumococcal samples sequenced from each province over time.

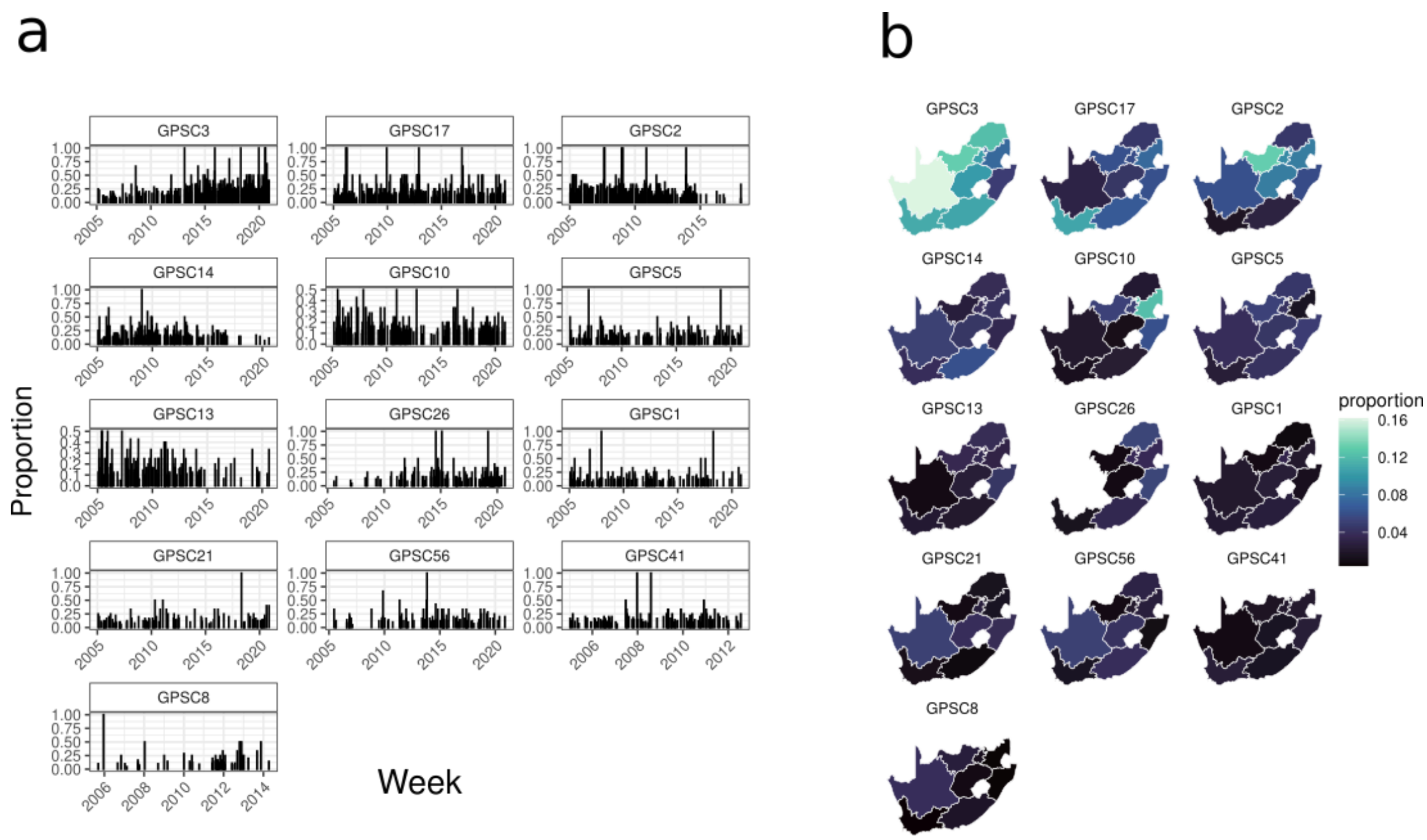

Figure S12. GPSC proportions (a) weekly proportion of each GPSC per the total number sequenced in each week across the study period (2005-2023). (b) proportion of each GPSC in each province per the total number sequenced in that province across the study period. GPSCs included are those with N≥100 as well as GPSC8 due to its epidemiological importance in South Africa.

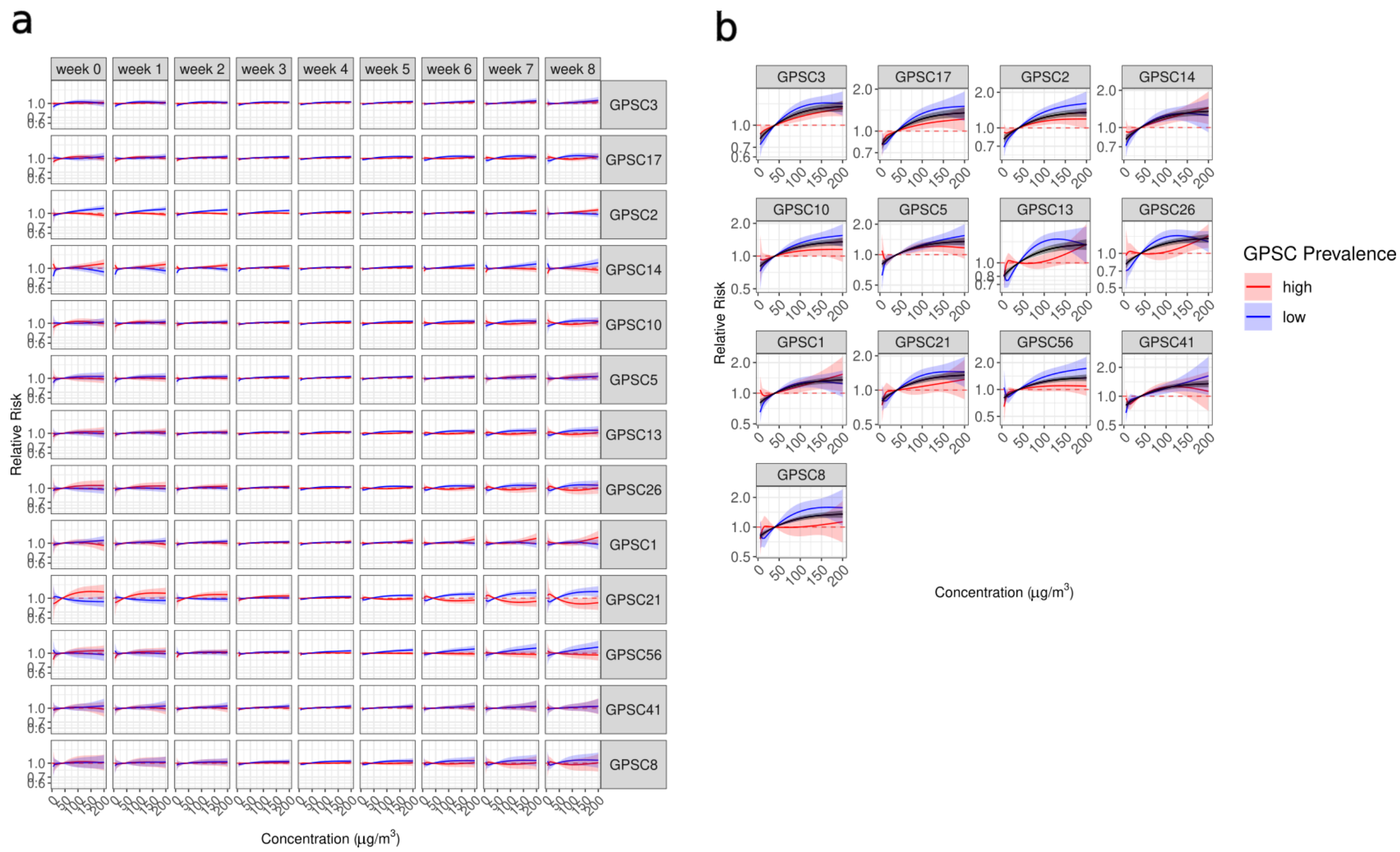

Figure S13. Exposure-response curves for $PM_{2.5}$ and pneumococcal disease interacted with the proportion of each GPSC per week per province. (a) 8-week lag time exposure-response curves and (b) includes the cumulative effect. The estimates are at high (red) and low (blue) GPSC prevalence. In b the black line indicates the model with no interaction. The shaded error bars represent 95% credible intervals and the points indicate the median.

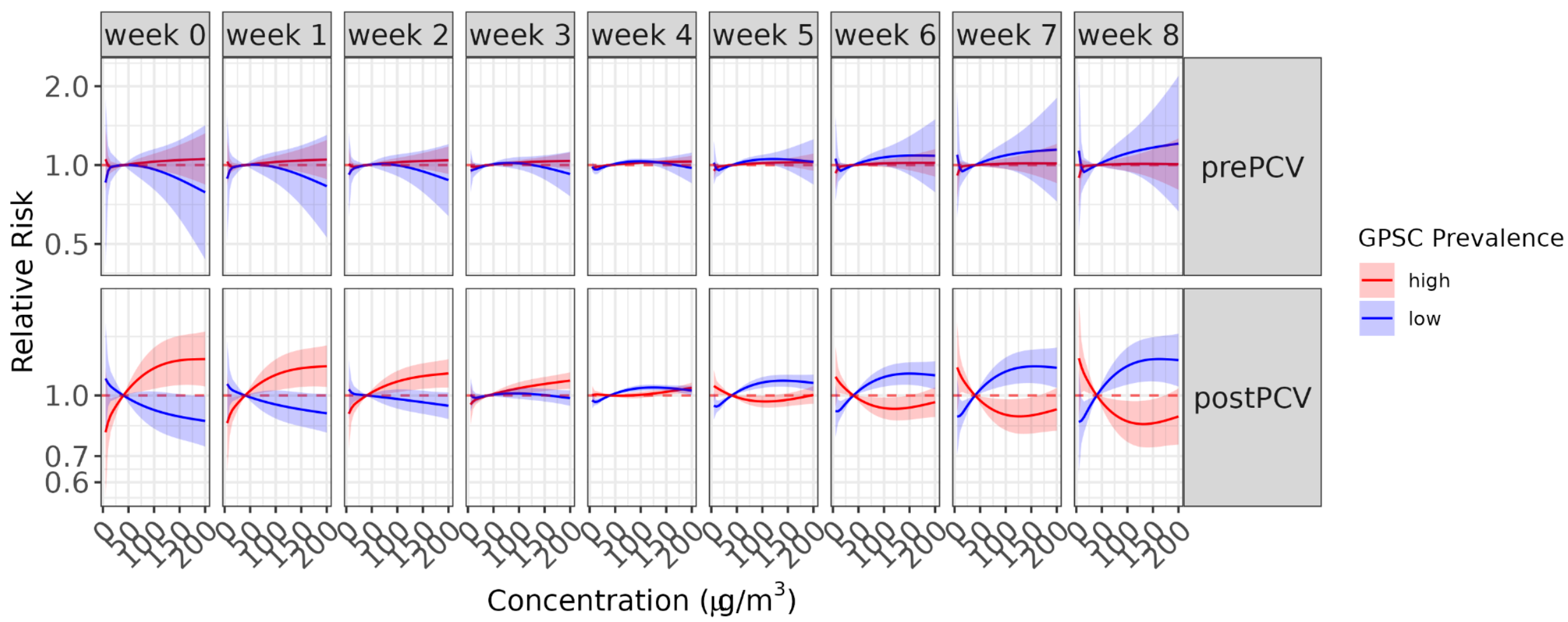

Figure S14. GPSC21-exposure-response curves for the relative risk of IPD given the concentration of $PM_{2.5}$ and interacted with the weekly proportion of GPSC21 in province level weekly models across an 8-week lag for (top) pre-PCV period 2005-2008 and (bottom) post-PCV period 2009-2019. The shaded error bars represent 95% credible intervals and the points indicate the median.

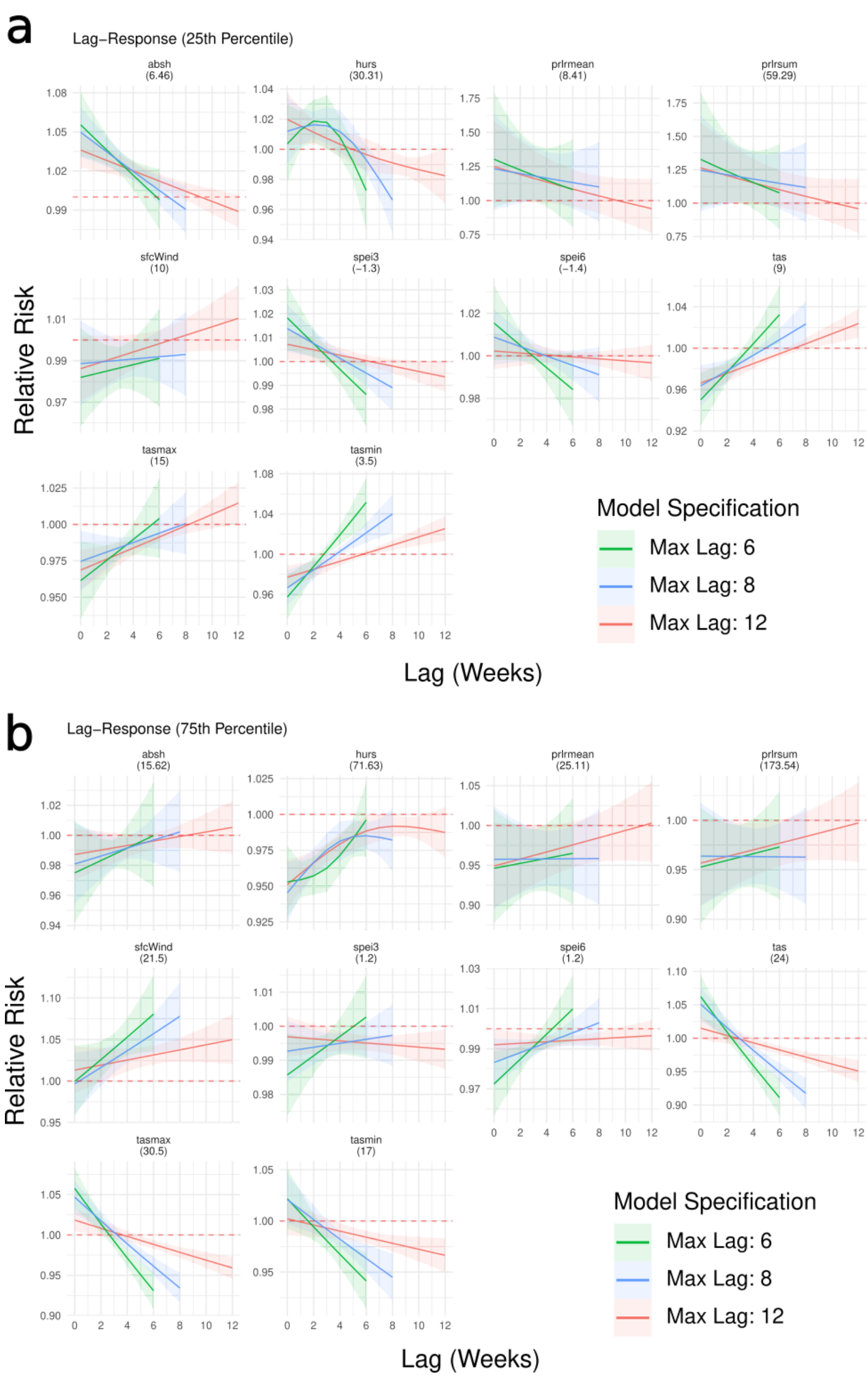

Figure S15. Marginal lag response curves at the (a) 25th percentile of the exposure variable and (b) 75th percentile of the exposure variable with maximum lags of 6 (green), 8 (blue), and 12 (pink) weeks for absolute humidity (absh, g/m^3), relative humidity (hurs, %), mean and total precipitation (prlrmean and prlrsum, millimeters), wind speed (sfcWind, km/hr), standardised precipitation and evapotranspiration index at 3 and 6 months (spei3 and spei6), and mean, maximum, and minimum temperature (tas, tasmax, tasmin; celsius). The shaded error bars represent 95% credible intervals and the points indicate the median.

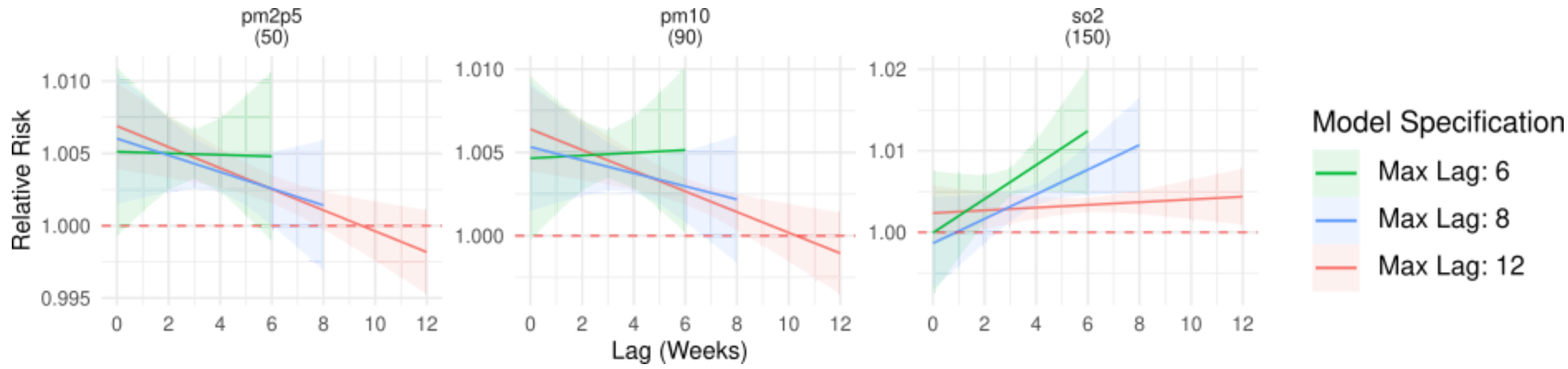

Figure S16. Marginal lag response curves for air pollution models at the 10% above the South African National Standard concentration in μg/m^3 for $PM_{2.5}$ (left), PM_{10} (middle), and SO_2 (right) with maximum lags of 6 (green), 8 (blue), and 12 (pink) weeks. The shaded error bars represent 95% credible intervals and the points indicate the median.

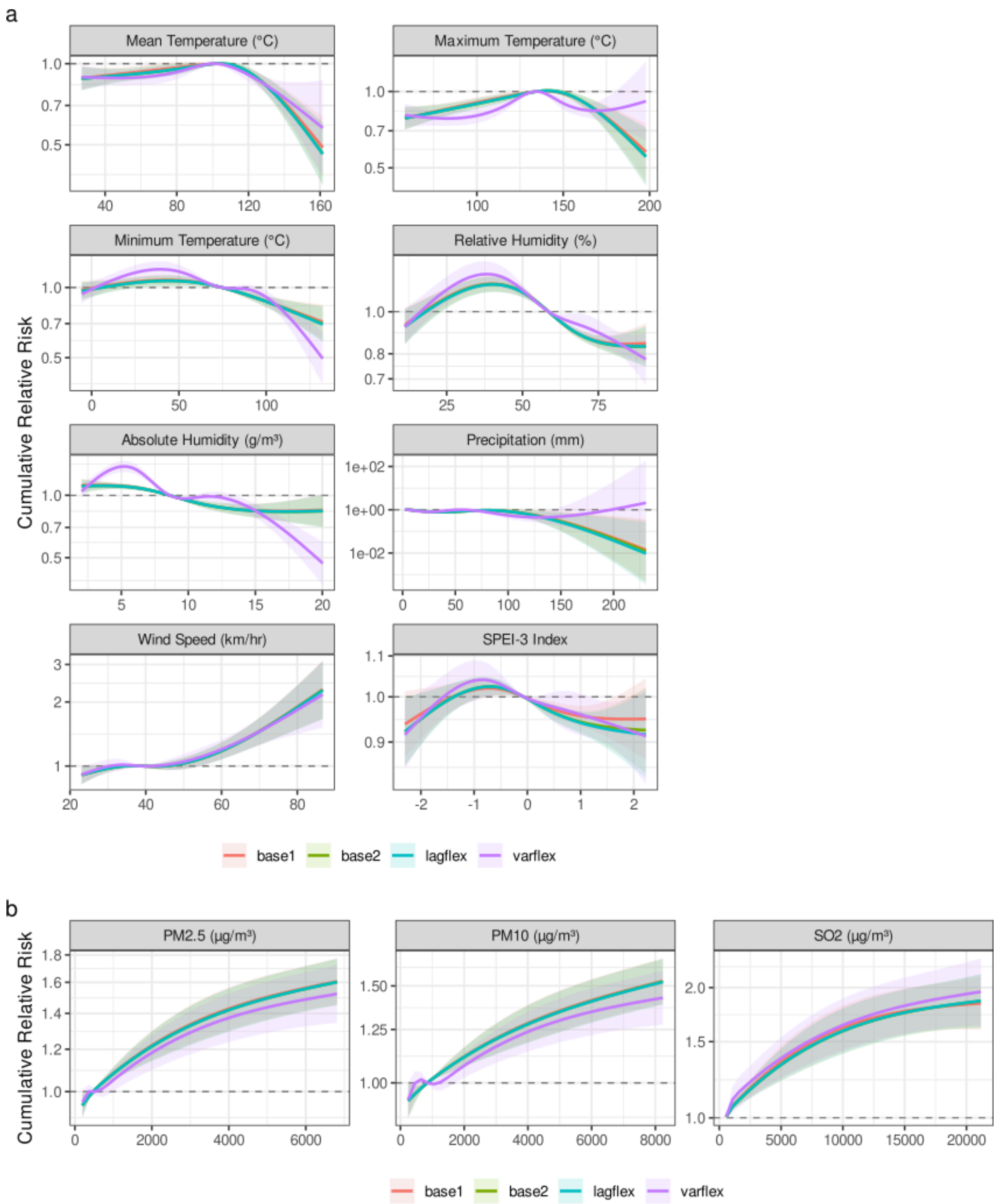

Figure S17. **DLNM spline specification sensitivity** for (a) meteorological exposures and (b) air pollution exposures with lags up to 8 weeks. Sensitivity analyses across increasing flexibility for the exposure response spline (argvar) and the exposure-lag spline (arglag). kv reflects the number of argvar degrees of freedom while kl reflects the arglag degrees of freedom. Models tested include base1 (var df = 3, lag df =2)(pink), base2 (var df =3, lag df = 3)(green), lagflex (var df =4, lag df = 5)(blue), varflex (var df =4 , lag df = 3)(purple). We include the goodness-of-fit metrics then the WAIC, MAE, -log(cpo), R^2 (Table S5). The shaded areas represent 95% credible intervals and the points indicate the median.
