# Supplementary material for: Pneumococcal population structure influences the effects of air pollution on invasive disease risk in South Africa
Source: Nat Microbiol. 2026 Sep 14;11(10):2723–37. doi: 10.1038/s41564-026-02458-5 (PMC13716828; doi:10.1038/s41564-026-02458-5)
Supplement: Supplementary file 3 [file 41564_2026_2458_MOESM3_ESM.pdf]

nature portfolio

Peer Review File

Pneumococcal population structure influences the effects of air pollution on invasive disease risk in South Africa

Corresponding Author: Dr Sophie BELMAN

Version 0:

Reviewer comments:

Reviewer #1

(Remarks to the Author)
This study is an ecological study using the cases of invasive pneumococcal disease (IPD) in nine provinces of South Africa to investigate the complex relationship between PM2.5, microbial diversity (pneumococcal serotypes and lineages), and IPD based on spatio-temporal Bayesian hierarchical models and distributed lag non-linear models. The findings identified the impact of PM2.5 with variable lag-times of the effects across pneumococcal serotypes, age groups, and disease types, and the greatest increased risk in older adults. Overall, this is a large-scale and innovative study integrating genomic surveillance data and environmental epidemiology framework. However, several major methodological concerns need to be addressed to strengthen the conclusions.

Major Concerns:
1. The environmental exposure modeling could be strengthened, particularly through more systematic inclusion of key meteorological variables in the primary model. According to the description in the section of “Base Model Evaluation”, the environmental exposure analysis relies heavily on univariable DLNMs and temperature was not included in the base model as a core exposure variable. Given the biological plausibility of temperature in affecting pathogen survival, host behavior (e.g., indoor crowding), and immune response, its absence from the primary analytical framework is a significant limitation. The observation that maximum temperature was a top univariate predictor underscores its importance, and considering temperature only in secondary analyses may underestimate its role in the primary effect structure. The meteorological variables, such as temperature and humidity are not only potential confounders but are also strongly correlated with air pollution and may interact with each other to affect human health. However, these variables were not systematically evaluated in the primary model and were mainly explored in univariable or multiplicative models. This modeling strategy may lead to residual confounding in the estimated PM2.5 effects.
2. Though the spatial random effects are included, the model may not fully account for fine-scale spatiotemporal confounding. Key environmental variables like PM2.5, humidity, and temperature are often spatialtemporally correlated. The use of province-level replication for interannual effects may mask district-specific exposure-response relationships driven by these correlated factors. In addition, environmental variable selection is primarily driven by WAIC-based model fit, with limited incorporation of prior epidemiological knowledge. A more theory-informed modeling strategy would improve interpretability.
3. Some potentially important confounders or effect modifiers, including indoor living conditions, such as household crowding and indoor airborne pathogen load, are important determinants of IPD. These factors were not considered in the present study and may contribute to residual confounding in the observed exposure-outcome associations. The authors need to evaluate the potential impact of these factors.
4. The section titled “Timing of air quality effects is mediated by lineage diversity” appears to overstate the causal role of lineage diversity, which was modeled as an effect modifier rather than an effect mediator. As described in the methods, “By incorporating the genomic lineages as a modifying proportional interaction effect” suggests the consideration of effect modification rather than effect mediation. Moreover, lineage diversity is only observed among confirmed cases and reflects pathogen characteristics post-infection, limiting its interpretability as a mediator in the causal pathway between air pollution and IPD incidence. The authors may wish to reconsider the terminology or clarify the intended causal interpretation.

Specific Points for Clarification:
1. Figure 1 suggests that stronger correlations between PM2.5 and IPD incidence are predominantly observed before approximately 2013, which coincided with major transitions in pneumococcal conjugate vaccine implementation and serotype replacement. Given the pronounced shifts from PCV7 to PCV13 and subsequently to non-vaccine serotypes, it is unclear whether the exposure-response relationship is stable over time. However, the vaccine implementation or calendar period was not explicitly modeled as an effect modifier in the main analyses. Additional interaction analyses for vaccine implementation would help assess the robustness of the findings.
2. Line 138, the selection of “an 8-week time period” (Line 138) for the DLNM lag window was stated without strong justification. While plausible for chronic effects, the biological rationale for pneumococcal infection (from exposure to invasive disease) spanning up to 8 weeks was not clearly articulated. Moreover, other important confounders should be included in the model

when exploring the lagged structure.
3. Line 144, the authors observed that "Humidity and precipitation were negatively associated with IPD risk". It is recommended to provide sufficient supporting evidence. Notably, in the South African context, extreme rainfall events, floods, or tropical cyclones may also have profound but complex impacts on infectious disease dynamics, through displacement, water contamination, and disruption of health services. The authors should consider the potential impact of extreme rainfall events and flooding.
4. The rationale for using daily maximum/minimum temperature (tasmax/ tasmin) as the temperature metrics, rather than daily mean temperature, was not provided. Different temperature metrics may capture distinct physiological stresses (e.g., heat stress vs. cold exposure). The choice should be biologically justified or comparative models should be tested.
5. Line 413, the phrase "SARS-CoV-2 pandemic" was used. For consistency and clarity within the public health and epidemiological context of this study, it is recommended to use the more common term "COVID-19 pandemic."
6. Line 443, "The population sizes per year per district were sourced from the 2023 Statistics South Africa projections". This description raises a concern about temporal appropriateness. Since the study period spans 2005-2023, applying a single set of projections from the endpoint year (2023) to represent population counts for all preceding years would be incorrect and may introduce significant inaccuracy.

(Remarks on code availability)

Reviewer #2

(Remarks to the Author)
This study stands out for its integration of large-scale epidemiological data (over 59,000 IPD cases spanning 15+ years) with genomic sequencing (4,350 isolates) and environmental variables in a high-burden setting like South Africa. An impressive study given the large number of resaerch etchniques used. While prior research has linked air pollution (PM2.5) and meteorological factors (humidity) to respiratory infections, including pneumococcal disease, the novelty here lies in explicitly accounting for microbial diversity, specifically, serotypes and genomic lineages, as mediators of environmental impacts on IPD risk. This is important for climate sensitvie disaese resaerchs.

The key issue of teh study that human activity confounds everything, pollution from industry/traffic correlates with urban density (which affects transmission), and metrology influences behavior (indoor crowding in dry seasons). The models control for density and seasonality but can't fully disentangle reverse causation or unmeasured confounders (co-infections like influenza, socioeconomic factors). The lagged effects suggest plausible mechanisms (PM2.5 impairing immunity or promoting bacterial virulence), but these are inferred, not experimentally proven.

Associations could indeed be proxies for behavioral/socioeconomic factors, and the authors emphasize this by framing results as "relationships" rather than direct causes. While not revolutionary in proving pollution "causes" IPD (ecological fallacy looms), its novelty in microbial-aware modeling makes it a step forward. Does S. Africa follow global trends, or is this just having internal validty only?

(Remarks on code availability)

Reviewer #3

(Remarks to the Author)
A large study of pneumococcal infections across a large number of hospitals over a prolonged period; it only refers to invasive infections (sepsis and meningitis mainly) and how the incidence of infection is affected by pollution and climate and how these interact with strain background. The data come from large datasets acorss South Africa over a significant period of time and the realtionship between infection, pollution, and climatic conditions looks pretty solid. The specific associations with age serotype and GPSC type are likely to be less strong due to the reduction in numbers as the comparisons are broken down within the whole research cohort; these however are the more intriguing (and perhaps more novel - I am not clear what is known about PM2.5 and pneumococus although there is a clear relation to pneumonia in some studies - this is one area the manuscript should be describe better as important background). Yet the biological basis for these associations which often differ between strains etc is not clear to me, and the authors have not really said whether they expected these patterns or whether they are unexpected.

Major issues
1. The data does not include pneumonia presumably as the microbiological diagnostic accuracy of pneumococcal pneumonia is poor, and this is not discussed at all. Yet pneumonia is the likely dominant form of pneumococcal infection and is also more obviously likely to be affected by climate. SOme text in the discussion on this I thin would be helpful, even though extrapolating from sepsis data to pneumonia must be largely intelligent guesswork. And it maybe that the increase in septicaemia largely reflects an increase in pneumonia as that is the cause of most septicaemia in adults? Although increases in other IPD is likely to be largely independent of pneumonia as they usually are not concurrent diagnoses; some sort of mechanism by which colonisation is more able to translocate into the blood or there are higher rates of colonisation?? What is known about pollution and climate and pneumococcal nasopharyngeal colonisation rates and density? The latter is important to discuss somewhere
2. The paper really needs a summary of what is already know about pollution, humidity, season, and air temperature and pneumococcal infections and other infectious diseases to make it clear what is new in this manuscript

3. Often the text lacks clarity and uses jargon that really makes it harder to follow the meaning that it could be if rewritten slightly in places. There are multiple examples of this hence I am not stating each one in this review.
4. Some discussion of the potential mechanisms underpinning differences between serotypes/GPSC, age groups, and disease in the effects of environmental factors on disease incidence would be useful, and important to indicate there is biological plausibility for some of these differences which otherwise are pretty confusing (why are older subjects more susceptible? how can pollution levels have different effects on individual pneumococcal serotypes / strains or disease type? With serotype in particualr it is odd that NVT and PCV7 serotypes cluster together and have the opposite effect on timing of their effect compared to PCV13)
5. Gauteng and Cape Town had RR of 10+ which I presume is for rates of IPD per 1000 population; this must be due to different ascertainment rates for IPD (plausible if there are large differences in the level of medical provision) as such a large variation in RR seems unlikely just due to geography / different demographics unless the vast majority of HIV +ve people live at these two locations? How much of the data are driven by the massively higher burden of infection in Gauteng and to a lesser extent Cape TOwn(what % of all cases comes from this province +/- Cape Town? would be good to state in the results), and is this increase in IPD in that province a real thing or artificial due to reduced ascertainment in other provinces? This may need reassessing the results with or without Gauteng's contribution?
6. The effects of humidity (25%) were a lot stronger than those for PM2.5(4%) - this proably should be brought up in the discussion as it is important for the reader to be aware of large differences in strength of effect between different environmental conditions

Minor issues (this is not an exhaustive list of areas of text that would benefit from some revision for clarity):
abstract lines 31-32 - it is not clear what this sentence means (the technical jargon is hard to follow)
line 35 Streptococcus pneumoniae not italicised
line 38 I think of colonisation as being the same as asymptomatic carriage so delete one or other of these
lines 46 to 47 not sure that '...in part due to the exxtensive diversity...' bit of the sentence is needed and it is not that clear how this follows the first part of the sentence in anycase
line 57 effects of lineages on pneumococcal biology really needs reference(s) (could be a review given the breadth covered by this statement)
Fig 1C and D: mark when PCVs were introduced on the graphs
Discussion lines 286-94 although i agree with the sentiment expressed here (about cuts to ART programmes), i also think it is tangential to the main data and messages from the paper and distracts from these
lines 104 are these risks presented in a table somewhere for each geographical site and also given as rates are per 1000/100,000 of the population / year? if not please make one
lines 110 to 113 i think these two sentences are connected ie the second sentence is used as evidence to suggest that trave to bigger hospitals increases the RR for large population centres? In which case the sentences best written the other way round and merged into a single sentence
lines 119-120 i can not see how ART would not reduce NVT IPD as well as VT; so was the lack of effect on NVT simply a numbers situation with too few NVT cases to show the effect of ART?
Fig 2D I am not at all sure what this is showing - it needs a better description presumably the insert is a blow up icture of the population density effect?
Line 142 NAAQs first use here - the acronym needs defining
Figure 3 and elsewhere with similar figures; the coloured shading represents the confidence intervals for these data??

(Remarks on code availability)
na

Decision Letter:

3rd February 2026

*Please ensure you delete the link to your author homepage in this e-mail if you wish to forward it to your co-authors.

Dear Dr Belman,

Thank you for your patience while your manuscript "Microbial diversity mediates the impact of air pollution on pneumococcal disease risk" was under peer-review at Nature Microbiology. It has now been seen by 3 referees, whose expertise and comments you will find at the end of this email. Although they find your work of some potential interest, they have raised a number of concerns that will need to be addressed before we can consider publication of the work in Nature Microbiology.

In particular, several referees raise concerns with potential confounders that have not been considered in your models, including temperature. We would not return to referees unless all points raised are addressed with additional analyses. If this is not possible, we would be happy to consult with our colleagues at other journals in the Portfolio to see if there is a more straightforward path to publication.

Should further experimental data allow you to address these criticisms, we would be happy to look at a revised manuscript.

We are committed to providing a fair and constructive peer-review process. Please do not hesitate to contact us if there are specific requests from the reviewers that you believe are technically impossible or unlikely to yield a meaningful outcome.

We strongly support public availability of data. Please place the data used in your paper into a public data repository, if one exists, or alternatively, present the data as Source Data or Supplementary Information. If data can only be shared on request, please explain why in your Data Availability Statement, and also in the correspondence with your editor. For some data types,

deposition in a public repository is mandatory - more information on our data deposition policies and available repositories can be found at https://www.nature.com/nature-research/editorial-policies/reporting-standards#availability-of-data.

Please include a data availability statement as a separate section after Methods but before references, under the heading "Data Availability". This section should inform readers about the availability of the data used to support the conclusions of your study. This information includes accession codes to public repositories (data banks for protein, DNA or RNA sequences, microarray, proteomics data etc…), references to source data published alongside the paper, unique identifiers such as URLs to data repository entries, or data set DOIs, and any other statement about data availability. At a minimum, you should include the following statement: “The data that support the findings of this study are available from the corresponding author upon request”, mentioning any restrictions on availability. If DOIs are provided, we also strongly encourage including these in the Reference list (authors, title, publisher (repository name), identifier, year). For more guidance on how to write this section please see: http://www.nature.com/authors/policies/data/data-availability-statements-data-citations.pdf

If revising your manuscript:

* Include a “Response to referees” document detailing, point-by-point, how you addressed each referee comment. If no action was taken to address a point, you must provide a compelling argument. This response will be sent back to the referees along with the revised manuscript.

* If you have not done so already we suggest that you begin to revise your manuscript so that it conforms to our Article format instructions at http://www.nature.com/nmicrobiol/info/final-submission. Refer also to any guidelines provided in this letter.

* Include a revised version of any required reporting checklist. It will be available to referees (and, potentially, statisticians) to aid in their evaluation if the manuscript goes back for peer review. A revised checklist is essential for re-review of the paper.

When submitting the revised version of your manuscript, please pay close attention to our href="https://www.nature.com/nature-portfolio/editorial-policies/image-integrity">Digital Image Integrity Guidelines. and to the following points below:

-- that unprocessed scans are clearly labelled and match the gels and western blots presented in figures.
-- that control panels for gels and western blots are appropriately described as loading on sample processing controls
-- all images in the paper are checked for duplication of panels and for splicing of gel lanes.

EXTENDED DATA FIGURES

When re-submitting your manuscript, please ensure that any supplementary figures and tables that are crucial to the manuscript’s conclusions are converted into Extended Data figures and tables to increase visibility of these data. Extended Data figures and tables are online-only (present in the online PDF and full-text HTML versions of the paper), peer-reviewed display items that provide essential background to the article but are not included in the main article due to space constraints. A maximum of ten Extended Data display items (figures and tables) is permitted.

Finally, please ensure that you retain unprocessed data and metadata files after publication, ideally archiving data in perpetuity, as these may be requested during the peer review and production process or after publication if any issues arise.

Please use the link below to submit a revised paper:

Link Redacted

Note: This url links to your confidential homepage and associated information about manuscripts you may have submitted or be reviewing for us. If you wish to forward this e-mail to co-authors, please delete this link to your homepage first.

Nature Microbiology is committed to improving transparency in authorship. As part of our efforts in this direction, we are now requesting that all authors identified as ‘corresponding author’ on published papers create and link their Open Researcher and Contributor Identifier (ORCID) with their account on the Manuscript Tracking System (MTS), prior to acceptance. This applies to primary research papers only. ORCID helps the scientific community achieve unambiguous attribution of all scholarly contributions. You can create and link your ORCID from the home page of the MTS by clicking on ‘Modify my Springer Nature account’. For more information please visit please visit www.springernature.com/orcid.

If you wish to submit a suitably revised manuscript we would hope to receive it within 6 months. If you cannot send it within this time, please let us know. We will be happy to consider your revision, even if a similar study has been accepted for publication at Nature Microbiology or published elsewhere (up to a maximum of 6 months).

In the meantime we hope that you find our referees' comments helpful.

Yours sincerely,

****

Reviewer Expertise:

Referee #1: air pollution, epidemiology, modelling
Referee #2: environmental health and epidemiology, climate change and pollution
Referee #3: Pneumococcal infections, clinical

Reviewer Comments:

Reviewer #1 (Remarks to the Author):

This study is an ecological study using the cases of invasive pneumococcal disease (IPD) in nine provinces of South Africa to investigate the complex relationship between PM2.5, microbial diversity (pneumococcal serotypes and lineages), and IPD based on spatio-temporal Bayesian hierarchical models and distributed lag non-linear models. The findings identified the impact of PM2.5 with variable lag-times of the effects across pneumococcal serotypes, age groups, and disease types, and the greatest increased risk in older adults. Overall, this is a large-scale and innovative study integrating genomic surveillance data and environmental epidemiology framework. However, several major methodological concerns need to be addressed to strengthen the conclusions.

Major Concerns:
1. The environmental exposure modeling could be strengthened, particularly through more systematic inclusion of key meteorological variables in the primary model. According to the description in the section of “Base Model Evaluation”, the environmental exposure analysis relies heavily on univariable DLNMs and temperature was not included in the base model as a core exposure variable. Given the biological plausibility of temperature in affecting pathogen survival, host behavior (e.g., indoor crowding), and immune response, its absence from the primary analytical framework is a significant limitation. The observation that maximum temperature was a top univariate predictor underscores its importance, and considering temperature only in secondary analyses may underestimate its role in the primary effect structure. The meteorological variables, such as temperature and humidity are not only potential confounders but are also strongly correlated with air pollution and may interact with each other to affect human health. However, these variables were not systematically evaluated in the primary model and were mainly explored in univariable or multiplicative models. This modeling strategy may lead to residual confounding in the estimated PM2.5 effects.
2. Though the spatial random effects are included, the model may not fully account for fine-scale spatiotemporal confounding. Key environmental variables like PM2.5, humidity, and temperature are often spatialtemporally correlated. The use of province-level replication for interannual effects may mask district-specific exposure-response relationships driven by these correlated factors. In addition, environmental variable selection is primarily driven by WAIC-based model fit, with limited incorporation of prior epidemiological knowledge. A more theory-informed modeling strategy would improve interpretability.
3. Some potentially important confounders or effect modifiers, including indoor living conditions, such as household crowding and indoor airborne pathogen load, are important determinants of IPD. These factors were not considered in the present study and may contribute to residual confounding in the observed exposure-outcome associations. The authors need to evaluate the potential impact of these factors.
4. The section titled “Timing of air quality effects is mediated by lineage diversity” appears to overstate the causal role of lineage diversity, which was modeled as an effect modifier rather than an effect mediator. As described in the methods, “By incorporating the genomic lineages as a modifying proportional interaction effect” suggests the consideration of effect modification rather than effect mediation. Moreover, lineage diversity is only observed among confirmed cases and reflects pathogen characteristics post-infection, limiting its interpretability as a mediator in the causal pathway between air pollution and IPD incidence. The authors may wish to reconsider the terminology or clarify the intended causal interpretation.

Specific Points for Clarification:
1. Figure 1 suggests that stronger correlations between PM2.5 and IPD incidence are predominantly observed before approximately 2013, which coincided with major transitions in pneumococcal conjugate vaccine implementation and serotype replacement. Given the pronounced shifts from PCV7 to PCV13 and subsequently to non-vaccine serotypes, it is unclear whether the exposure-response relationship is stable over time. However, the vaccine implementation or calendar period was not explicitly modeled as an effect modifier in the main analyses. Additional interaction analyses for vaccine implementation would help assess the robustness of the findings.
2. Line 138, the selection of “an 8-week time period” (Line 138) for the DLNM lag window was stated without strong justification. While plausible for chronic effects, the biological rationale for pneumococcal infection (from exposure to invasive disease) spanning up to 8 weeks was not clearly articulated. Moreover, other important confounders should be included in the model when exploring the lagged structure.
3. Line 144, the authors observed that “Humidity and precipitation were negatively associated with IPD risk”. It is recommended to provide sufficient supporting evidence. Notably, in the South African context, extreme rainfall events, floods, or tropical cyclones may also have profound but complex impacts on infectious disease dynamics, through displacement, water contamination, and disruption of health services. The authors should consider the potential impact of extreme rainfall events and flooding.
4. The rationale for using daily maximum/minimum temperature (tasmax/ tasmin) as the temperature metrics, rather than daily mean temperature, was not provided. Different temperature metrics may capture distinct physiological stresses (e.g., heat stress vs. cold exposure). The choice should be biologically justified or comparative models should be tested.
5. Line 413, the phrase “SARS-CoV-2 pandemic” was used. For consistency and clarity within the public health and epidemiological context of this study, it is recommended to use the more common term “COVID-19 pandemic.”
6. Line 443, “The population sizes per year per district were sourced from the 2023 Statistics South Africa projections”. This description raises a concern about temporal appropriateness. Since the study period spans 2005-2023, applying a single set of projections from the endpoint year (2023) to represent population counts for all preceding years would be incorrect and may

introduce significant inaccuracy.

Reviewer #2 (Remarks to the Author):

This study stands out for its integration of large-scale epidemiological data (over 59,000 IPD cases spanning 15+ years) with genomic sequencing (4,350 isolates) and environmental variables in a high-burden setting like South Africa. An impressive study given the large number of resaerch etchniques used. While prior research has linked air pollution (PM2.5) and meteorological factors (humidity) to respiratory infections, including pneumococcal disease, the novelty here lies in explicitly accounting for microbial diversity, specifically, serotypes and genomic lineages, as mediators of environmental impacts on IPD risk. This is important for climate sensitvie disaese resaerchs.

The key issue of teh study that human activity confounds everything, pollution from industry/traffic correlates with urban density (which affects transmission), and metrology influences behavior (indoor crowding in dry seasons). The models control for density and seasonality but can't fully disentangle reverse causation or unmeasured confounders (co-infections like influenza, socioeconomic factors). The lagged effects suggest plausible mechanisms (PM2.5 impairing immunity or promoting bacterial virulence), but these are inferred, not experimentally proven.

Associations could indeed be proxies for behavioral/socioeconomic factors, and the authors emphasize this by framing results as "relationships" rather than direct causes. While not revolutionary in proving pollution "causes" IPD (ecological fallacy looms), its novelty in microbial-aware modeling makes it a step forward. Does S. Africa follow global trends, or is this just having internal validty only?

Reviewer #3 (Remarks to the Author):

A large study of pneumococcal infections across a large number of hospitals over a prolonged period; it only refers to invasive infections (sepsis and meningitis mainly) and how the incidence of infection is affected by pollution and climate and how these interact with strain background. The data come from large datasets acorss South Africa over a significant period of time and the realtionship between infection, pollution, and climatic conditions looks pretty solid. The specific associations with age serotype and GPSC type are likely to be less strong due to the reduction in numbers as the comparisons are broken down within the whole research cohort; these however are the more intriguing (and perhaps more novel - I am not clear what is known about PM2.5 and pneumococus although there is a clear relation to pneumonia in some studies - this is one area the manuscript should be describe better as important background). Yet the biological basis for these associations which often differ between strains etc is not clear to me, and the authors have not really said whether they expected these patterns or whether they are unexpected.

Major issues
1. The data does not include pneumonia presumably as the microbiological diagnostic accuracy of pneumococcal pneumonia is poor, and this is not discussed at all. Yet pneumonia is the likely dominant form of pneumococcal infection and is also more obviously likely to be affected by climate. SOme text in the discussion on this I thin would be helpful, even though extrapolating from sepsis data to pneumonia must be largely intelligent guesswork. And it maybe that the increase in septicaemia largely reflects an increase in pneumonia as that is the cause of most septicaemia in adults? Although increases in other IPD is likely to be largely independent of pneumonia as they usually are not concurrent diagnoses; some sort of mechanism by which colonisation is more able to translocate into the blood or there are higher rates of colonisation?? What is known about pollution and climate and pneumococcal nasopharyngeal colonisation rates and density? The latter is important to discuss somewhere
2. The paper really needs a summary of what is already know about pollution, humidity, season, and air temperature and pneumococcal infections and other infectious diseases to make it clear what is new in this manuscript
3. Often the text lacks clarity and uses jargon that really makes it harder to follow the meaning that it could be if rewritten slightly in places. There are multiple examples of this hence I am not stating each one in this review.
4. Some discussion of the potential mechanisms underpinning differences between serotypes/GPSC, age groups, and disease in the effects of environmental factors on disease incidence would be useful, and important to indicate there is biological plausibility for some of these differences which otherwise are pretty confusing (why are older subjects more susceptible? how can pollution levels have different effects on individual pneumococcal serotypes / strains or disease type? With serotype in particualr it is odd that NVT and PCV7 serotypes cluster together and have the opposite effect on timing of their effect compared to PCV13)
5. Gauteng and Cape Town had RR of 10+ which I presume is for rates of IPD per 1000 population; this must be due to different ascertainment rates for IPD (plausible if there are large differences in the level of medical provision) as such a large variation in RR seems unlikely just due to geography / different demographics unless the vast majority of HIV +ve people live at these two locations? How much of the data are driven by the massively higher burden of infection in Gauteng and to a lesser extent Cape TOwn(what % of all cases comes from this province +/- Cape Town? would be good to state in the results), and is this increase in IPD in that province a real thing or artificial due to reduced ascertainment in other provinces? This may need reassessing the results with or without Gauteng's contribution?
6. The effects of humidity (25%) were a lot stronger than those for PM2.5(4%) - this proably should be brought up in the discussion as it is important for the reader to be aware of large differences in strength of effect between different environmental conditions

Minor issues (this is not an exhaustive list of areas of text that would benefit from some revision for clarity):

abstract lines 31-32 - it is not clear what this sentence means (the technical jargon is hard to follow)
line 35 Streptococcus pneumoniae not italicised
line 38 I think of colonisation as being the same as asymptomatic carriage so delete one or other of these
lines 46 to 47 not sure that '...in part due to the exxtensive diversity...' bit of the sentence is needed and it is not that clear how this follows the first part of the sentence in anycase
line 57 effects of lineages on pneumococcal biology really needs reference(s) (could be a review given the breadth covered by this statement)
Fig 1C and D: mark when PCVs were introduced on the graphs
Discussion lines 286-94 although i agree with the sentiment expressed here (about cuts to ART programmes), i also think it is tangential to the main data and messages from the paper and distracts from these
lines 104 are these risks presented in a table somewhere for each geographical site and also given as rates are per 1000/100,000 of the population / year? if not please make one
lines 110 to 113 i think these two sentences are connected ie the second sentence is used as evidence to suggest that trave to bigger hospitals increases the RR for large population centres? In which case the sentences best written the other way round and merged into a single sentence
lines 119-120 i can not see how ART would not reduce NVT IPD as well as VT; so was the lack of effect on NVT simply a numbers situation with too few NVT cases to show the effect of ART?
Fig 2D I am not at all sure what this is showing - it needs a better description presumably the insert is a blow up icture of the population density effect?
Line 142 NAAQs first use here - the acronym needs defining
Figure 3 and elsewhere with similar figures; the coloured shading represents the confidence intervals for these data??

Reviewer #3 (Remarks on code availability):

na

****

Version 1:

Reviewer comments:

Reviewer #1

(Remarks to the Author)
The authors have revised and restructured the modelling strategy, which has addressed many core concerns. I have a few remaining suggestions regarding the statistical modelling and technical details.

1. The description in the Methods remains brief regarding the DLNM specification for meteorological variables. Please provide a more detailed specification consistent with the GitHub code, cite references for the arglag/argvar choices and conduct the corresponding sensitivity analyses. Furthermore, I recommend showing the lag-response structure for the main meteorological exposures in the supplementary material to support the selected maximum lag period (e.g. supplementary figures for the lag-response surfaces or marginal lag curves).
2. The authors mentioned that "For each variable we included equally distributed knots at the 33rd and 67th quantile except for precipitation for which we included 2 equally spaced knots on a log scale of the precipitation distribution. We centered and scaled all meteorological factors. We centered the effect estimation at the median of the variable distribution (Table S5)." In the GitHub code, I noticed that "if(cov_oi%in%c("pm2p5_lag0","pm10_lag0","o3_lag0","so2_lag0")){
if(cov_oi=="pm2p5_lag0"){ cen = 40 }
if(cov_oi=="pm10_lag0"){ cen = 75 }
# if(cov_oi=="o3_lag0"){ cen = 100 }
if(cov_oi=="so2_lag0"){ cen = 125 }
}";
Many air pollution epidemiological studies assume a linear no-threshold concentration–response relationship and therefore use the minimum observed concentration or a very low value as the reference. The rationale for choosing 40, 75, 100 and 125 should be explicitly provided. Similarly, the chosen reference values for temperature and relative humidity should be justified in the manuscript.
3. In Figure 3a, there are segments of the exposure–response curves at high temperatures where only point estimates appear to be plotted without credible intervals. Could the authors clarify why the intervals are absent for some portions of the curves? Additionally, according to Figure 3a, Additionally, both extremely high and low temperatures appear to show a protective effect against IPD. Is there a plausible biological rationale for this? The authors should briefly discuss this finding.
4. In Figures 4b, 4c and 4d, the credible interval for the "all disease" group is barely distinguishable. I suggest using a different visual mark to make these intervals visible. Additionally, in Figure 4b, the vertical dashed reference line appears to be drawn at approximately 40 μg/m³ rather than at the 50 μg/m³ stated in the text. Please verify and correct if necessary.
5. The Methods indicate that "The population size per year per district (from 2005–2023), were sourced from the 2023 Statistics South Africa projections." I was unable to find yearly population estimates at the district level for the full study period in the cited Statistics South Africa Annual Report 2023 (https://www.statssa.gov.za/publications/AnnualReport/AR_Book1202324.pdf). Please double-check the data source and, if necessary, provide a more precise citation or clarify the dataset from which the annual district populations were obtained.

(Remarks on code availability)
See the remarks to be sent to the author.

Reviewer #2

(Remarks to the Author)
Belman et al. provide compelling evidence that particulate matter and humidity exert non-linear, lagged effects on IPD incidence, with microbial diversity—serotypes and genomic lineages—acting as key effect modifiers.

Monthly invasive pneumococcal disease case counts visibly peak during the colder months, coinciding precisely with seasonal elevations in mean PM concentrations and inversely tracking relative humidity levels. This visual co-variation directly supports the manuscript's central claims that (i) air pollution is positively associated with IPD risk and humidity exerts a protective effect at higher levels.

The authors also performed a simple lag analysis (Spearman correlations of environmental exposures with disease outcomes) that identified maximum associations up to approximately 5 weeks, providing an empirical basis for the 8-week distributed lag window used in the models.

'Looking at the data' yields clear confirmatory evidence for the direction, seasonality, and urban concentration of the associations.

Moreover, the manuscript has a more nuanced, policy-relevant questions and answer.

Weekly PM at 50 ug (exceeding South Africa's NAAQS threshold) confers a ~4% cumulative 8-week risk increase (up to 18% at 100 ug)

The authors have also addressed my earlier points on potential confounding by human activity (urban density, indoor crowding, behavioral factors) through explicit discussion of limitations, use of population density as a proxy, and spatiotemporal random effects. They appropriately frame results as observational associations rather than direct causation and provide mechanistic hypotheses grounded in the literature (epithelial adhesion, biofilm formation, serotype-specific virulence factors such as psrP).

I recommend this publication. J

(Remarks on code availability)

Reviewer #3

(Remarks to the Author)
the abstract does not seem to mention the most novel finding of the paper which is the effects of pollution differ between pneumococcal serotypes / lineages which seems a major oversight.

last three or four sentences of the abstract really could do with some tweaking eg add 'moderate' in front of Humidity (33-49%), the next sentence really needs a bit of fiddling with the last part to make it clearer/ better use of English, and the last sentence I would rewrite completely as I really not sure what it is saying - surely this paper has quantified these relationships? and ~I am not sure their findings will affect vaccine policies, these are too crude to cope with the changes seen here; but this paper is good evidence that pollution is bad and adds to the wealth of data that suggests controlling pollution will be immensely important for public health.

Page 2 'accounting for' phrasing occurs in consecutive sentences

Meterological section in the discussion of temperature it seems cold moderate and high temperatures all increase risk; so what is the comparative temperature with lower risk? this is all bit confusing to the reader and needs a bit more text to make clearer why all three temperature bands have an increased risk

(Remarks on code availability)
not an expert so pointless for me to review the code

Decision Letter:

5th May 2026

*Please ensure you delete the link to your author homepage in this e-mail if you wish to forward it to your co-authors.

Dear Sophie,

Thank you for your patience while your manuscript "Microbial diversity modifies the impact of air pollution on pneumococcal disease risk" was under peer-review at Nature Microbiology. It has now been seen by 3 referees, whose expertise and comments you will find at the end of this email. You will see from their comments below that while they find your work of interest,

some important points are raised. We are very interested in the possibility of publishing your study in Nature Microbiology, but would like to consider your response to these concerns in the form of a revised manuscript before we make a final decision on publication.

In particular, you will see that referee #1 has some comments that should be straightforward to address.

We are committed to providing a fair and constructive peer-review process. Do not hesitate to contact us if there are specific requests from the reviewers that you believe are technically impossible or unlikely to yield a meaningful outcome.

If you have not done so already please begin to revise your manuscript so that it conforms to our Article format instructions at http://www.nature.com/nmicrobiol/info/final-submission/

The usual length limit for a Nature Microbiology Article is six display items (figures or tables) and 3,000 words. We have some flexibility, and can allow a revised manuscript at 3,500 words, but please consider this a firm upper limit. There is a trade-off of ~250 words per display item, so if you need more space, you could move a Figure or Table to Supplementary Information.

Some reduction could be achieved by focusing any introductory material and moving it to the start of your opening 'bold' paragraph, whose function is to outline the background to your work, describe in a sentence your new observations, and explain your main conclusions. The discussion should also be limited. Methods should be described in a separate section following the discussion, we do not place a word limit on Methods.

Nature Microbiology titles should give a sense of the main new findings of a manuscript, and should not contain punctuation. Please keep in mind that we strongly discourage active verbs in titles, and that they should ideally fit within 90 characters each (including spaces).

We strongly support public availability of data. Please place the data used in your paper into a public data repository, if one exists, or alternatively, present the data as Source Data or Supplementary Information. If data can only be shared on request, please explain why in your Data Availability Statement, and also in the correspondence with your editor. For some data types, deposition in a public repository is mandatory - more information on our data deposition policies and available repositories can be found at https://www.nature.com/nature-research/editorial-policies/reporting-standards#availability-of-data.

Please include a data availability statement as a separate section after Methods but before references, under the heading "Data Availability". This section should inform readers about the availability of the data used to support the conclusions of your study. This information includes accession codes to public repositories (data banks for protein, DNA or RNA sequences, microarray, proteomics data etc…), references to source data published alongside the paper, unique identifiers such as URLs to data repository entries, or data set DOIs, and any other statement about data availability. At a minimum, you should include the following statement: "The data that support the findings of this study are available from the corresponding author upon request", mentioning any restrictions on availability. If DOIs are provided, we also strongly encourage including these in the Reference list (authors, title, publisher (repository name), identifier, year). For more guidance on how to write this section please see: http://www.nature.com/authors/policies/data/data-availability-statements-data-citations.pdf

To improve the accessibility of your paper to readers from other research areas, please pay particular attention to the wording of the paper's opening bold paragraph, which serves both as an introduction and as a brief, non-technical summary in about 150 words. If, however, you require one or two extra sentences to explain your work clearly, please include them even if the paragraph is over-length as a result. The opening paragraph should not contain references. Because scientists from other sub-disciplines will be interested in your results and their implications, it is important to explain essential but specialised terms concisely. We suggest you show your summary paragraph to colleagues in other fields to uncover any problematic concepts.

If your paper is accepted for publication, we will edit your display items electronically so they conform to our house style and will reproduce clearly in print. If necessary, we will re-size figures to fit single or double column width. If your figures contain several parts, the parts should form a neat rectangle when assembled. Choosing the right electronic format at this stage will speed up the processing of your paper and give the best possible results in print. We would like the figures to be supplied as vector files - EPS, PDF, AI or postscript (PS) file formats (not raster or bitmap files), preferably generated with vector-graphics software (Adobe Illustrator for example). Please try to ensure that all figures are non-flattened and fully editable. All images should be at least 300 dpi resolution (when figures are scaled to approximately the size that they are to be printed at) and in RGB colour format. Please do not submit Jpeg or flattened TIFF files. Please see also 'Guidelines for Electronic Submission of Figures' at the end of this letter for further detail.

Figure legends must provide a brief description of the figure and the symbols used, within 350 words, including definitions of any error bars employed in the figures.

When submitting the revised version of your manuscript, please pay close attention to our href="https://www.nature.com/nature-research/editorial-policies/image-integrity">Digital Image Integrity Guidelines. and to the following points below:

-- that unprocessed scans are clearly labelled and match the gels and western blots presented in figures.
-- that control panels for gels and western blots are appropriately described as loading on sample processing controls
-- all images in the paper are checked for duplication of panels and for splicing of gel lanes.

EXTENDED DATA FIGURES

When re-submitting your manuscript, please ensure that any supplementary figures and tables that are crucial to the

manuscript’s conclusions are converted into Extended Data figures and tables to increase visibility of these data. Extended Data figures and tables are online-only (present in the online PDF and full-text HTML versions of the paper), peer-reviewed display items that provide essential background to the article but are not included in the main article due to space constraints. A maximum of ten Extended Data display items (figures and tables) is permitted.

Finally, please ensure that you retain unprocessed data and metadata files after publication, ideally archiving data in perpetuity, as these may be requested during the peer review and production process or after publication if any issues arise.

Please include a statement before the acknowledgements naming the author to whom correspondence and requests for materials should be addressed.

Finally, we require authors to include a statement of their individual contributions to the paper -- such as experimental work, project planning, data analysis, etc. -- immediately after the acknowledgements. The statement should be short, and refer to authors by their initials. For details please see the Authorship section of our joint Editorial policies at http://www.nature.com/authors/editorial_policies/authorship.html

When revising your paper:

* include a point-by-point response to any editorial suggestions and to our referees. Please include your response to the editorial suggestions in your cover letter, and please upload your response to the referees as a separate document.

* ensure it complies with our format requirements for Letters as set out in our guide to authors at www.nature.com/nmicrobiol/info/gta/

* state in a cover note the length of the text, methods and legends; the number of references; number and estimated final size of figures and tables

* resubmit electronically if possible using the link below to access your home page:

Link Redacted

*This url links to your confidential homepage and associated information about manuscripts you may have submitted or be reviewing for us. If you wish to forward this e-mail to co-authors, please delete this link to your homepage first.

Please ensure that all correspondence is marked with your Nature Microbiology reference number in the subject line.

Nature Microbiology is committed to improving transparency in authorship. As part of our efforts in this direction, we are now requesting that all authors identified as ‘corresponding author’ on published papers create and link their Open Researcher and Contributor Identifier (ORCID) with their account on the Manuscript Tracking System (MTS), prior to acceptance. This applies to primary research papers only. ORCID helps the scientific community achieve unambiguous attribution of all scholarly contributions. You can create and link your ORCID from the home page of the MTS by clicking on ‘Modify my Springer Nature account’. For more information please visit please visit www.springernature.com/orcid.

We hope to receive your revised paper within three weeks. If you cannot send it within this time, please let us know.

We look forward to hearing from you soon.

Yours sincerely,

****

Reviewers Comments:

Reviewer #1 (Remarks to the Author):

The authors have revised and restructured the modelling strategy, which has addressed many core concerns. I have a few remaining suggestions regarding the statistical modelling and technical details.

1. The description in the Methods remains brief regarding the DLNM specification for meteorological variables. Please provide a more detailed specification consistent with the GitHub code, cite references for the arglag/argvar choices and conduct the corresponding sensitivity analyses. Furthermore, I recommend showing the lag-response structure for the main meteorological exposures in the supplementary material to support the selected maximum lag period (e.g. supplementary figures for the lag-response surfaces or marginal lag curves).
2. The authors mentioned that “For each variable we included equally distributed knots at the 33rd and 67th quantile except for precipitation for which we included 2 equally spaced knots on a log scale of the precipitation distribution. We centered and scaled all meteorological factors. We centered the effect estimation at the median of the variable distribution (Table S5).” In the

GitHub code, I noticed that “if(cov_oi%in%c("pm2p5_lag0","pm10_lag0","o3_lag0","so2_lag0")){
if(cov_oi=="pm2p5_lag0"){ cen = 40 }
if(cov_oi=="pm10_lag0"){ cen = 75 }
# if(cov_oi=="o3_lag0"){ cen = 100 }
if(cov_oi=="so2_lag0"){ cen = 125 }
}”;
Many air pollution epidemiological studies assume a linear no-threshold concentration–response relationship and therefore use the minimum observed concentration or a very low value as the reference. The rationale for choosing 40, 75, 100 and 125 should be explicitly provided. Similarly, the chosen reference values for temperature and relative humidity should be justified in the manuscript.
3. In Figure 3a, there are segments of the exposure–response curves at high temperatures where only point estimates appear to be plotted without credible intervals. Could the authors clarify why the intervals are absent for some portions of the curves? Additionally, according to Figure 3a, Additionally, both extremely high and low temperatures appear to show a protective effect against IPD. Is there a plausible biological rationale for this? The authors should briefly discuss this finding.
4. In Figures 4b, 4c and 4d, the credible interval for the “all disease” group is barely distinguishable. I suggest using a different visual mark to make these intervals visible. Additionally, in Figure 4b, the vertical dashed reference line appears to be drawn at approximately 40 µg/m³ rather than at the 50 µg/m³ stated in the text. Please verify and correct if necessary.
5. The Methods indicate that “The population size per year per district (from 2005–2023), were sourced from the 2023 Statistics South Africa projections.” I was unable to find yearly population estimates at the district level for the full study period in the cited Statistics South Africa Annual Report 2023 (https://www.statssa.gov.za/publications/AnnualReport/AR_Book1202324.pdf). Please double-check the data source and, if necessary, provide a more precise citation or clarify the dataset from which the annual district populations were obtained.

Reviewer #1 (Remarks on code availability):

See the remarks to be sent to the author.

Reviewer #2 (Remarks to the Author):

Belman et al. provide compelling evidence that particulate matter and humidity exert non-linear, lagged effects on IPD incidence, with microbial diversity—serotypes and genomic lineages—acting as key effect modifiers.

Monthly invasive pneumococcal disease case counts visibly peak during the colder months, coinciding precisely with seasonal elevations in mean PM concentrations and inversely tracking relative humidity levels. This visual co-variation directly supports the manuscript’s central claims that (i) air pollution is positively associated with IPD risk and humidity exerts a protective effect at higher levels.

The authors also performed a simple lag analysis (Spearman correlations of environmental exposures with disease outcomes) that identified maximum associations up to approximately 5 weeks, providing an empirical basis for the 8-week distributed lag window used in the models.

'Looking at the data' yields clear confirmatory evidence for the direction, seasonality, and urban concentration of the associations.

Moreover, the manuscript has a more nuanced, policy-relevant questions and answer.

Weekly PM at 50 ug (exceeding South Africa’s NAAQS threshold) confers a ~4% cumulative 8-week risk increase (up to 18% at 100 ug)

The authors have also addressed my earlier points on potential confounding by human activity (urban density, indoor crowding, behavioral factors) through explicit discussion of limitations, use of population density as a proxy, and spatiotemporal random effects. They appropriately frame results as observational associations rather than direct causation and provide mechanistic hypotheses grounded in the literature (epithelial adhesion, biofilm formation, serotype-specific virulence factors such as psrP).

I recommend this publication. J

Reviewer #3 (Remarks to the Author):

the abstract does not seem to mention the most novel finding of the paper which is the effects of pollution differ between pneumococcal serotypes / lineages which seems a major oversight.

last three or four sentences of the abstract really could do with some tweaking eg add 'moderate' in front of Humidity (33-49%), the next sentence really needs a bit of fiddling with the last part to make it clearer/ better use of English, and the last sentence I would rewrite completely as I really not sure what it is saying - surely this paper has quantified these relationships? and ~I am not sure their findings will affect vaccine policies, these are too crude to cope with the changes seen here; but this paper is good evidence that pollution is bad and adds to the wealth of data that suggests controlling pollution will be immensely important for public health.

Page 2 'accounting for' phrasing occurs in consecutive sentences

Meterological section in the discussion of temperature it seems cold moderate and high temperatures all increase risk; so what is the comparative temperature with lower risk? this is all bit confusing to the reader and needs a bit more text to make clearer why all three temperature bands have an increased risk

Reviewer #3 (Remarks on code availability):

not an expert so pointless for me to review the code

****

Version 2:

Reviewer comments:

Reviewer #1

(Remarks to the Author)
The authors have addressed the comments appropriately, and I have no further comments.

(Remarks on code availability)

Decision Letter:

Our ref: NMICROBIOL-25124824B

15th June 2026

Dear Dr. Belman,

Thank you for submitting your revised manuscript "Air pollution-driven pneumococcal disease risk is shaped by microbial diversity" (NMICROBIOL-25124824B). It has now been seen by the original referees and their comments are below. The reviewers find that the paper has improved in revision, and therefore we'll be happy in principle to publish it in Nature Microbiology, pending minor revisions to satisfy the referees' final requests and to comply with our editorial and formatting guidelines.

If the current version of your manuscript is in a PDF format, please email us a copy of the file in an editable format (Microsoft Word or LaTex)-- we can not proceed with PDFs at this stage.

We are now performing detailed checks on your paper and will send you a checklist detailing our editorial and formatting requirements in a couple of weeks. Please do not upload the final materials and make any revisions until you receive this additional information from us.

Thank you again for your interest in Nature Microbiology Please do not hesitate to contact me if you have any questions.

Sincerely,

Reviewer #1 (Remarks to the Author):

The authors have addressed the comments appropriately, and I have no further comments.

INTERNAL USE ONLY AIP modules required: ["Life Science"]

Version 3:

Decision Letter:

17th July 2026

Dear Dr Belman,

I am pleased to accept your Article "Pneumococcal population structure influences the effects of air pollution on invasive disease risk in South Africa" for publication in Nature Microbiology. Thank you for having chosen to submit your work to us and many congratulations.

Over the next few weeks, your paper will be copyedited to ensure that it conforms to Nature Microbiology style. We look particularly carefully at the titles of all papers to ensure that they are relatively brief and understandable.

Once your paper is typeset, you will receive an email with a link to choose the appropriate publishing options for your paper and our Author Services team will be in touch regarding any additional information that may be required. Once your paper has been scheduled for online publication, the Nature press office will be in touch to confirm the details.

You may wish to make your media relations office aware of your accepted publication, in case they consider it appropriate to organize some internal or external publicity. Once your paper has been scheduled you will receive an email confirming the publication details. This is normally 3-4 working days in advance of publication. If you need additional notice of the date and time of publication, please let the production team know when you receive the proof of your article to ensure there is sufficient time to coordinate. Further information on our embargo policies can be found here:
https://www.nature.com/authors/policies/embargo.html

After the grant of rights is completed, you will receive a link to your electronic proof via email with a request to make any corrections within 48 hours. If, when you receive your proof, you cannot meet this deadline, please inform us at rjsproduction@springernature.com immediately. You will not receive your proofs until the publishing agreement has been received through our system

Due to the importance of these deadlines, we ask you please us know now whether you will be difficult to contact over the next month. If this is the case, we ask you provide us with the contact information (email, phone and fax) of someone who will be able to check the proofs on your behalf, and who will be available to address any last-minute problems.

Acceptance of your manuscript is conditional on all authors' agreement with our publication policies (see https://www.nature.com/nmicrobiol/editorial-policies). In particular your manuscript must not be published elsewhere.

Authors may need to take specific actions to achieve compliance with funder and institutional open access mandates. If your research is supported by a funder that requires immediate open access (e.g. according to Plan S principles or the NIH public access policy) then you should select the gold OA route, and we will direct you to the compliant route where possible. Because authors warrant under our subscription licensing terms that they haven’t committed to licensing any version of their article under a licence inconsistent with the terms of our agreement – including the applicable embargo period – publication under the subscription model isn’t suitable for authors whose funders require no embargo.

If you have any questions about our publishing options, costs, Open Access requirements, or our legal forms, please contact ASJournals@springernature.com

An online order form for reprints of your paper is available at https://www.nature.com/reprints/author-reprints.html. All co-authors, authors' institutions and authors' funding agencies can order reprints using the form appropriate to their geographical region.

We welcome the submission of potential cover material (including a short caption of around 40 words) related to your manuscript; suggestions should be sent to Nature Microbiology as electronic files (the image should be 300 dpi at 210 x 297 mm in either TIFF or JPEG format). Please note that such pictures should be selected more for their aesthetic appeal than for their scientific content, and that colour images work better than black and white or grayscale images. Please do not try to design a cover with the Nature Microbiology logo etc., and please do not submit composites of images related to your work. I am sure you will understand that we cannot make any promise as to whether any of your suggestions might be selected for the cover of the journal.

You can now use a single sign-on for all your accounts, view the status of all your manuscript submissions and reviews, access usage statistics for your published articles and download a record of your refereeing activity for the Nature journals.

To assist our authors in disseminating their research to the broader community, our SharedIt initiative provides you with a unique shareable link that will allow anyone (with or without a subscription) to read the published article. Recipients of the link with a subscription will also be able to download and print the PDF.

As soon as your article is published, you will receive an automated email with your shareable link.

With kind regards,

P.S. Click on the following link if you would like to recommend Nature Microbiology to your librarian
http://www.nature.com/subscriptions/recommend.html#forms

** Visit the Springer Nature Editorial and Publishing website at www.springernature.com/editorial-and-publishing-jobs for more information about our career opportunities. If you have any questions please click here.**

Open Access This Peer Review File is licensed under a Creative Commons Attribution 4.0 International License, which permits use, sharing, adaptation, distribution and reproduction in any medium or format, as long as you give appropriate credit to the original author(s) and the source, provide a link to the Creative Commons license, and indicate if changes were made.
In cases where reviewers are anonymous, credit should be given to 'Anonymous Referee' and the source.
The images or other third party material in this Peer Review File are included in the article's Creative Commons license, unless indicated otherwise in a credit line to the material. If material is not included in the article's Creative Commons license and your intended use is not permitted by statutory regulation or exceeds the permitted use, you will need to obtain permission directly from the copyright holder.
To view a copy of this license, visit https://creativecommons.org/licenses/by/4.0/

<u>Reviewers Comments and Responses</u>

Reviewer #1 (Remarks to the Author):

The authors have revised and restructured the modelling strategy, which has addressed many core concerns. I have a few remaining suggestions regarding the statistical modelling and technical details.

Query 1.1: The description in the Methods remains brief regarding the DLNM specification for meteorological variables. Please provide a more detailed specification consistent with the GitHub code, cite references for the arglag/argvar choices and conduct the corresponding sensitivity analyses.

Response 1.2: We have expanded the Methods section (Lines 654-669) to provide a comprehensive specification of our DLNM framework. To justify our selection of the exposure-response (argvar) and lag-response (arglag) functions, we conducted a sensitivity analysis comparing our primary models, defined by 3 degrees of freedom (df) for exposure (2 internal knots) and 2 or 3 df for lags, against more flexible alternatives (e.g., 4 df for exposure and 5 df for lags) (Figure S17; Table S6).

While the more flexible exposure models occasionally yielded marginally lower WAIC values, the differences were negligible (typically small WAIC delta < 10). The cumulative risk curves showed that the additional degrees of freedom did not alter the fundamental shape of the association but increased the risk of overfitting at the exposure extremes. Our model specification prioritized model parsimony and stability as is standard in environmental time-series modeling (Gasparrini et al., 2010; Gasparrini et al., 2017).

Lines 654-669

To model the non-linear and delayed effects of environmental factors, we employed natural cubic splines for both the exposure-response (argvar) and lag-response (arglag) functions. We conducted sensitivity analyses to determine the optimal degrees of freedom (df) for these splines (Table S5)(Figure S17). Some of these specification adjustments induced numerical instability in the exposure-response curves. Specifically for absolute humidity, and maximum and minimum temperature the hyper-flexible argvar specification produced spurious oscillations. These oscillations did not have any epidemiologically plausible explanation, and likely reflected overfitting rather than meaningful structure. We ultimately included the more constrained non-linear specification which preserved the overall shape while avoiding artificial fluctuations. The final DLNM crossbasis used a natural cubic spline for the lag-response with 2 degrees of freedom (covering an 8-week lag period), except relative humidity for which 3 df were used to better capture potential non-linear lag structures[95,96]. For each exposure-response function we utilized a natural cubic spline with 3 df, placing internal knots at the 33rd and 67th percentiles. For precipitation two knots were placed at equally spaced intervals on the log-transformed distribution to account for its right skew. These specifications were consistent with established DLNM approaches and maintained model parsimony with sufficient flexibility to capture delayed and non-linear exposure-response associations without overfitting[97,98] (Table S6).

*95. Seok, J., Lee, B. Y. & Yoon, H.-Y. Association between humidity and respiratory health: the 2016–2018 Korea National Health and Nutrition Examination Survey. Respir. Res. **25**, 424 (2024).*

*96. Wu, Z. et al. The lag-effects of meteorological factors and air pollutants on child respiratory diseases in Fuzhou, China. J. Glob. Health **12**, 11010 (2022).*

*97. Gasparrini, A., Armstrong, B. & Kenward, M. G. Distributed lag non-linear models. Stat. Med. **29**, 2224–2234 (2010).*

*98. Gasparrini, A., Scheipl, F., Armstrong, B. & Kenward, M. G. A penalized framework for distributed lag non-linear models. Biometrics **73**, 938–948 (2017).*

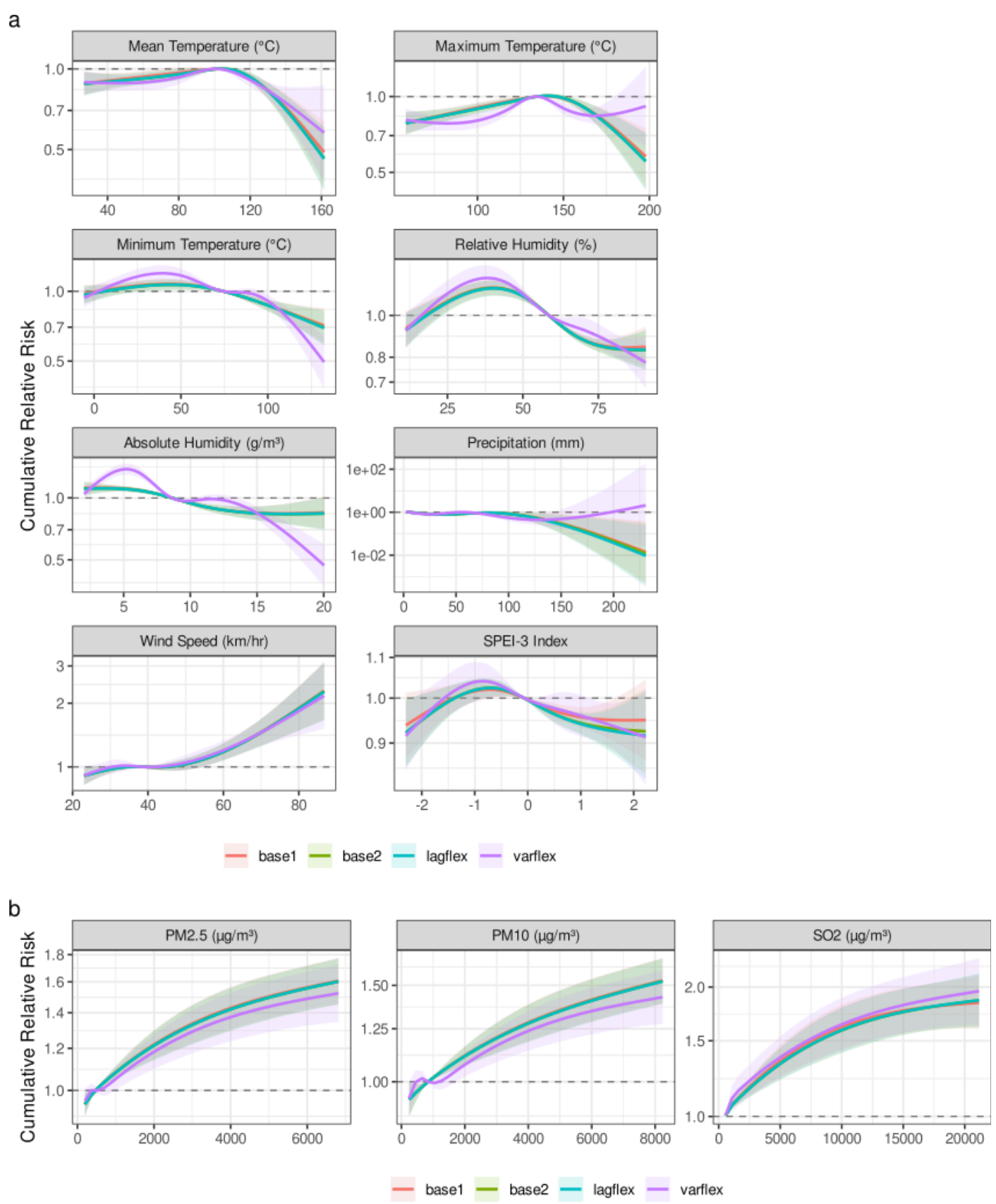

Figure S17. DLNM spline specification sensitivity for (a) meteorological exposures and (b) air pollution exposures with lags up to 8 weeks. Sensitivity analyses across increasing flexibility for the exposure response spline (argvar) and the exposure-lag spline (arglag). kv reflects the number of argvar degrees of freedom while kl reflects the arglag degrees of freedom. Models tested include base1 (var df = 3, lag df =2)(pink), base2 (var df =3, lag df = 3)(green), lagflex (var df =4, lag df = 5)(blue), varflex (var df =4 , lag df = 3)(purple). We include the goodness-of-fit metrics then the WAIC, MAE, -log(cpo), R^2 (Table S5). The shaded areas represent 95% credible intervals.

Query 1.2: Furthermore, I recommend showing the lag-response structure for the main meteorological exposures in the supplementary material to support the selected maximum lag period (e.g. supplementary figures for the lag-response surfaces or marginal lag curves).

Response 1.2: Thank you for this comment, we completely agree that these are informative and added the lag-response surfaces in the previous revision round (Extended Data Fig. 9 and Supplementary Figure 2). We now also include Supplementary Figures S15 and S16 with marginal lag responses at the 25th and

75th percentile for meteorological variables and 10% above the national reference thresholds for the air pollution variables.

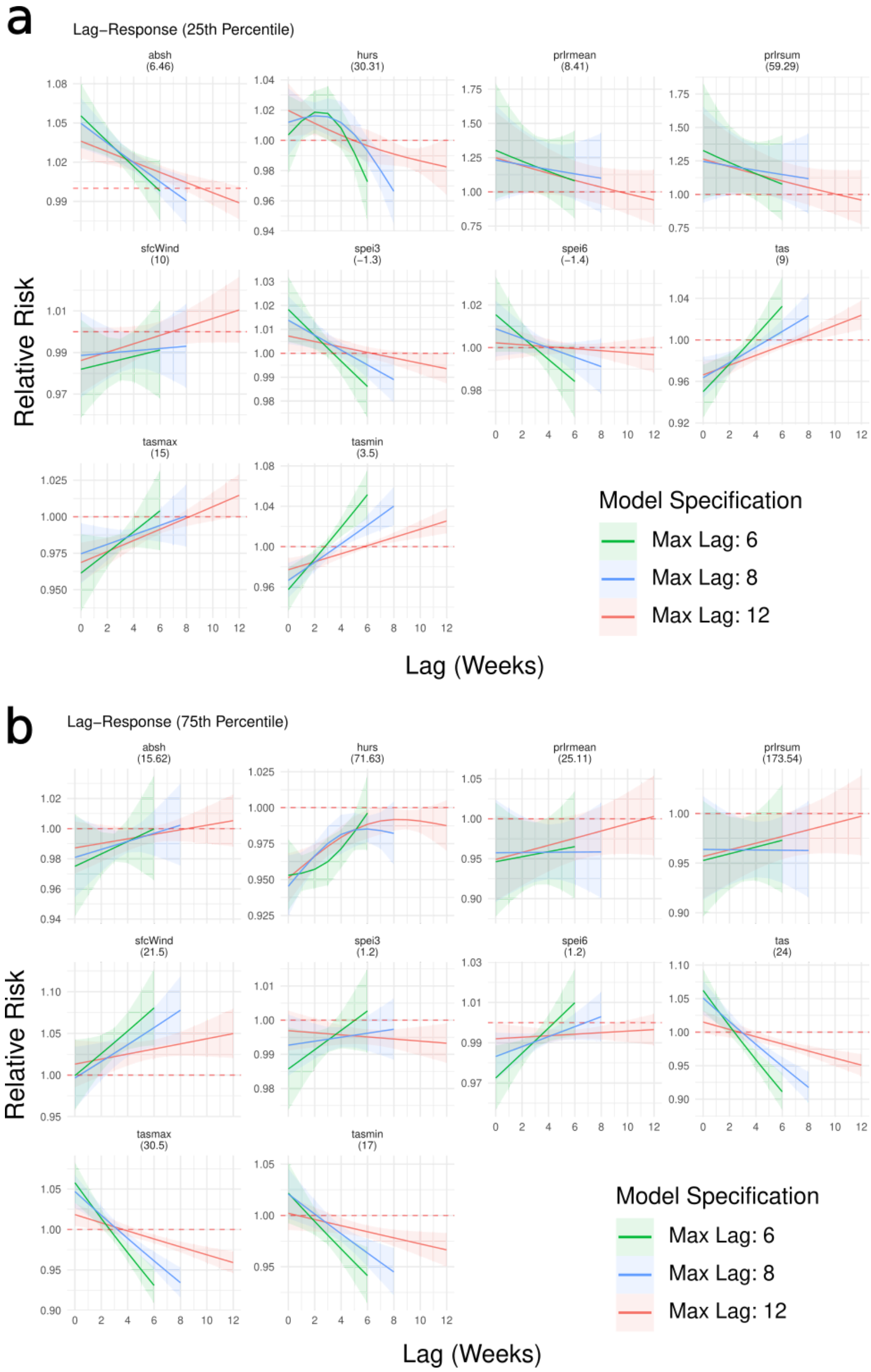

Figure S15. ***Marginal lag response curves*** *at the (a) 25th percentile of the exposure variable and (b) 75th percentile of the exposure variable with maximum lags of 6 (green), 8 (blue), and 12 (pink) weeks for absolute humidity (absh, g/m*^{3}*), relative humidity (hurs, %), mean and total precipitation (prlrmean and prlrsum, millimeters), wind speed (sfcWind, km/hr), standardised precipitation and evapotranspiration index at 3 and 6 months (spei3 and spei6), and mean, maximum, and minimum temperature (tas, tasmax, tasmin; celsius).*

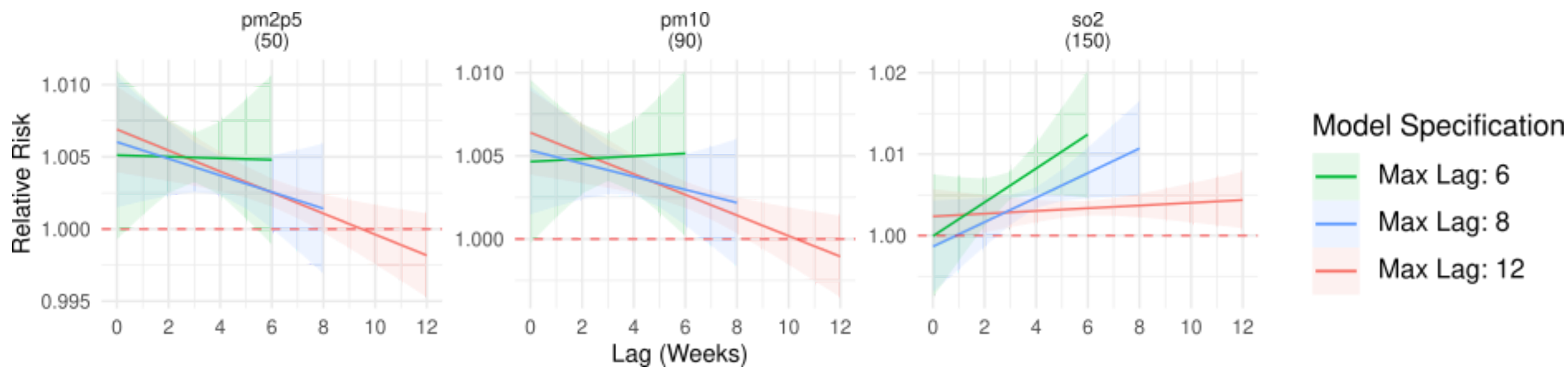

Figure S16. ***Marginal lag response curves for air pollution models*** *at the 10% above the South African National Standard concentration in µg/m^3 for $PM_{2.5}$ (left), PM_{10} (middle), and SO_2 (right) with maximum lags of 6 (green), 8 (blue), and 12 (pink) weeks.*

Query 1.3: The authors mentioned that
"For each variable we included equally distributed knots at the 33rd and 67th quantile except for precipitation for which we included 2 equally spaced knots on a log scale of the precipitation distribution. We centered and scaled all meteorological factors. We centered the effect estimation at the median of the variable distribution (Table S5)." In the GitHub code, I noticed that

```
"if(cov_oi%in%c("pm2p5_lag0","pm10_lag0","o3_lag0","so2_lag0")){
if(cov_oi=="pm2p5_lag0"){ cen = 40 }
if(cov_oi=="pm10_lag0"){ cen = 75 }
# if(cov_oi=="o3_lag0"){ cen = 100 }
if(cov_oi=="so2_lag0"){ cen = 125 }
}";
```

Many air pollution epidemiological studies assume a linear no-threshold concentration–response relationship and therefore use the minimum observed concentration or a very low value as the reference. The rationale for choosing 40, 75, 100 and 125 should be explicitly provided. Similarly, the chosen reference values for temperature and relative humidity should be justified in the manuscript.

Response 1.3: Thank you for this important comment. We intentionally departed from a linear no-threshold-concentration-response relationship and included the air pollution reference values according to the National Ambient Air Quality Standards as set by the South African government to provide a policy relevant interpretation. This allowed us to assess health risks relative to policy compliance and our risk estimates to be in reference to exceedance of these interim standards. This justification is now described in lines 719-724 in the Methods section and Table 6.

Lines 719-724:

"We centered the air quality metrics at the 24-hour National Ambient Air Quality Standards (NAAQS) for South Africa ($PM_{2.5}$ = 40 µg/m^3, PM_{10} = 75 µg/m^3, and SO_2 = 125 µg/m^3). This centering strategy was chosen to align our risk estimates with national regulatory frameworks, providing a direct assessment of health risks associated with standard exceedance rather than an arbitrary minimum value. We utilized the 24 hour exceedance threshold rather than the annual as we are examining an acute IPD effect (Table 6)(Table S6)."

For the meteorological factors we centered the effect estimates at the median so as to estimate the effects under unusual scenarios departing from the median distribution. We have added a sentence describing this in the Methods.

Lines 678-681:

"We centered the effect estimations at the median of each variable's distribution which provided a neutral benchmark to account for a meteorological baseline. Including a center at the median is recognized when a biological optimal exposure point is not defined for a health outcome, as is the case for IPD[83]."

83. Gasparrini, A. Distributed Lag Linear and Non-Linear Models in R: The Package dlnm. *J. Stat. Softw.* **43**, 1–20 (2011).

Query 1.4: In Figure 3a, there are segments of the exposure–response curves at high temperatures where only point estimates appear to be plotted without credible intervals. Could the authors clarify why the intervals are absent for some portions of the curves? Additionally, according to Figure 3a, Additionally, both extremely high and low temperatures appear to show a protective effect against IPD. Is there a plausible biological rationale for this? The authors should briefly discuss this finding.

Response 1.4: Thank you for this comment. We have adjusted the figure appropriately so the entire range of credible intervals is included and accurately labeled. This non-linear cumulative effect masks a varied risk structure between warm and cold temperatures, and is now described in the results section (lines 110-114). We have moved one main figure to the Extended Data so this is now Figure 2.

Lines 110-114:

"Meteorological models showed a non-linear cumulative risk for temperature (Figure 2A), with underlying divergent lag structures: warm temperatures triggered acute risks, whereas cold-related risks were delayed. Only cold temperatures (4-10°C) showed a significant cumulative increase (6°C RR: 1.04; 95% CI 1.01-1.06), though in warmer weeks (31-38°C) we found a 5-11% risk increase relative to the median (Extended Data Fig. 5C; Figure S2)."

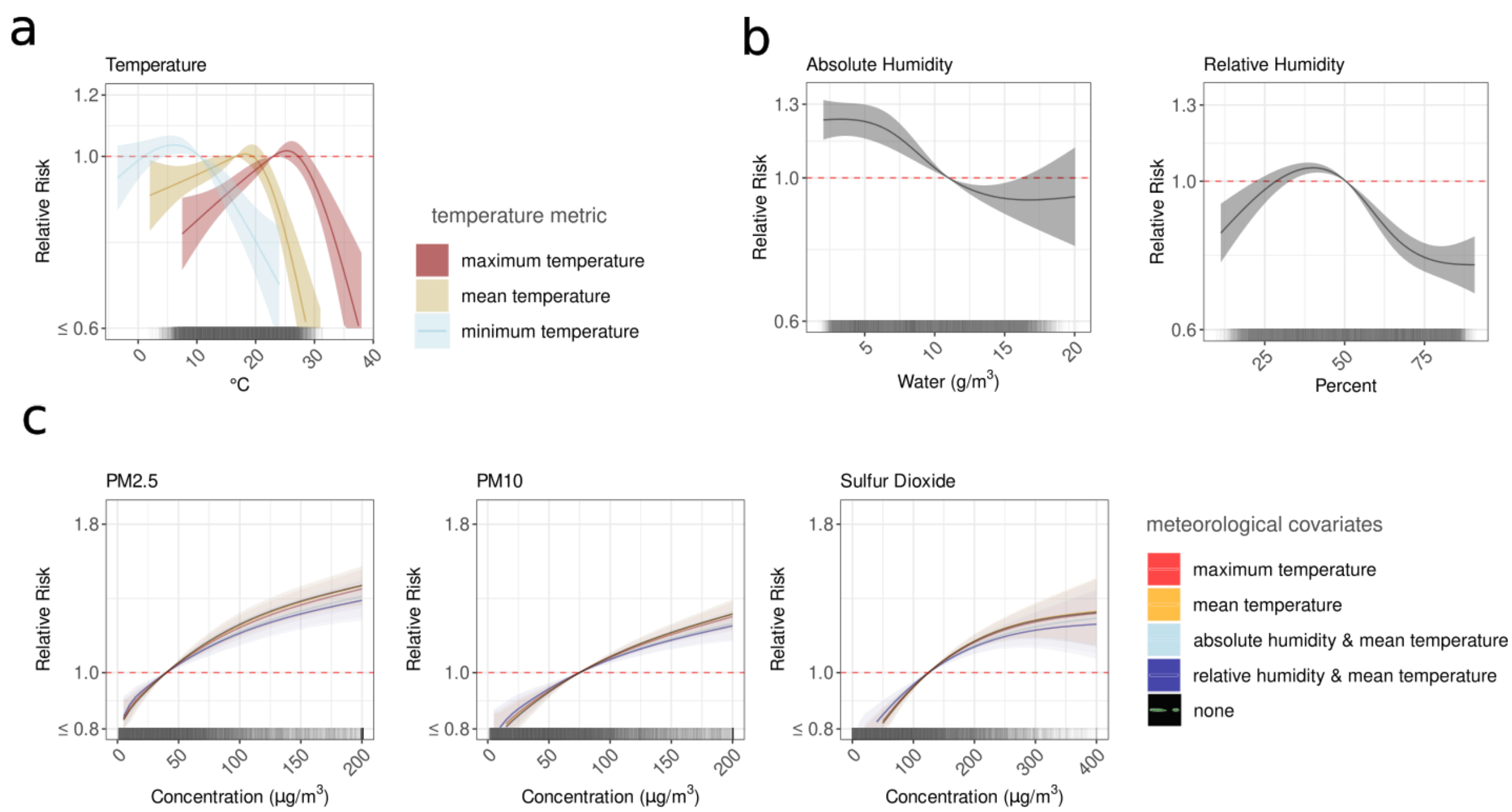

Figure 2. Cumulative relative risk across 8-weeks of lags for (a-b) meteorological models, and (c) air pollution models. *(a) Exposure response curves for maximum (pink), mean (yellow), and minimum (blue) temperature in Celsius (℃). (b) Exposure response curves for absolute humidity (g/m³) (left) and % relative humidity (right)(c) Exposure response curves for $PM_{2.5}$ (left) PM_{10} (middle) or sulfur dioxide (SO_2)(right) including models with combinations of meteorological covariates (temperature metrics with linear formulations and humidity metrics with non-linear formulations): maximum temperature (red), mean temperature (yellow), absolute humidity and mean temperature (light blue), relative humidity and mean temperature (dark blue), and none (black) with three week lags for humidity and no lag for temperature. These models are fit to weekly district level data from 2005-2019 across South Africa. The DLNM crossbasis for the humidity variables has 3-degrees of freedom while the rest of the DLNMs have 2-degrees of freedom. At the bottom of (a-d) is a rug plot with the distribution of the variables in the DLNM. For (a) the rug plot is for mean temperature. The shading represents 95% credible intervals.*

Query 1.5: In Figures 4b, 4c and 4d, the credible interval for the “all disease” group is barely distinguishable. I suggest using a different visual mark to make these intervals visible. Additionally, in Figure 4b, the vertical dashed reference line appears to be drawn at approximately 40 µg/m³ rather than at the 50 µg/m³ stated in the text. Please verify and correct if necessary.

Response 1.5: Thank you for this comment. We now include the “all disease” credible intervals clearly in grey and changed the line to dotted from dashed to make it more distinguishable from the null line (RR=1). We have also shifted the major grid lines so that there is a vertical line at 40 µg/m³. I cannot find a reference to, or any included dashed vertical reference line to which you refer from Figure 4B (now Figure 3B) or elsewhere.

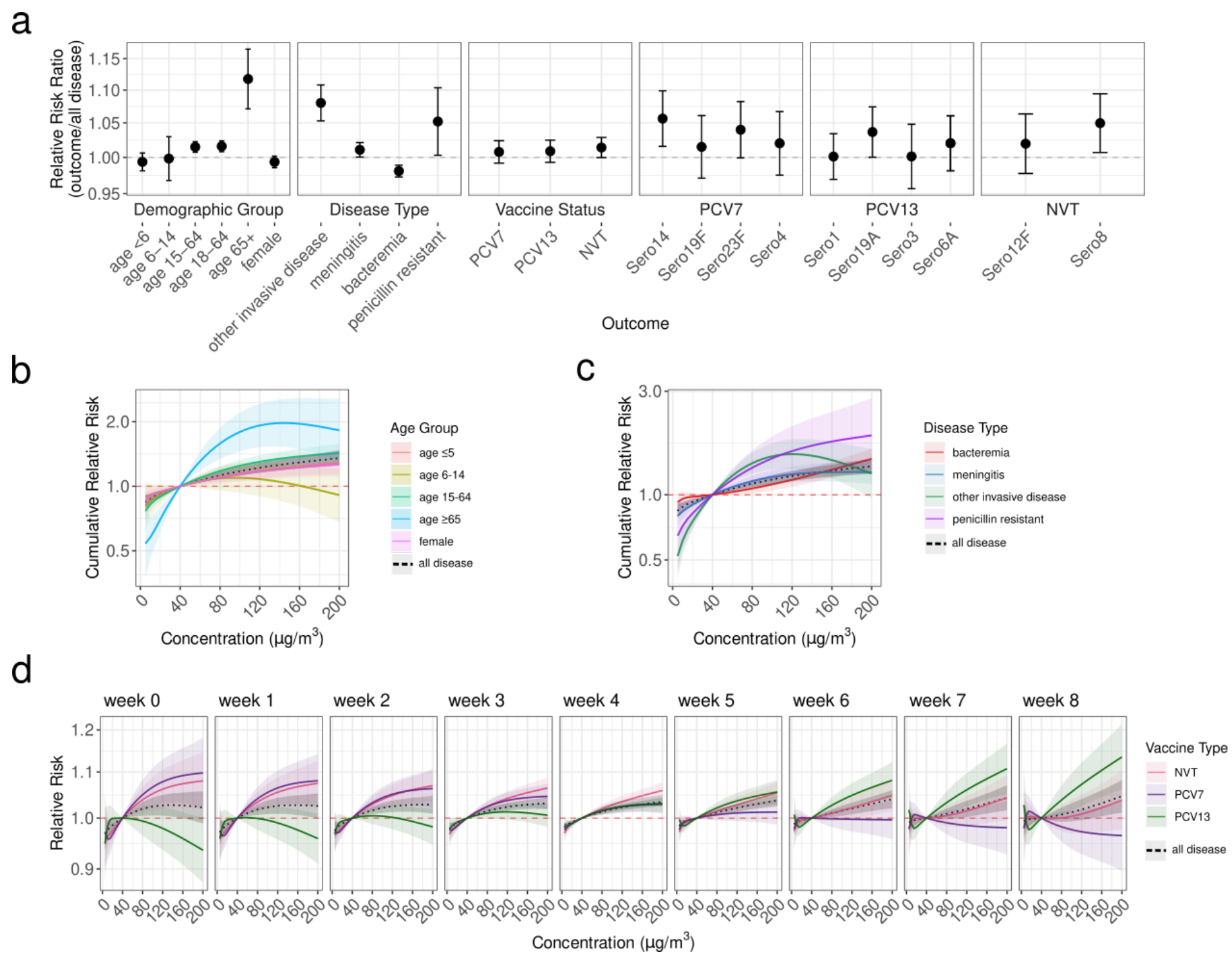

***Figure 3. Risk of specific sub-outcomes of invasive pneumococcal disease given weekly averages of particulate matter <2.5µm ($PM_{2.5}$) in the air pollution model framework**. These models are all fitted to case data at the district level from 2005-2019. (a) relative risk ratio of each specific model compared to the model including all disease at 50 µg/m³ (exceeding the NAAQS threshold for $PM_{2.5}$ by 10 µg/m³) (b) across age groups including age ≤5 (red), age 6-14 (light green), age 15-64 (green), and age 65 and older (blue), and including only females (purple), (c) the relative risk of $PM_{2.5}$ across disease types for bacteremia (red), meningitis (blue), and other invasive disease types (green), penicillin resistant infections (purple) and all disease (black). Weekly lag times for b and c are available in Figure S9. (d) models for disease caused by serotypes targeted by PCV7 (purple), additional 6 serotypes in PCV13 (green), and NVT (red) with a 0 to 8 week lag time from $PM_{2.5}$ to disease for each vaccine type group (pcv7=purple, pcv13=green, nvt=pink). Cumulative vaccine type exposure-response curves are in Figure S10. (b-d) include the RR from the model including all disease cases (black dotted). The shading and error bars represent 95% credible intervals for (a-d).*

Query 1.6: The Methods indicate that "The population size per year per district (from 2005–2023), were sourced from the 2023 Statistics South Africa projections." I was unable to find yearly population estimates at the district level for the full study period in the cited Statistics South Africa Annual Report 2023 (https://www.statssa.gov.za/publications/AnnualReport/AR_Book1202324.pdf). Please double-check the data source and, if necessary, provide a more precise citation or clarify the dataset from which the annual district populations were obtained.

Response 1.6: These estimates were taken directly from the mid-year population estimates for 2023 as supplied in P0302 for statistics South Africa compiled into Excel data tables by the National Institute for Communicable Diseases. These are provided in tables titled "District Estimates 2002-2025" in the most recent version https://www.statssa.gov.za/?PPN=P0302&SCH=74263&page_id=1854. I have improved

the methods section in lines 400-405 to make this clearer and I have adjusted the citation to ensure the URL links to the correct page with these tables.

Lines 400-405:
"Annual district-level population estimates for the period 2005–2023 were obtained from Statistics South Africa mid-year population estimates (Statistical Release P0302). Specifically, we utilized the district municipality population datasets generated from the 2023 projection series, which applies demographic assumptions (births, deaths, and migration) to project forward from the 2022 South African National Census baseline while retrospectively modeling historical annual trends."

Reviewer #2 (Remarks to the Author):

Belman et al. provide compelling evidence that particulate matter and humidity exert non-linear, lagged effects on IPD incidence, with microbial diversity—serotypes and genomic lineages—acting as key effect modifiers.

Monthly invasive pneumococcal disease case counts visibly peak during the colder months, coinciding precisely with seasonal elevations in mean PM concentrations and inversely tracking relative humidity levels. This visual co-variation directly supports the manuscript's central claims that (i) air pollution is positively associated with IPD risk and humidity exerts a protective effect at higher levels.

The authors also performed a simple lag analysis (Spearman correlations of environmental exposures with disease outcomes) that identified maximum associations up to approximately 5 weeks, providing an empirical basis for the 8-week distributed lag window used in the models.

'Looking at the data' yields clear confirmatory evidence for the direction, seasonality, and urban concentration of the associations.

Moreover, the manuscript has a more nuanced, policy-relevant questions and answer.

Weekly PM at 50 ug (exceeding South Africa's NAAQS threshold) confers a ~4% cumulative 8-week risk increase (up to 18% at 100 ug)

The authors have also addressed my earlier points on potential confounding by human activity (urban density, indoor crowding, behavioral factors) through explicit discussion of limitations, use of population density as a proxy, and spatiotemporal random effects. They appropriately frame results as observational associations rather than direct causation and provide mechanistic hypotheses grounded in the literature (epithelial adhesion, biofilm formation, serotype-specific virulence factors such as psrP).

I recommend this publication. J

Response Reviewer #2: Thank you very much for your helpful reviews. They have improved the rigor and clarity of the manuscript overall. We are grateful for your recommendation.

Reviewer #3 (Remarks to the Author):

Query 2.1: the abstract does not seem to mention the most novel finding of the paper which is the effects of pollution differ between pneumococcal serotypes / lineages which seems a major oversight.
last three or four sentences of the abstract really could do with some tweaking eg add 'moderate' in front of Humidity (33-49%), the next sentence really needs a bit of fiddling with the last part to make it clearer/ better use of English, and the last sentence I would rewrite completely as I really not sure what it is saying - surely this paper has quantified these relationships? and ~I am not sure their findings will affect vaccine policies, these are too crude to cope with the changes seen here; but this paper is good evidence that pollution is bad and adds to the wealth of data that suggests controlling pollution will be immensely important for public health.
Response 2.1: Thank you for the attention to the abstract. We have now rewritten this to better reflect the important findings in the manuscript and to clarify the associations. We have also removed the reference to vaccination timing.

"Streptococcus pneumoniae (pneumococcus) is a major global cause of human disease. Despite distinct seasonality the interplay between microbial diversity and environmental drivers remains unexplored. We analyzed 59,017 invasive pneumococcal disease (IPD) cases over 15 years from South Africa, incorporating 4,350 genome-sequenced isolates, using Bayesian spatio-temporal models to link environmental exposures and bacterial diversity. Moderate humidity (33-49%) conferred a 5% increase in IPD risk, while warm temperatures (31-38°C) were associated with acute risks and cold-related risks (4-10°C) were delayed. There was a clear positive association between $PM_{2.5}$ and IPD although it was not uniform; varying by age, disease presentation, and most notably serotype and lineage. Specifically, the lag time between $PM_{2.5}$ exposure and disease onset varied by serotype with only serotypes 4, 8, and 23F conferring an immediate IPD risk. We also found that high prevalence of GPSC21 lineage (serotype 19F) modified the pollution response, shifting the lag structure to produce immediate disease spikes following high $PM_{2.5}$ exposure. By quantifying how microbial diversity modifies environmental risk, we demonstrate that air quality can shape the fitness landscape of microbial populations. These findings suggest that integrating microbial diversity and environmental data may be useful for optimizing public health policy and provides further evidence of the negative health impacts of air pollution."

Query 2.2: Page 2 'accounting for' phrasing occurs in consecutive sentences
Response 2.2: Thank you for this comment. We have adjusted this section and removed the multiple instances of "accounting for" from lines 62-73.

Query 2.3: Meteorological section in the discussion of temperature it seems cold moderate and high temperatures all increase risk; so what is the comparative temperature with lower risk? this is all bit confusing to the reader and needs a bit more text to make clearer why all three temperature bands have an increased risk
Response 2.3: The association is positive across all three bands whereby as temperature increases IPD risk increases. The difference lies in the timing. With colder temperatures the disease risk is delayed risk whereas the lags between warmer temperatures and increased disease risk are much shorter/more immediate. This is shown in Extended Data Fig. 5C and Figure S2 and now discussed clearly in the Results and Discussion.

Lines 110-114:
"Meteorological models showed a non-linear cumulative risk for temperature (Figure 2A), with underlying divergent lag structures: warm temperatures triggered acute risks, whereas cold-related risks were delayed. Only cold temperatures (4-10°C) showed a significant cumulative increase (6°C RR: 1.04; 95% CI 1.01-1.06), though in warmer weeks (31-38°C) we found a 5-11% risk increase relative to the median (Extended Data Fig. 5C; Figure S2)."

Lines 267-268:
"Temperature effects were nuanced: higher temperatures conferred immediate risk while cold effects were delayed, possibly reflecting differing mechanistic relationships with IPD"

Reviewer Expertise:

Referee #1: air pollution, epidemiology, modelling
Referee #2: environmental health and epidemiology, climate change and pollution
Referee #3: Pneumococcal infections, clinical

Reviewer Comments:

Reviewer #1 (Remarks to the Author):

This study is an ecological study using the cases of invasive pneumococcal disease (IPD) in nine provinces of South Africa to investigate the complex relationship between PM2.5, microbial diversity (pneumococcal serotypes and lineages), and IPD based on spatio-temporal Bayesian hierarchical models and distributed lag non-linear models. The findings identified the impact of PM2.5 with variable lag-times of the effects across pneumococcal serotypes, age groups, and disease types, and the greatest increased risk in older adults. Overall, this is a large-scale and innovative study integrating genomic surveillance data and environmental epidemiology framework. However, several major methodological concerns need to be addressed to strengthen the conclusions.

Dear Reviewer #1,
Thank you for your comprehensive review. It highlighted some major limitations in our evaluation of air pollutant effects on IPD. We have re-structured the analysis to separately evaluate the meteorological covariates, and the air pollution covariates which now adjust appropriately for meteorological confounding. We evaluated multiple formulations including meteorological covariates and ultimately included both absolute humidity and mean temperature in all of our air pollution models.

Major Concerns:
Query 1.1: The environmental exposure modeling could be strengthened, particularly through more systematic inclusion of key meteorological variables in the primary model. According to the description in the section of “Base Model Evaluation”, the environmental exposure analysis relies heavily on univariable DLNMs and temperature was not included in the base model as a core exposure variable. Given the biological plausibility of temperature in affecting pathogen survival, host behavior (e.g., indoor crowding), and immune response, its absence from the primary analytical framework is a significant limitation.

The observation that maximum temperature was a top univariate predictor underscores its importance, and considering temperature only in secondary analyses may underestimate its role in the primary effect structure. The meteorological variables, such as temperature and humidity are not only potential confounders but are also strongly correlated with air pollution and may interact with each other to affect human health. However, these variables were not systematically evaluated in the primary model and were mainly explored in univariable or multiplicative models. This modeling strategy may lead to residual confounding in the estimated PM2.5 effects.

Response 1.1: Thank you for highlighting this limitation to our work. We have restructured the manuscript to first include the development of a sociodemographic base model which accounts for spatiotemporal effects as well as interventions (vaccination or antiretroviral therapy) and population density. We then develop our meteorological model which evaluates meteorological effects (temperature, humidity, precipitation, drought indicators, wind speed) using distributed lag non-linear models (DLNM) by including the DLNM crossbasis for each meteorological variable together with the sociodemographic base model. We then develop an air pollution model independently by evaluating the inclusion of multiple different formulations of temperature and humidity metrics together with air pollutants, $PM_{2.5}$, PM_{10}, and SO_2 respectively, and our sociodemographic base model. We finally proceed to evaluate outcome specific and lineage moderated effects from $PM_{2.5}$ utilizing the above developed air pollution model (which adjusts for meteorological confounding). We have updated **Figures 3-5** in the main text to reflect these new models. **Figure S8** demonstrates the changes in relative risk for $PM_{2.5}$ and PM_{10} when accounting for these confounders, **Figure S12** now shows the different surfaces for model formulation comparisons, and **Table 5** demonstrates the goodness-of-fit metrics across meteorological confounder model formulations evaluated. The final air pollution model is described in **Equation 3** and is utilized in all subsequent air pollution analyses to account for meteorological confounding.

Equation 3: $\log(\rho_{s,t}) = \alpha + \beta_{pd}pd_{s,a(t)} + f(pcv_{periods}) + \beta_{temp}temp_{s,t} + f(hum_{s,t\text{-}3}) + CB_{aq}(aq_{s,t-\ell}) + \delta_{m(t)} + \gamma_{pr(s),a(t)} + u_s + v_s$

Table 5. Model comparison for $PM_{2.5}$ including different model formulations for inclusion of meteorological confounders. *These all include the sociodemographic base model and a $PM_{2.5}$ crossbasis ($CB_{aq}(aq_{s,t-\ell})$) as well as a temperature covariate with no lag time ($temp_{s,t}$) or a 3-week lag time ($temp_{s,t\text{-}3}$), and a non-linear formulation of absolute humidity with a 3-week lag time ($f_2(hum_{s,t\text{-}3})$). Additional evaluated formulations are included in Table S5. f indicates a non-linear function.*

Model	Cumulative 8-week RR at 50µg/m³	WAIC	MAE	CPO
$\log(\rho_{s,t}) = \alpha + \beta_{pd}pd_{s,a(t)} + f(pcv_{periods}) + CB_{aq}(aq_{s,t-\ell}) + \delta_{m(t)} + \gamma_{pr(s),a(t)} + u_s + v_s$	1.04 (1.03-1.05)	78684	0.697	0.970
$\log(\rho_{s,t}) = \alpha + \beta_{pd}pd_{s,a(t)} + f(pcv_{periods}) + \beta_{temp}temp_{s,t\text{-}3} + CB_{aq}(aq_{s,t-\ell}) + \delta_{m(t)} + \gamma_{pr(s),a(t)} + u_s + v_s$	1.04 (1.03-1.05)	78686	0.697	0.970
$\log(\rho_{s,t}) = \alpha + \beta_{pd}pd_{s,a(t)} + f(pcv_{periods}) + f(hum_{s,t\text{-}3}) + CB_{aq}(aq_{s,t-\ell}) + CB_{aq}(aq_{s,t-\ell}) \cdot temp_{s,t\text{-}3} + \delta_{m(t)} + \gamma_{pr(s),a(t)} + u_s + v_s +$	1.04 (1.01-1.007)	78662	0.696	0.970
$\log(\rho_{s,t}) = \alpha + \beta_{pd}pd_{s,a(t)} + f(pcv_{periods}) + \beta_{temp}temp_{s,t\text{-}3} + f(hum_{s,t\text{-}3}) + CB_{aq}(aq_{s,t-\ell}) + \delta_{m(t)} + \gamma_{pr(s),a(t)} + u_s + v_s$	1.03 (1.02-1.05)	78661	0.696	0.970
$\log(\rho_{s,t}) = \alpha + \beta_{pd}pd_{s,a(t)} + f(pcv_{periods}) + f(hum_{s,t\text{-}3}) + CB_{aq}(aq_{s,t-\ell}) + CB_{temp}(temp_{s,t-\ell}) + \delta_{m(t)} + \gamma_{pr(s),a(t)} + u_s + v_s$	1.03 (1.02 - 1.05)	78659	0.696	0.970
$\log(\rho_{s,t}) = \alpha + \beta_{pd}pd_{s,a(t)} + f(pcv_{periods}) + f(hum_{s,t\text{-}3}) + CB_{aq}(aq_{s,t-\ell}) + CB_{aq}(aq_{s,t-\ell}) \cdot temp_{s,t} + \delta_{m(t)} + \gamma_{pr(s),a(t)} + u_s + v_s$	1.03 (1.02-1.05)	78658	0.696	0.970

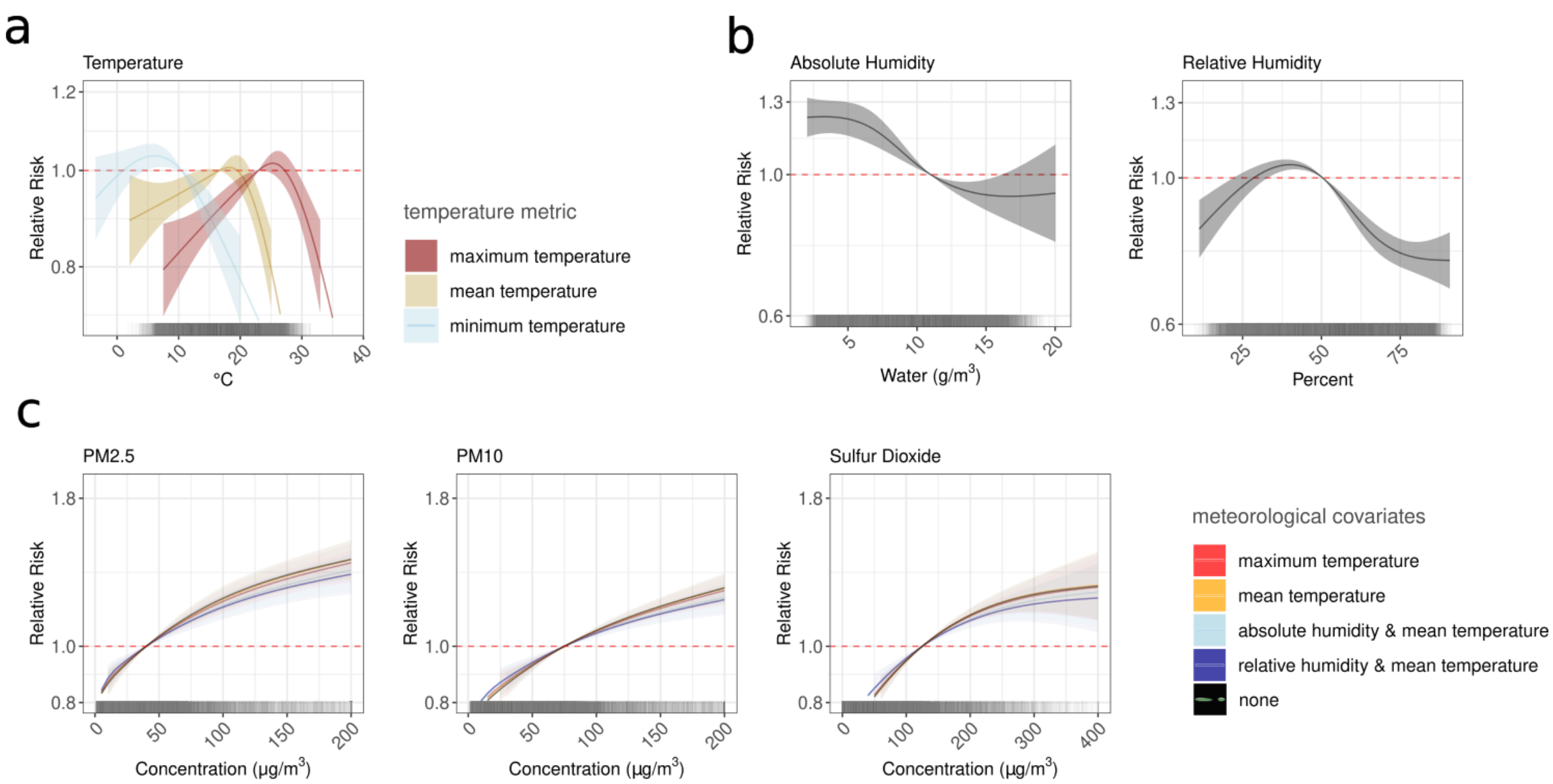

Figure 3. Cumulative relative risk across 8-weeks of lags for (a-b) meteorological models, and (c) air pollution models. *(a) Exposure response curves for maximum (pink), mean (yellow), and minimum (blue) temperature in Celsius (℃). (b) Exposure response curves for absolute humidity (g/m*^{3}*) (left) and % relative humidity (right)(c) Exposure response curves for* $PM_{2.5}$ *(left)* PM_{10} *(middle) or sulfur dioxide (*SO_2*)(right) including models with combinations of meteorological covariates (temperature metrics with linear formulations and humidity metrics with non-linear formulations): maximum temperature (red), mean temperature (yellow), absolute humidity and mean temperature (light blue), relative humidity and mean temperature (dark blue), and none (black) with three week lags for humidity and no lag for temperature. These models are fit to weekly district level data from 2005-2019 across South Africa. The DLNM crossbasis for the humidity variables has 3-degrees of freedom while the rest of the DLNMs have 2-degrees of freedom. At the bottom of (a-d) is a rug plot with the distribution of the variables in the DLNM. For (a) the rug plot is for mean temperature. The shading represents 95% credible intervals.*

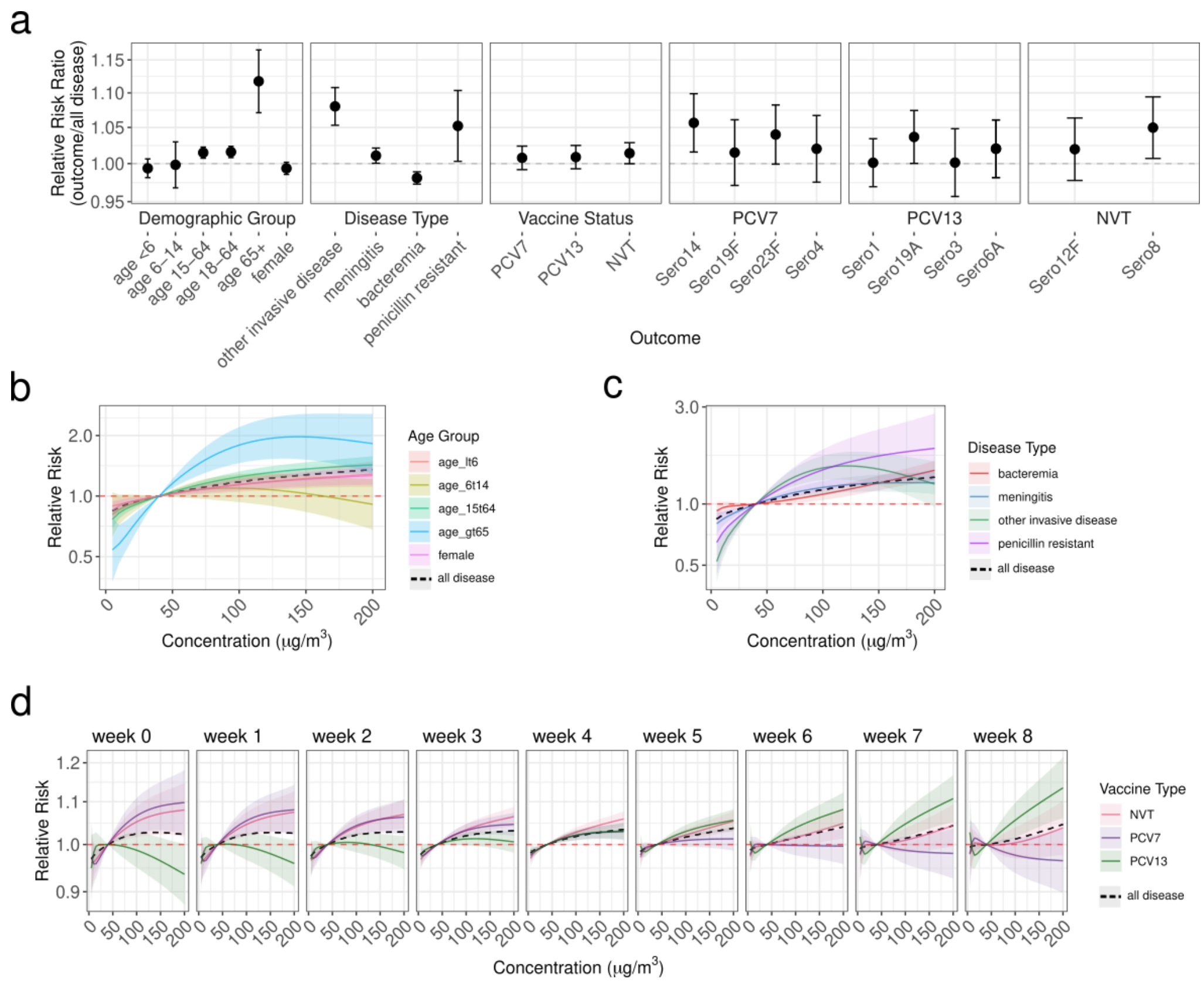

Figure 4. Risk of specific sub-outcomes of invasive pneumococcal disease given weekly averages of particulate matter <2.5μm ($PM_{2.5}$) in the air pollution model framework*. These models are all fitted to case data at the district level from 2005-2019. (a) reporting the relative risk ratio of each specific model compared to the model including all disease at 50 μg/m^3 (exceeding the NAAQS threshold for $PM_{2.5}$ by 10 μg/m^3) (b) across age groups including age ≤5 (red), age 6-14 (light green), age 15-64 (green), and age 65 and older (blue), and including only females (purple), (c) the relative risk of $PM_{2.5}$ across disease types for bacteremia (red), meningitis (blue), and other invasive disease types (green), penicillin resistant infections (purple) and all disease (black). Weekly lag times for b and c are available in Figure S15. (d) models for disease caused by serotypes targeted by PCV7 (purple), additional 6 serotypes in PCV13 (green), and NVT (red) with a 0 to 8 week lag time from $PM_{2.5}$ to disease for each vaccine type group (pcv7=purple, pcv13=green, nvt=pink) Cumulative vaccine type exposure-response curves are in Figure S16. All models also include the RR from a model including all disease cases (black dashed). The shading and error bars represent 95% credible intervals for (a-d).*

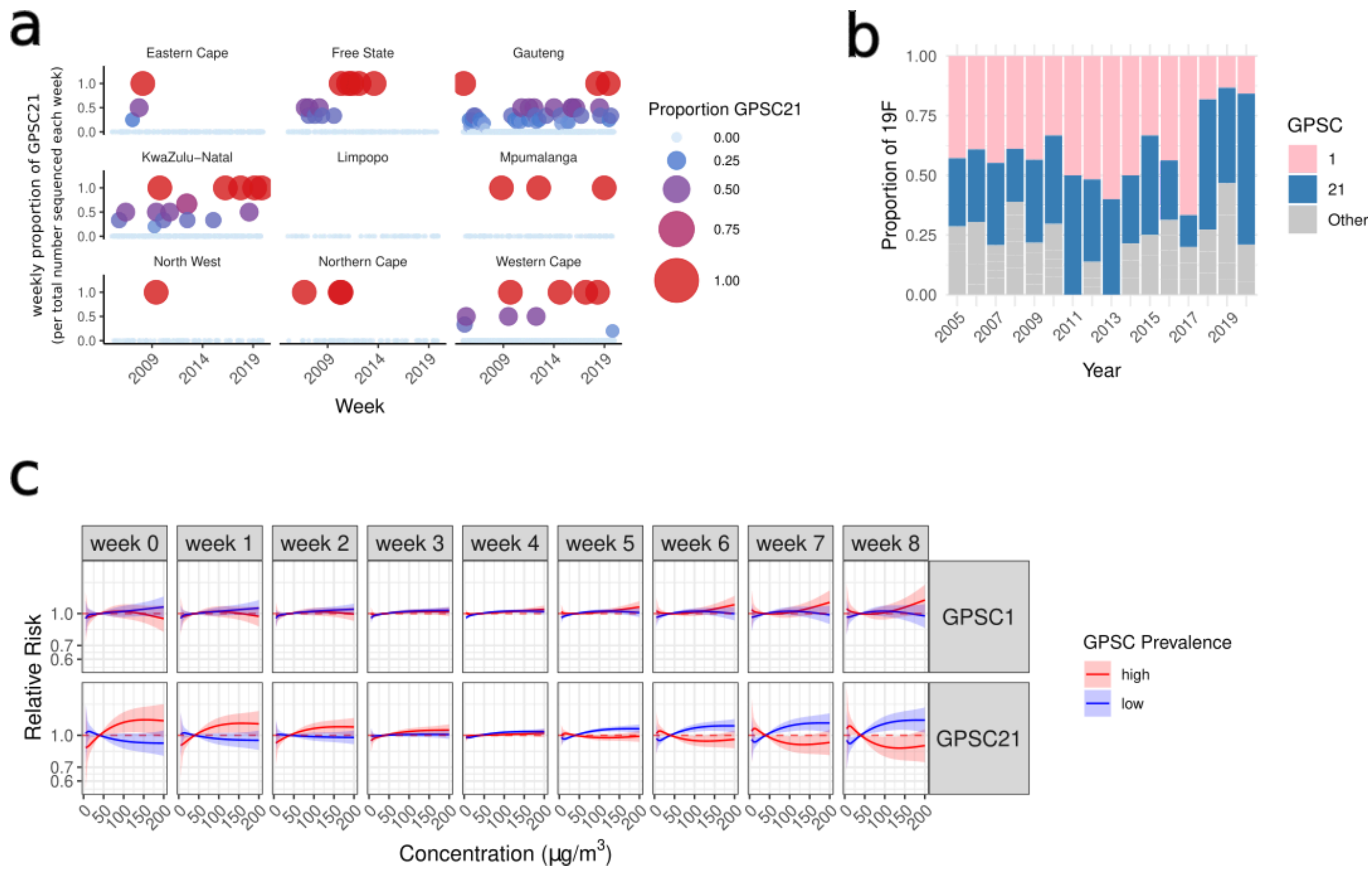

Figure 5. Incorporating GPSC21 by modifying the effect of $PM_{2.5}$ on IPD in the air pollution model framework *fo (a) the proportion of sequences which were GPSC21 in each week and province per the total number sequenced in each week and (b) the proportion of serotype 19F isolates which are found on each GPSC per year highlighting GPSC1 (pink) and GPSC21 (blue) (c) exposure-response curves for the relative risk of province-level IPD across concentrations of $PM_{2.5}$ for GPSC1 (top) and GPSC21 (bottom) including the curves for high prevalence (red) and low prevalence (blue) of each lineage. The shading represents 95% credible intervals.*

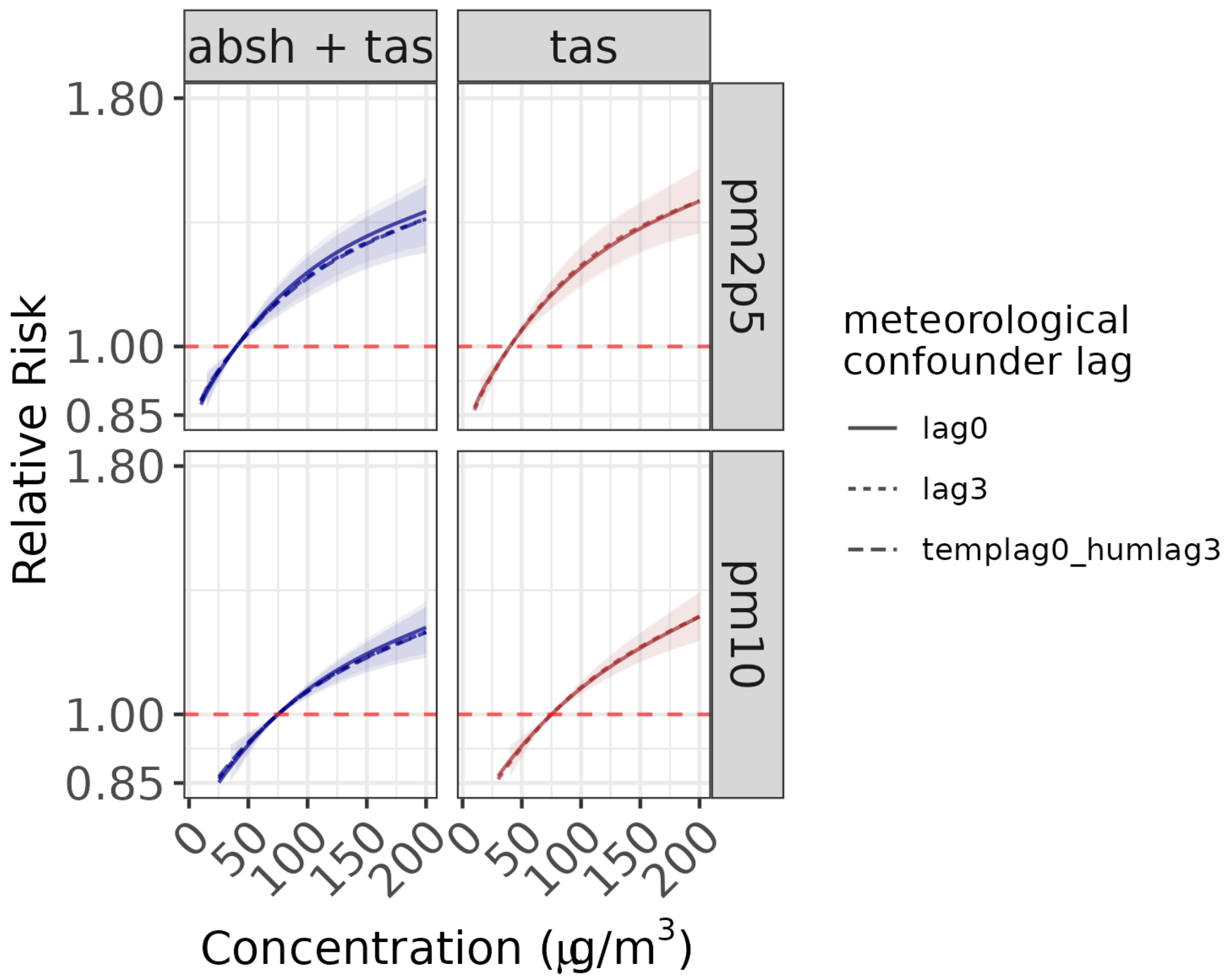

Figure S8. ***Exposure-response curves across 8 weeks of lags for the relative risk*** *(y-axis) from each $PM_{2.5}$ (top) and PM_{10} (bottom) (x-axis) in DLNM models. These models are fit to weekly district level case data with the sociodemographic base model and $PM_{2.5}$ or PM_{10} DLNMs with additional absolute humidity (absh) and mean temperature (tas) incorporating meteorological confounding covariates at different lag times. Lag times incorporated here include mean temperature with no lag time (solid) and a 3-week lag (dotted) time and absolute humidity with temperature both with no lag time, both at a 3-week lag time, and with humidity at a 3-week lag time and mean temperature with no lag time (dashed). The best model formulation is the one with the mixture of humidity at 3 weeks and temperature with no lag.*

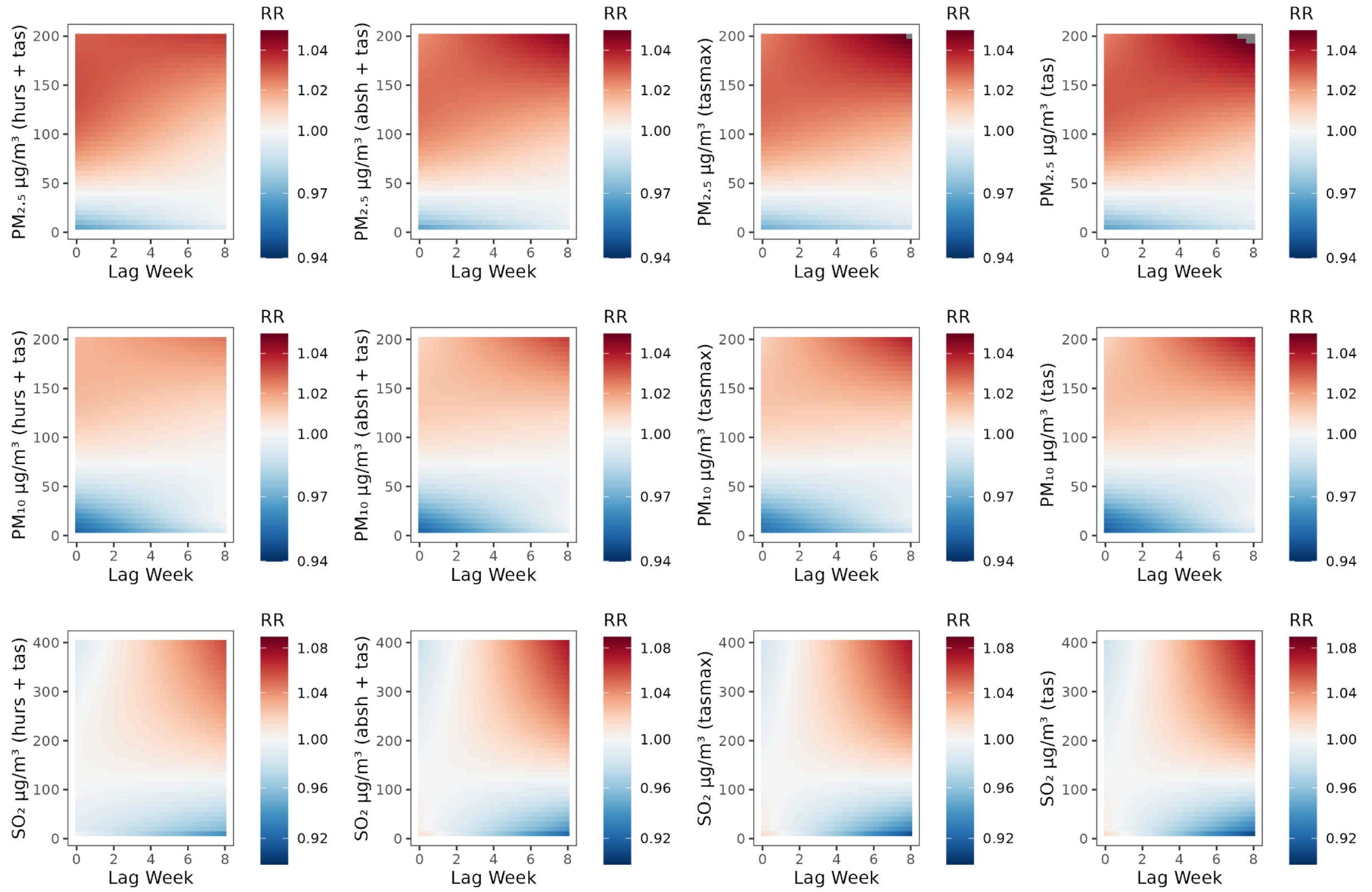

***Figure S12. Mean relative risk (RR) surfaces from the air pollution models** where risk >1 is red and <1 is blue for $PM_{2.5}$ (top row), PM_{10} (middle row), and SO_2 (bottom row) including adjustment for different formulations of meteorological confounders from left to right for (i) relative humidity and temperature, (ii) absolute humidity and temperature, (iii) maximum temperature, and (iv) mean temperature. The humidity variables are included as non-linear at lag week 3 and the temperature variables are linear at lag week 0. The main air pollution model includes an adjustment for absolute humidity and mean temperature.*

Query 1.2.1 Though the spatial random effects are included, the model may not fully account for fine-scale spatiotemporal confounding. Key environmental variables like PM2.5, humidity, and temperature are often spatiotemporally correlated. The use of province-level replication for interannual effects may mask district-specific exposure-response relationships driven by these correlated factors.

Response 1.2.1

Thank you for raising this important point regarding potential fine-scale spatiotemporal confounding due to spatiotemporally correlated environmental exposures (e.g., $PM_{2.5}$, temperature, and humidity). We agree that such correlations can complicate the identification of exposure-response relationships, particularly when spatial and temporal structures are modeled separately.

In our main specification (the sociodemographic base model), we model spatial variation as time-invariant (district-level random effects) **(Figure S13A)(below)** while allowing interannual variability through province-level replication **(Figure S13B)(Equation 3)**. This formulation was chosen to balance parsimony, identifiability, and interpretability. With a province level interannual replication we

could capture broad region-specific temporal variability (e.g. climate extremes, policy changes) while allowing us to isolate effects of environmental factors at finer scales. Our study period aligned with the rollout of vaccines (PCV) and antiretroviral therapy (ART) which are reported at the provincial level. However, we do acknowledge that having a spatially varying temporal effect at the province level rather than a temporally varying spatial effect at the district level may not fully capture finer-scale spatiotemporal interactions. To explore this, we conducted a sensitivity analysis using the more flexible specification in which spatial random effects were replicated by year (allowing the spatial structure to vary over time) to compare against the interannual effect replicated by province where *f* indicates a non-linear function **(Figure S13C) (Equation 4)**.

Equation 3: $\log(\rho_{s,t}) = \alpha + \beta_{pd}pd_{s,a(t)} + f(pcv_{periods}) + \beta_{temp}temp_{s,t} + f(hum_{s,t\text{-}3}) + CB_{aq}(aq_{s,t-\ell}) + \delta_{m(t)} + \gamma_{pr(s),a(t)} + u_s + v_s$

Equation 4: $\log(\rho_{s,t}) = \alpha + \beta_{pd}pd_{s,a(t)} + f(pcv_{periods}) + \beta_{temp}temp_{s,t} + f(hum_{s,t\text{-}3}) + CB_{aq}(aq_{s,t-\ell}) + \delta_{m(t)} + u_{s,a(t)} + v_{s,a(t)}$

This alternative formulation relaxes the assumption of temporal stability in spatial patterns within provinces and allows for unobserved district-level heterogeneity that evolves each year, potentially capturing residual confounding from spatiotemporally correlated environmental variables at a finer spatial scale. We find clear yearly differences for the spatial effects between the models and for some districts there are residual time varying spatial effects which are not captured in the less flexible formulation.

This increased flexibility comes at the cost of substantially increased model complexity and reduced interpretability. While the time-varying spatial model introduces additional flexibility, it does not materially change the substantive conclusions; rather, it redistributes variability in ways that may be difficult to interpret epidemiologically. In particular:

- Allowing fully time-varying spatial effects introduces a large number of additional parameters, increasing the risk of overfitting, especially given the strong spatiotemporal correlation in environmental exposures, and increases computational instability.
- The differences between the two model specifications are highly correlated with the province-level interannual effect, suggesting that both models capture a similar underlying temporal signal but allocate it differently across spatial and temporal components **(Figure S13B–C)**.
- The estimated exposure-response relationship for $PM_{2.5}$ is virtually unchanged between the two specifications **(Figure S13D)**, both in magnitude and shape.

Overall, while we acknowledge that no model can fully eliminate spatiotemporal confounding, our sensitivity analysis demonstrates that:

1. The main findings are robust to a more flexible spatiotemporal specification, and
2. The chosen model provides a more parsimonious and interpretable framework for addressing the study objectives without evidence of substantial residual confounding.

We have now clarified this rationale in the Methods and included the additional sensitivity analysis in the Supplement **(Figure S13).**

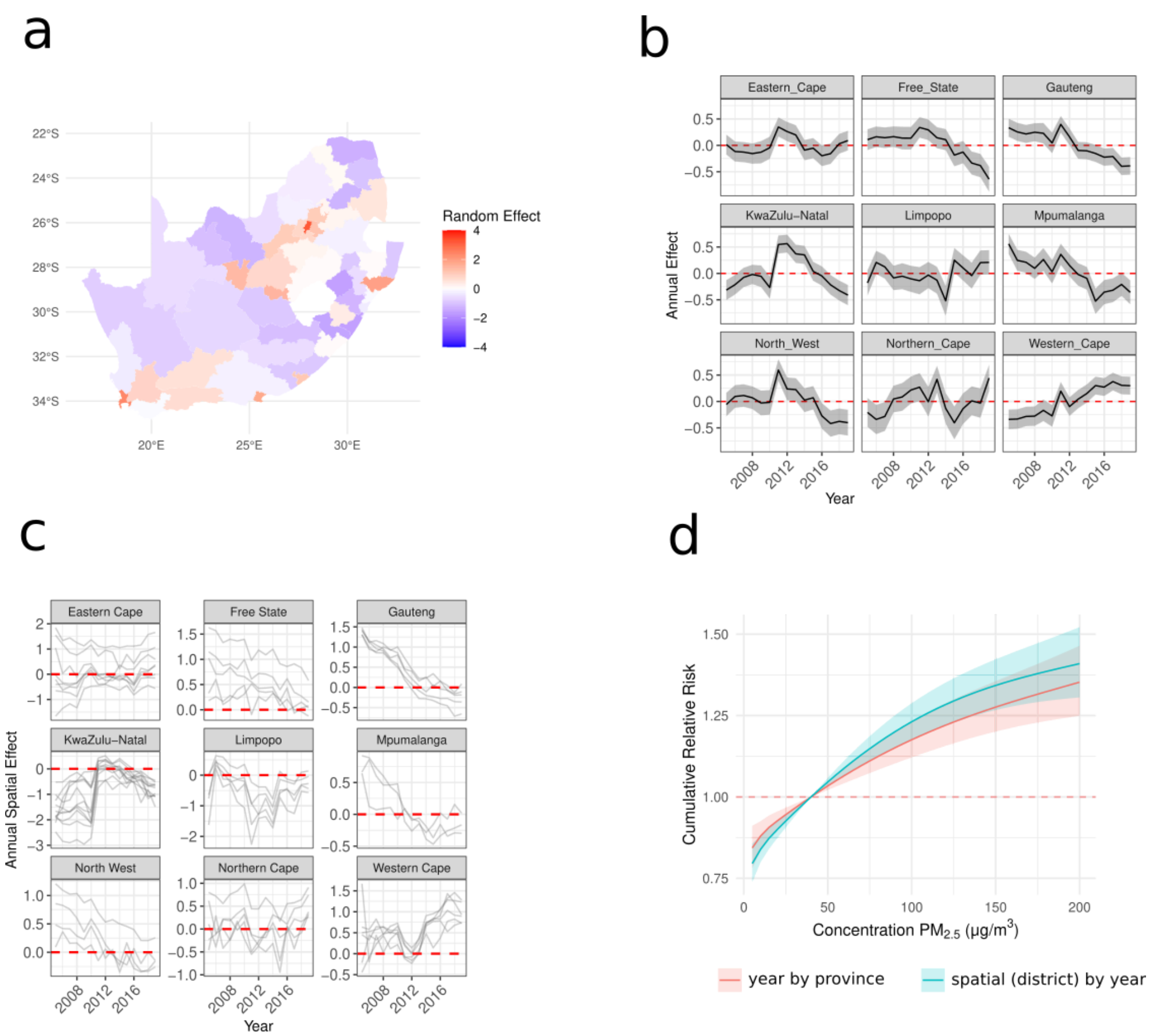

Figure S13. *Model sensitivity with time varying spatial effects. (a) static spatial random effect from equation 1. (b) interannual effect replicated by province from equation 1. (c) spatial effect replicated by year for each district from equation 2. (d) Cumulative exposure-response curve across 8-weeks with the relative risk (y-axis) across weekly μg/m³ of $PM_{2.5}$ (x-axis) comparing the air pollutant model from the main text (pink) (Equation 3) to a model which varies the spatial variation by year (blue) (Equation 4).*

Lines 271-275 (tracked changes version):

"We also explored the potential for fine scale spatiotemporal confounding of the $PM_{2.5}$ effect by allowing spatial effects to vary by year. This showed high correlation with the interannual province level effects from the sociodemographic base model, with a few urban districts as outliers. However, the $PM_{2.5}$ exposure-response relationship was robust to this analysis indicating that fine-scale spatiotemporal confounding was not influential (Figure S13)."

Query 1.2.2 In addition, environmental variable selection is primarily driven by WAIC-based model fit, with limited incorporation of prior epidemiological knowledge. A more theory-informed modeling strategy would improve interpretability.

Response 1.2.2 We agree that interpretability relies on a theory-driven framework. We have restructured the methods section to clarify that our model formulations were driven by epidemiological knowledge of IPD and of the interactions between air pollutants and meteorological factors. We describe our methodological logic below and in lines 740-774 in the document with tracked changes:

1. We first developed a sociodemographic base model which accounted for spatiotemporal random effects as well as other sociodemographic covariates such as vaccination coverage and population density. Population density is a known confounder with air pollution and disease and we wanted to account for it explicitly. Pneumococcal vaccination and antiretroviral therapy in this setting have had a substantial impact on IPD and needed to be explicitly accounted for rather than relying on the residual interannual effect.
2. We then introduced meteorological factors to the sociodemographic base model using distributed lag non-linear models (DLNMs) to understand the non-linear or lagged effect of these factors on IPD.
3. To isolate the independent effects of air pollutants, we modeled air pollution exposures using DLNMs and evaluated them alongside previously identified sociodemographic and meteorological drivers. Because temperature and humidity strongly confound both IPD dynamics and atmospheric chemistry of pollutants, we compared multiple specifications for these variables, including linear, nonlinear, interaction with DLNMs across different lag-times; and with an additional DLNM for temperature (Table 5). While goodness-of-fit and parsimony helped us select the optimal functional form, the inclusion of these variables was theory driven. All air pollution effects reported in the revised manuscript now adjust for meteorological and sociodemographic confounding.

Lines 740-774 (tracked changes version):

"In brief, here we summarise our modeling strategy for identifying meteorological and air pollution factors that drive patterns of pneumococcal disease; with a deeper analysis of the effects of $PM_{2.5}$ on different outcomes (e.g. serotype, age group, disease type).

To assess the risk from environmental factors on IPD we adopted the following modeling strategy:

1. *We first developed a sociodemographic base model which accounted for population density and vaccination period as well as residual seasonal, spatial, and interannual effects.*
2. *We tested different metrics for vaccination and antiretroviral therapy to assess their impact on disease patterns. We chose a sociodemographic base model given a balance between model fit and tractable complexity ultimately including confounding effects of vaccination as a categorical variable (across 3 vaccination periods), and population density as a continuous variable, alongside the residual random effects.*
3. *We then developed a meteorological model that utilized delayed lag non-linear models (DLNMs) to assess the risk from weekly, district level, meteorological factors ($m_{s,t}$) across lag periods. These included absolute humidity, relative humidity, mean, minimum, and maximum temperature, precipitation, wind speed, and the Standard Precipitation Evapotranspiration Index (SPEI; a drought indicator). We evaluated model fit and effect size across 8-weeks of lag time from exposure.*
4. *To elucidate the risk from air pollution we developed an air pollution model that included a DLNM crossbasis for $PM_{2.5}$, PM_{10}, and SO_2 including the same sociodemographic base model and meteorological confounding factors. We tested different formulations for inclusion of the*

meteorological confounding including linear and non-linear covariates of humidity and temperature with different lag-times. We also evaluated the inclusion of a temperature crossbasis alongside the $PM_{2.5}$ crossbasis, and temperature as a modifying interaction with the $PM_{2.5}$ crossbasis. We found the WAIC to be similar across the temperature covariate model, the temperature crossbasis model, and the interaction model. The $PM_{2.5}$ effect sizes were also unchanged across these model types. The final air pollution model included a non-linear lag 3 humidity covariate and linear lag 0 covariate for temperature.

5. *Using our final air pollution model, we then focused on $PM_{2.5}$ to determine how the risk from $PM_{2.5}$ varies given different outcomes (e.g. serotype, age group, disease type).*

Finally we incorporated a modification by different underlying genomic lineages with $PM_{2.5}$ in the same model so as to elucidate how the underlying microbial population modifies these $PM_{2.5}$ effects. The $PM_{2.5}$ air pollution models all accounted for the potentially confounding effects of temperature, humidity, population density, and vaccination."

Query 1.3. Some potentially important confounders or effect modifiers, including indoor living conditions, such as household crowding and indoor airborne pathogen load, are important determinants of IPD. These factors were not considered in the present study and may contribute to residual confounding in the observed exposure-outcome associations. The authors need to evaluate the potential impact of these factors.

Response 1.3 Thank you for this point, we agree that factors such as household crowding are significant determinants of IPD transmission. While primary data on household crowding and indoor air quality were not available in this study we addressed these potential confounders through our modeling strategy: (1) we included yearly population density as a proxy for urban crowding and housing types at the district level (2) we utilized spatial structured and unstructured random effects to capture unmeasured residual variation at the district level. This term accounts for localized clusters of risk at the district level which may be driven by socioeconomic factors not explicitly captured by population data. We acknowledge that sub-district variations remain unobserved. To address your second point, indoor and outdoor air pollution are often highly correlated, particularly in settings where ambient air infiltrates the home, and ambient levels are a meaningful surrogate for exposure. We now include a sentence in the limitations section discussing unmeasured indoor factors that could influence our findings in lines 572-578 and additional citations (57-59), which discusses household $PM_{2.5}$ exposure in South Africa including the correlation with ambient air pollution and the gender dimension.

Lines 572-578 (tracked changes version):

"A limitation of this study is the lack of granular data on indoor air pollution exposure. However, given that indoor and ambient pollutants are often closely correlated, our results likely capture the core exposure-outcome association, though excludes the precise contribution of indoor air pollution[57,58]. We also lacked primary data on indoor household factors, but include population density and spatiotemporal random effects as proxies for some of this unobserved risk. Our inability to account for sub-district level variations in household factors is a further limitation, especially given the known differences in household air pollution exposure when stratified by sex[59]."

57. González-Martín, J., Kraakman, N. J. R., Pérez, C., Lebrero, R. & Muñoz, R. A state–of–the-art review on indoor air pollution and strategies for indoor air pollution control. *Chemosphere* **262**, 128376 (2021).

58. Benyon, M. *et al.* Household PM2.5 in a South African urban and rural setting: A comparative analysis using low-cost sensors. *Atmospheric Pollut. Res.* **16**, 102459 (2025).

59. Somboonsin, P. P. & Baffour, B. Spatial Inequities in Women's Health: The Global Impact of Household Air Pollution from Solid Fuels. *Water. Air. Soil Pollut.* **236**, 934 (2025).

Query 1.4 The section titled "Timing of air quality effects is mediated by lineage diversity" appears to overstate the causal role of lineage diversity, which was modeled as an effect modifier rather than an effect mediator. As described in the methods, "By incorporating the genomic lineages as a modifying proportional interaction effect" suggests the consideration of effect modification rather than effect mediation. Moreover, lineage diversity is only observed among confirmed cases and reflects pathogen characteristics post-infection, limiting its interpretability as a mediator in the causal pathway between air pollution and IPD incidence. The authors may wish to reconsider the terminology or clarify the intended causal interpretation.

Response 1.4 : Thank you for highlighting this imprecise use of terminology. We agree that lineage diversity was modeled as an effect modifier rather than a mediator. Accordingly, we have removed the term mediator throughout the manuscript to clarify that lineage diversity was incorporated as an interaction term with the air pollution exposure in the DLNM framework. This also addresses the causal interpretation as lineage diversity is only measured among confirmed cases and cannot plausibly lie on the causal pathway between air pollution exposure and IPD. Instead it is modifying the association between air pollution and disease risk.

Specific Points for Clarification:

Query 1.5. Figure 1 suggests that stronger correlations between PM2.5 and IPD incidence are predominantly observed before approximately 2013, which coincided with major transitions in pneumococcal conjugate vaccine implementation and serotype replacement. Given the pronounced shifts from PCV7 to PCV13 and subsequently to non-vaccine serotypes, it is unclear whether the exposure-response relationship is stable over time. However, the vaccine implementation or calendar period was not explicitly modeled as an effect modifier in the main analyses. Additional interaction analyses for vaccine implementation would help assess the robustness of the findings.

Response 1.5 Thank you for this point, we have conducted sensitivity analysis for the pre-PCV7 (2005-2009) and post-PCV7 time period to evaluate whether the effects are robust to both time periods (**Figure S16**). We find that the effects are pronounced across all disease types as well as when stratified by non-vaccine type serotypes, PCV7 serotypes, and PCV13 serotypes in both periods. Furthermore when we evaluate the correlation between $PM_{2.5}$ and IPD across the different time periods we find that the correlations remained positive and of comparable magnitude (r = 0.49 for 2005-2009; r = 0.39 for 2009-2020; r = 0.39 for 2005-2020). Together this suggests that the relationship between $PM_{2.5}$ exposure and IPD incidence is not driven solely by a specific vaccine implementation period.

Query 1.6. Line 138, the selection of "an 8-week time period" (Line 138) for the DLNM lag window was stated without strong justification. While plausible for chronic effects, the biological rationale for pneumococcal infection (from exposure to invasive disease) spanning up to 8 weeks was not clearly articulated. Moreover, other important confounders should be included in the model when exploring the lagged structure.

Response 1.6 We include an 8-week time period for exposure to effect for multiple reasons. Previous epidemiological evidence indicated a 1-month lag-time from environmental exposure to changes in pneumococcal dynamics. These include an association at 1 month between environmental factors and IPD in New Zealand (Murdoch & Jennings 2009), and assessment of temperature and air pollution with a significant 4-week lag time to pneumococcal disease effects (Kim et al., 1996). We additionally evaluated models with 6 and 12 weeks finding that 8-weeks captured all effects and did not change the cumulative effects size, while up to 6 weeks the effect was still pronounced by week 6 and by 12 weeks the effect had reversed for most variables. The Spearman's correlation of lag-times from environmental exposure and disease in this dataset found the maximum correlation up to about 5 weeks for all variables (**Figure S3**). Furthermore, the carriage duration and estimated generation time (time from being infected to infecting someone else) for the pneumococcus is estimated at around 4 weeks (Belman et al., 2024). Given the statistical evidence together with the estimated 30 day generation time for the pneumococcus, 8-weeks should capture both the statistical and biologically relevant effects. We have included this logic in the "Association between environmental factors and pneumococcal disease risk" section (Lines 194-198) as well as in the methods (Lines 876-882).

Lines 194-198 (tracked changes version):

*"*We included an 8-week lag period due to a combination of previous epidemiological evidence of up to a 1-month lag time from environmental exposure to pneumococcal disease effects, the maximum correlation from environmental exposure to disease up to about a 5-week lag time, and previous evidence of a 30 day estimated generation time for the pneumococcus (Figure S3)[6,18–20]*."*

Lines 876-881 (tracked changes version):

"We proceeded with models including lags of up to 8 weeks from environmental exposure to pneumococcal disease due to our own model comparison and previous work identifying effects up to 4 weeks after environmental exposure[18,19]. Furthermore we found the maximum correlation between the disease outcome and environmental exposure up to around a 5-week (Figure S3). Given the statistical evidence together with the estimated 30 day generation time for the pneumococcus,an 8-week lag period is expected to capture both the statistically and biologically relevant effects[6]."

6. Belman, S. *et al.* Geographical migration and fitness dynamics of Streptococcus pneumoniae. *Nature* **631**, 386–392 (2024).
18. Kim, P. E. *et al.* Association of Invasive Pneumococcal Disease with Season, Atmospheric Conditions, Air Pollution, and the Isolation of Respiratory Viruses. *Clin. Infect. Dis.* **22**, 100–106 (1996).
19. Murdoch D.R. & Jennings L.C. Association of respiratory virus activity and environmental factors with the incidence of invasive pneumococcal disease. *J. Infect.* **58**, 37 EP – 46 (2009).
20. Beentjes, D., Shears, R. K., French, N., Neill, D. R. & Kadioglu, A. Mechanistic Insights into the Impact of Air Pollution on Pneumococcal Pathogenesis and Transmission. *Am. J. Respir. Crit. Care Med.* **206**, 1070–1080 (2022).

Query 1.7. Line 144, the authors observed that "Humidity and precipitation were negatively associated with IPD risk". It is recommended to provide sufficient supporting evidence. Notably, in the South

African context, extreme rainfall events, floods, or tropical cyclones may also have profound but complex impacts on infectious disease dynamics, through displacement, water contamination, and disruption of health services. The authors should consider the potential impact of extreme rainfall events and flooding.

Response 1.7 Thank you for this comment. The observed negative associations between humidity, precipitation, and IPD risk are consistent with prior literature suggesting that drier conditions may facilitate pneumococcal transmission and/or host susceptibility. However, there is also conflicting evidence in arid regions (Jusot et. al., 2017 in Nigeria; Ciruela et al., 2016 in Catalunya) which find positive associations with these effects. We have edited this entire section to make the results clearer and better aligned with our methods. The title of this section is now:

"Associations between environmental factors and pneumococcal disease risk"

The relevant sections to this reviewer comment are copied here:

Lines 216-252 (tracked changes version):
"Our meteorological models comprise the sociodemographic base model plus a DLNM for each variable. Temperature had a non-linear cumulative association with IPD across the time period with only average minimum weekly temperatures of 4–10°C conferring an increased risk of IPD (6°C RR 1.04: 95% CI 1.01-1.06) (Figure 3A). Weeks with a mean daily maximum temperature of 31°C were associated with a 5% increased risk of IPD relative to the median, rising to an 11% increased risk at 38°C. Similarly, mean weekly temperatures of 24°C were associated with a 5% risk increase (RR 1.05: 95% CI 1.03-1.08). In contrast, weekly averages of daily minimum temperatures (4–10°C) were associated with a cumulative risk increase over 8-weeks (6°C RR 1.04: 95% CI 1.01-1.06) (Figure 3A). Relative humidity provided a better model fit (difference in Watanabe–Akaike Information Criterion; ΔWAIC: 77.7) than absolute humidity (the absolute amount of vapor in air g/m^3) which in contrast to relative humidity does not account for the temperature-dependent variation in the air's capacity to retain water. Absolute humidity had a stronger association at low values (4 g/m^3 cumulative RR 1.23: 95% CI 1.16-1.30) with an acute increase in disease risk until a 2-week lag time. For relative humidity the cumulative risk was non-linear. The highest risk occurred at moderate humidity levels (34%- 47%) with a maximum cumulative RR of 1.05 (95% CI 1.03–1.07) at 39.8% relative to the median (Figure 3B). There was a strong protective effect of high relative humidity with RR decreasing to 0.77 (95% CI 0.73-0.88) at 74.8% relative humidity ***(Figure 3B)****. These differences in apparent effect magnitude between absolute and relative humidity should be interpreted cautiously as they depend on the distribution of each variable. The weaker relative effects observed for relative humidity likely reflect its dependence on temperature, suggesting that temperature partially mediates humidity-related effects* ***(Figure 3B)(Figure S5)(Figure S21)(Table S5)****.*
Increasing precipitation and a higher Standardised Precipitation and Evapotranspiration Index (SPEI) (suggesting reduced drought conditions) decreased IPD risk (negative associations) ***(Figure S6A-B)****. Increasing average wind speed increased IPD risk (positive association), while increasing ozone concentrations (ozone increases with increasing temperatures) decreased disease risk; concordant with decreasing risk in the summer months* ***(Figure S6C-D)(Figure S7)****. "*

We also adjusted **Figure 3 (above in response 1.1)** and **Figure S6** for clarity:

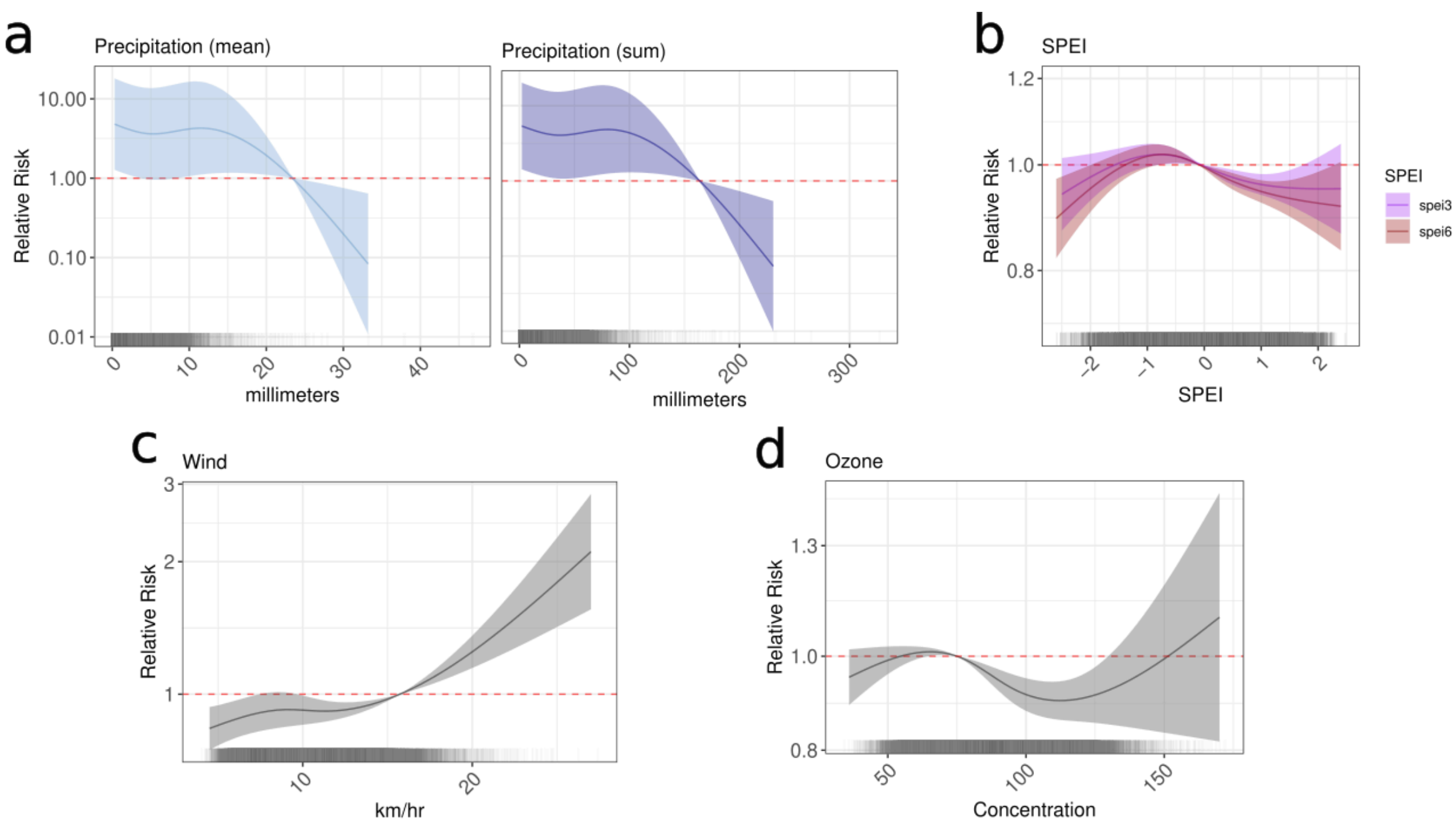

Figure S6. Cumulative 8-week relative risk with models fit to weekly district level IPD case data with the sociodemographic base model and these meteorological DLNMs for (a) precipitation mean (left; light blue) precipitation sum (right; dark blue), and (b) standard precipitation evapotranspiration index (SPEI) 3 months (purple) and 6 months (brown) (c) wind kilometers per hour (km/hr) (d) ozone (μg/m^3).

Further, we agree that extreme rainfall events, flooding, or tropical cyclones may have complex effects on infectious disease dynamics through mechanisms such as population displacement, water contamination, and disruption of healthcare services. However, the present study did not specifically examine extreme weather events, which occur relatively infrequently and may require dedicated event-based or disaster-focused analyses.

To address this point, we have added text to the manuscript acknowledging that extreme rainfall events and flooding could influence infectious disease dynamics in ways not captured by the meteorological indicators used in this analysis, and that future research could explore these relationships in more detail in the South African context:

Lines 579-582 (tracked changes version):

"It is also important to note that extreme rainfall events and flooding may also influence IPD dynamics through mechanisms such as population displacement, water contamination, and disruption of health services[60]. These complex effects were not specifically examined in the present analysis and warrant further investigation."

60. Bellos, A. *et al.* The burden of acute respiratory infections in crisis-affected populations: a systematic review. *Confl. Health* **4**, 3 (2010).

Query 1.8 The rationale for using daily maximum/minimum temperature (tasmax/ tasmin) as the temperature metrics, rather than daily mean temperature, was not provided. Different temperature metrics

may capture distinct physiological stresses (e.g., heat stress vs. cold exposure). The choice should be biologically justified or comparative models should be tested.

Response 1.8 Thank you for this comment. We have now explicitly evaluated models using daily maximum (tasmax), minimum (tasmin), and mean temperature to clarify their distinct relationships with invasive pneumococcal disease (IPD) risk. This is now also included in **Figure 3A** and **Figure S5C**. We have also included a paragraph describing these differences in the main text. We evaluated multiple metrics but ultimately included mean temperature in the air pollution models to account for its confounding effects with air pollution.

Lines 220-224 (tracked changes version):
"Weeks with a mean daily maximum temperature of 31°C were associated with a 5% increased risk of IPD relative to the median, rising to an 11% increased risk at 38°C. Similarly, mean weekly temperatures of 24°C were associated with a 5% risk increase (RR 1.05: 95% CI 1.03-1.08)(Figure S5C). In contrast, weekly averages of daily minimum temperatures (4–10°C) were associated with a cumulative risk increase over 8-weeks (6°C RR 1.04: 95% CI 1.01-1.06) (Figure 3A)."

Query 1.9 Line 413, the phrase "SARS-CoV-2 pandemic" was used. For consistency and clarity within the public health and epidemiological context of this study, it is recommended to use the more common term "COVID-19 pandemic."

Response 1.9 Thank you for this comment. This has now been changed to "COVID-19 pandemic".

Query 1.10 Line 443, "The population sizes per year per district were sourced from the 2023 Statistics South Africa projections". This description raises a concern about temporal appropriateness. Since the study period spans 2005-2023, applying a single set of projections from the endpoint year (2023) to represent population counts for all preceding years would be incorrect and may introduce significant inaccuracy.

Response 1.10 We included yearly population sizes for each year from 2005-2023 and each district. We sourced this data from the 2023 Statistics South Africa census. This is clarified in the Sociodemographic Data section in the Methods: "*The population size per year per district (from 2005-2023), were sourced from the 2023 Statistics South Africa projections"*

Reviewer #2 (Remarks to the Author):

This study stands out for its integration of large-scale epidemiological data (over 59,000 IPD cases spanning 15+ years) with genomic sequencing (4,350 isolates) and environmental variables in a high-burden setting like South Africa. An impressive study given the large number of research techniques used. While prior research has linked air pollution (PM2.5) and meteorological factors (humidity) to respiratory infections, including pneumococcal disease, the novelty here lies in explicitly accounting for

microbial diversity, specifically, serotypes and genomic lineages, as mediators of environmental impacts on IPD risk. This is important for climate sensitive disease researchers.

Dear Reviewer #2,

Thank you for your insights and comments particularly around inclusion of hypotheses for the mechanisms driving different effects across serotypes and lineages, and addressing sources of potential confounding. We believe that your insights have substantially strengthened the work.

Query 2.1 The key issue of the study is that human activity confounds everything, pollution from industry/traffic correlates with urban density (which affects transmission), and metrology influences behavior (indoor crowding in dry seasons). The models control for density and seasonality but can't fully disentangle reverse causation or unmeasured confounders (co-infections like influenza, socioeconomic factors). The lagged effects suggest plausible mechanisms (PM2.5 impairing immunity or promoting bacterial virulence), but these are inferred, not experimentally proven. Associations could indeed be proxies for behavioral/socioeconomic factors, and the authors emphasize this by framing results as "relationships" rather than direct causes.

Response 2.1

Thank you for this comment. We agree that factors associated with urban environments, such as population density, indoor crowding, and other unmeasured socioeconomic or household characteristics, may act as potential confounders in the relationship between air pollution and IPD. We acknowledge this limitation in the Discussion (lines 573-579).

Lines 573-579 (tracked changes version):

"A limitation of this study is the lack of granular data on indoor air pollution exposure. However, given that indoor and ambient pollutants are often closely correlated, our results likely capture the core exposure-outcome association, though excludes the precise contribution of indoor air pollution[57,58]. We also lacked primary data on indoor household factors, but include population density and spatiotemporal random effects as proxies for some of this unobserved risk. Our inability to account for sub-district level variations in household factors is a further limitation, especially given the known differences in household air pollution exposure when stratified by sex[59]."

57. González-Martín, J., Kraakman, N. J. R., Pérez, C., Lebrero, R. & Muñoz, R. A state–of–the-art review on indoor air pollution and strategies for indoor air pollution control. *Chemosphere* **262**, 128376 (2021).
58. Benyon, M. *et al.* Household PM2.5 in a South African urban and rural setting: A comparative analysis using low-cost sensors. *Atmospheric Pollut. Res.* **16**, 102459 (2025).
59. Somboonsin, P. P. & Baffour, B. Spatial Inequities in Women's Health: The Global Impact of Household Air Pollution from Solid Fuels. *Water. Air. Soil Pollut.* **236**, 934 (2025).

To partially address this concern, we examined the correlation between population density and IPD incidence and found only a weak Pearson correlation ($r = 0.20$), suggesting that population density alone is not a primary correlate of IPD in our dataset (Figure S4A). Importantly, many sociodemographic and household characteristics are relatively stable over time, and from week to week, whereas air pollution levels vary substantially within a week.

Although we did not have primary data on indoor household characteristics, the inclusion of population density and spatiotemporal random effects in our models serves as a proxy for some unobserved sources of variation in risk. Nevertheless, we acknowledge that our study cannot account for sub-district level variation in household crowding or other socioeconomic factors, which may influence IPD risk and therefore represents an important limitation of our analysis.

Query 2.2 While not revolutionary in proving pollution "causes" IPD (ecological fallacy looms), its novelty in microbial-aware modeling makes it a step forward. Does S. Africa follow global trends, or is this just having internal validity only?

Response 2.2 We agree that the "microbial-aware" modeling represents a step forward. This is particularly important given the mixed evidence on how meteorological factors influence pneumococcal dynamics. Our study helps fill a gap in the literature regarding the effects of both meteorological and air pollution factors on these dynamics (Belman et al., 2026, under review; SSRN). There are some similarities to global trends. For example, short-lag positive associations with temperature have been observed, consistent with findings from Jusot et al., 2017 (Nigeria) and Qin et al., 2021 (China). However, the broader consensus still points to a negative association between temperature and disease incidence, where lower temperatures tend to increase disease risk. It is important to note that multiple environmental factors often interact, and their effects can vary by location. Understanding the timing and structure of these relationships, in each setting, contributes to solving the mechanistic puzzle of how environmental factors shape disease dynamics. We have added some of this background from our recent scoping review to the Introduction (lines 59-72) and in the Discussion (lines 465-470 & 488-500).

Lines 59-72 (tracked changes version):

Introduction:

"Existing evidence around the impact of environmental factors on pneumococcal disease is heterogenous[13]. Most studies focus on temperature, generally showing that cold temperatures increase disease in temperate climates, although positive associations (higher disease with higher temperatures) have been reported in arid settings[14–16]. Evidence on rainfall and humidity is similarly mixed: while many studies suggest that increasing rainfall and humidity decrease pneumococcal disease incidence, the opposite effect is found in settings such as Senegal[17], Texas[18], Spain[16], and New Zealand[19] where high levels of rainfall and humidity are associated with increasing disease. Air pollution is widely considered to increase IPD risk, however the mechanisms are poorly understood[20]. A recent scoping review identified only thirteen epidemiological studies (as of January 2026) examining particulate matter (e.g. $PM_{2.5}$ and PM_{10}) with just six finding a positive relationship[13,14,21–25]. Most relied on simple correlations, few assessed delayed effects (lag times from exposure to outcome), or controlled for confounding meteorological effects[18,21,22]. Overall, important gaps remain, including limited understanding of non-linear and lagged effects, interactions between environmental factors, and variation across age groups, disease presentations, or serotypes."

13. Belman, S. *et al.* The Influence of Climate and Air Pollution on Pneumococcal Dynamics: A Scoping Review. SSRN Scholarly Paper at https://doi.org/10.2139/ssrn.6400926 (2026).
14. Jusot, J.-F. *et al.* Airborne dust and high temperatures are risk factors for invasive bacterial disease. *J. Allergy Clin. Immunol.* **139**, 977-986.e2 (2017).
15. Qin, T. *et al.* Incidence, Etiology, and Environmental Risk Factors of Community-Acquired Pneumonia Requiring Hospitalization in China: A 3-Year, Prospective, Age-Stratified, Multicenter Case-Control Study. *Open Forum Infect. Dis.* **8**, ofab499 (2021).
16. García-Rey, C., Fenoll, A., Aguilar, L. & Casal, J. Effect of social and climatological factors on antimicrobial use and Streptococcus pneumoniae resistance in different provinces in Spain. *J. Antimicrob. Chemother.* **54**, 465–471 (2004).
17. Diouf, F. S. *et al.* Seasonal variation of asymptomatic viral and bacterial nasopharyngeal carriage in rural Senegal. *J. Infect. Public Health* **17**, 922–928 (2024).
18. Kim, P. E. *et al.* Association of Invasive Pneumococcal Disease with Season, Atmospheric Conditions, Air Pollution, and the Isolation of Respiratory Viruses. *Clin. Infect. Dis.* **22**, 100–106 (1996).

19. Murdoch D.R. & Jennings L.C. Association of respiratory virus activity and environmental factors with the incidence of invasive pneumococcal disease. *J. Infect.* **58**, 37 EP – 46 (2009).
20. Beentjes, D., Shears, R. K., French, N., Neill, D. R. & Kadioglu, A. Mechanistic Insights into the Impact of Air Pollution on Pneumococcal Pathogenesis and Transmission. *Am. J. Respir. Crit. Care Med.* **206**, 1070–1080 (2022).
21. Dherani, M. K. *et al.* Association between household air pollution and nasopharyngeal pneumococcal carriage in Malawian infants (MSCAPE): a nested, prospective, observational study. *Lancet Glob. Health* **10**, e246–e256 (2022).
22. Li, C. *et al.* Investigating the Distribution and Antibiotic Resistance of Bacterial Pathogens in Clinical Specimens from a Chinese Maternal and Child Hospital: The Role of Environmental Factors. *Infect. Drug Resist.* **17**, 2261–2272 (2024).
23. Murdoch D.R. & Jennings L.C. Association of respiratory virus activity and environmental factors with the incidence of invasive pneumococcal disease. *J. Infect.* **58**, 37 EP – 46 (2009).
24. Yang, T. *et al.* Epidemiological characteristics and meteorological factors of acute respiratory infections (ARIs) in hospitalized children in eastern Guangdong, China. *Sci. Rep.* **14**, 25518 (2024).
25. Zhou, Z., Fang, C., Li, J., Zhou, M. & Chen, X. Ambient NO(2) is associated with Streptococcus pneumoniae-induced pneumonia in children and increases the minimum inhibitory concentration of penicillin. *Int. J. Biometeorol.* **66**, 87–96 (2022).
26. Choe, Y. J., Smit, M. A. & Mermel, L. A. Seasonality of respiratory viruses and bacterial pathogens. *Antimicrob. Resist. Infect. Control* **8**, 125 (2019).

Discussion:

Lines 465-470 (tracked changes version):

"Our study corroborates some previous findings regarding the effects of meteorological factors and IPD, such as increased risk at lower values for humidity, temperature and precipitation, contributing to a growing pool of heterogeneous evidence across different sociodemographic and climatic regions[13]. It is likely that these effects depend on additional factors such as the climatic region, age group, and other underlying coinfections or immunological factors."

13. Belman, S. *et al.* The Influence of Climate and Air Pollution on Pneumococcal Dynamics: A Scoping Review. SSRN Scholarly Paper at https://doi.org/10.2139/ssrn.6400926 (2026).

Lines 488-500 (tracked changes version):

"For serotypes that are already colonising, exposure to high levels of air pollution may facilitate invasion. Therefore, serotypes with a high burden in populations exposed to elevated air pollution may exhibit more immediate effects, whereas serotypes that are not yet widely colonising may show delayed responses. Evidence on the impact of air pollution on carriage acquisition remains mixed. Several previous studies have identified increased epithelial adhesion, biofilm formation, and bacterial burden with air pollution[46–49], while others find a decrease in carriage acquisition with increasing $PM_{2.5}$ concentrations[14,47]. The acute effects which we found for serotype 4, 23F, and 19A indicate that there may be a different mechanism for their invasion than other serotypes. Serotype 4 has been particularly notable in causing invasive disease among adults despite its inclusion in the vaccine across the globe[7,48,49]. This may reflect its exacerbation by environmental factors. This serotype has high proportions of the gene psrP, an adhesin and a known virulence factor which together with microabrasions from exposure to $PM_{2.5}$[50–52] may increase invasion with no lag time[53], although this alone is not sufficient given that psrP is present across a range of lineages."

7. Lekhuleni, C. *et al.* Impact of pneumococcal conjugate vaccines on invasive pneumococcal disease-causing lineages among South African children. *Nat. Commun.* **15**, 8401 (2024).
14. Jusot, J.-F. *et al.* Airborne dust and high temperatures are risk factors for invasive bacterial disease. *J. Allergy Clin. Immunol.* **139**, 977-986.e2 (2017).
46. de Oliveira, J. D. *et al.* Influence of environmental dust in transformation capacity of Streptococcus pneumoniae. *Genet. Mol. Biol.* **44**, e20200028 (2020).
47. Yadav, M. K., Go, Y. Y., Chae, S.-W., Park, M. K. & Song, J.-J. Asian Sand Dust Particles Increased Pneumococcal Biofilm Formation in vitro and Colonization in Human Middle Ear Epithelial Cells and Rat Middle Ear Mucosa. *Front. Genet.* **11**, (2020).
48. Kellner, J. D. *et al.* Whole-Genome Analysis of Streptococcus pneumoniae Serotype 4 Causing Outbreak of Invasive Pneumococcal Disease, Alberta, Canada. *Emerg. Infect. Dis.* **27**, 1867–1875 (2021).
49. Cuypers, L. *et al.* Rapid increase in serotype 4 invasive pneumococcal disease among vulnerable young male adults in Belgium (2020-2024). *Int. J. Infect. Dis. IJID Off. Publ. Int. Soc. Infect. Dis.* **163**, 108216 (2026).
50. Shivshankar, P., Sanchez, C., Rose, L. F. & Orihuela, C. J. The Streptococcus pneumoniae adhesin PsrP binds to Keratin 10 on lung cells. *Mol. Microbiol.* **73**, 663–679 (2009).

51. Yang, T. *et al.* Epidemiological characteristics and meteorological factors of acute respiratory infections (ARIs) in hospitalized children in eastern Guangdong, China. *Sci. Rep.* **14**, 25518 (2024).
52. Zhou, Z., Fang, C., Li, J., Zhou, M. & Chen, X. Ambient NO(2) is associated with Streptococcus pneumoniae-induced pneumonia in children and increases the minimum inhibitory concentration of penicillin. *Int. J. Biometeorol.* **66**, 87–96 (2022).
53. Dherani, M. K. *et al.* Association between household air pollution and nasopharyngeal pneumococcal carriage in Malawian infants (MSCAPE): a nested, prospective, observational study. *Lancet Glob. Health* **10**, e246–e256 (2022).

Reviewer #3 (Remarks to the Author):

A large study of pneumococcal infections across a large number of hospitals over a prolonged period; it only refers to invasive infections (sepsis and meningitis mainly) and how the incidence of infection is affected by pollution and climate and how these interact with strain background. The data come from large datasets across South Africa over a significant period of time and the relationship between infection, pollution, and climatic conditions looks pretty solid. The specific associations with age serotype and GPSC type are likely to be less strong due to the reduction in numbers as the comparisons are broken down within the whole research cohort; these however are the more intriguing (and perhaps more novel - I am not clear what is known about PM2.5 and pneumococcus although there is a clear relation to pneumonia in some studies - this is one area the manuscript should be described better as important background). Yet the biological basis for these associations which often differ between strains etc is not clear to me, and the authors have not really said whether they expected these patterns or whether they are unexpected.

Dear Reviewer #3,
Thank you for your thoughtful and detailed assessment of our work. We appreciate your recognition of the strengths of the dataset and the observed relationships between invasive pneumococcal disease, environmental exposures, and climate. In particular your suggestions to better contextualize existing evidence on particulate matter and pneumococcal infections, and to clarify the biological basis and expectations for strain-specific differences, have been very helpful. In response, we have expanded the background and clarified our interpretation of these findings.

Major issues
Query 3.1.1 The data does not include pneumonia presumably as the microbiological diagnostic accuracy of pneumococcal pneumonia is poor, and this is not discussed at all. Yet pneumonia is the likely dominant form of pneumococcal infection and is also more obviously likely to be affected by climate. SOme text in the discussion on this I think would be helpful, even though extrapolating from sepsis data to pneumonia must be largely intelligent guesswork. And it may be that the increase in septicaemia largely reflects an increase in pneumonia as that is the cause of most septicaemia in adults? Although increases in other IPD is likely to be largely independent of pneumonia as they usually are not concurrent diagnoses; some sort of mechanism by which colonisation is more able to translocate into the blood or there are higher rates of colonisation??

Response 3.1.1 Thank you for this comment. As you stated we only include bacteremic pneumonia in this study, which is a limitation. It will be interesting to understand what the differences in effects are with regards to non-bacteremic pneumococcal pneumonia as compared to bacteremic pneumococcal pneumonia in future work. We have clarified that only bacteremia pneumonia is included here and the limitation of not having non-invasive pneumonia in this study.

Line 635 (tracked changes version)

"Bacteremic cases do include bacteremic pneumonia but non-bacteremic pneumonia is not included."

Lines 595-603 (tracked changes version)

"We did not include carriage isolates in this analysis, as the focus of GERMS-SA is on invasive disease. Consequently, our results pertain only to invasive pneumococcal disease (IPD) lineages and serotypes. This also means that cases of non-bacteraemic pneumonia are not captured in this study. This exclusion may be skewing the impact of some of our environmental associations as a whole. Understanding the relationships between environmental factors and bacteremic versus non-bacteremic pneumonia is an important direction for future work."

Query 3.1.2 What is known about pollution and climate and pneumococcal nasopharyngeal colonisation rates and density? The latter is important to discuss somewhere

Response 3.1.2 We agree it is important to acknowledge previous work on environmental factors and colonisation rates and density as colonisation is a pivotal component of pneumococcal dynamics and a necessary prerequisite for disease. There is some existing evidence on the impact of air pollution on colonization which we have incorporated into the Discussion.

Lines 488-494 (tracked changes version)

"For serotypes that are already colonising, exposure to high levels of air pollution may facilitate invasion. Therefore, serotypes with a high burden in populations exposed to elevated air pollution may exhibit more immediate effects, whereas serotypes that are not yet widely colonising may show delayed responses. Evidence on the impact of air pollution on carriage acquisition remains mixed. Several previous studies have identified increased epithelial adhesion, biofilm formation, and bacterial burden with air pollution[46–49], while others find a decrease in carriage acquisition with increasing $PM_{2.5}$ concentrations[14,47]."

14. Jusot, J.-F. *et al.* Airborne dust and high temperatures are risk factors for invasive bacterial disease. *J. Allergy Clin. Immunol.* **139**, 977-986.e2 (2017).
46. de Oliveira, J. D. *et al.* Influence of environmental dust in transformation capacity of Streptococcus pneumoniae. *Genet. Mol. Biol.* **44**, e20200028 (2020).
47. Yadav, M. K., Go, Y. Y., Chae, S.-W., Park, M. K. & Song, J.-J. Asian Sand Dust Particles Increased Pneumococcal Biofilm Formation in vitro and Colonization in Human Middle Ear Epithelial Cells and Rat Middle Ear Mucosa. *Front. Genet.* **11**, (2020).
48. Kellner, J. D. *et al.* Whole-Genome Analysis of Streptococcus pneumoniae Serotype 4 Causing Outbreak of Invasive Pneumococcal Disease, Alberta, Canada. *Emerg. Infect. Dis.* **27**, 1867–1875 (2021).
49. Cuypers, L. *et al.* Rapid increase in serotype 4 invasive pneumococcal disease among vulnerable young male adults in Belgium (2020-2024). *Int. J. Infect. Dis. IJID Off. Publ. Int. Soc. Infect. Dis.* **163**, 108216 (2026).

Query 3.2. The paper really needs a summary of what is already know about pollution, humidity, season, and air temperature and pneumococcal infections and other infectious diseases to make it clear what is new in this manuscript

Response 3.2 We have now added a section in the introduction addressing what is previously known and the gap that this study fills (lines 59-72). Prior to this study, there was a major gap regarding the impact of environmental factors across different serotypes, disease types, or age groups for pneumococcal disease. Most evidence around the impact of air pollution on pneumococcal disease dynamics includes positive associations, however there are some notable studies which find negative relationships including a study in Spain finding a negative association with pneumococcal infection (Sahuquillo-Arce et al., 2017) and another in China (Qin et al., 2021). We include this summary and also now cite a recent Scoping Review (Belman et al., 2026), which is currently under peer review (http://dx.doi.org/10.2139/ssrn.6400926).

Introduction:

Lines 59-72 (tracked changes version):

"Existing evidence around the impact of environmental factors on pneumococcal disease is heterogenous[13]. Most studies focus on temperature, generally showing that cold temperatures increase disease in temperate climates, although positive associations (higher disease with higher temperatures) have been reported in arid settings[14–16]. Evidence on rainfall and humidity is similarly mixed: while many studies suggest that increasing rainfall and humidity decrease pneumococcal disease incidence, the opposite effect is found in settings such as Senegal[17], Texas[18], Spain[16], and New Zealand[19] where high levels of rainfall and humidity are associated with increasing disease. Air pollution is widely considered to increase IPD risk, however the mechanisms are poorly understood[20]. A recent scoping review identified only thirteen epidemiological studies (as of January 2026) examining particulate matter (e.g. $PM_{2.5}$ and PM_{10}) with just six finding a positive relationship[13,14,19,21–24]. Most relied on simple correlations, few assessed delayed effects (lag times from exposure to outcome), or controlled for confounding meteorological effects[18,21,22]. Overall, important gaps remain, including limited understanding of non-linear and lagged effects, interactions between environmental factors, and variation across age groups, disease presentations, or serotypes."

13. Belman, S. *et al.* The Influence of Climate and Air Pollution on Pneumococcal Dynamics: A Scoping Review. SSRN Scholarly Paper at https://doi.org/10.2139/ssrn.6400926 (2026).
14. Jusot, J.-F. *et al.* Airborne dust and high temperatures are risk factors for invasive bacterial disease. *J. Allergy Clin. Immunol.* **139**, 977-986.e2 (2017).
15. Qin, T. *et al.* Incidence, Etiology, and Environmental Risk Factors of Community-Acquired Pneumonia Requiring Hospitalization in China: A 3-Year, Prospective, Age-Stratified, Multicenter Case-Control Study. *Open Forum Infect. Dis.* **8**, ofab499 (2021).
16. García-Rey, C., Fenoll, A., Aguilar, L. & Casal, J. Effect of social and climatological factors on antimicrobial use and Streptococcus pneumoniae resistance in different provinces in Spain. *J. Antimicrob. Chemother.* **54**, 465–471 (2004).
17. Diouf, F. S. *et al.* Seasonal variation of asymptomatic viral and bacterial nasopharyngeal carriage in rural Senegal. *J. Infect. Public Health* **17**, 922–928 (2024).
18. Kim, P. E. *et al.* Association of Invasive Pneumococcal Disease with Season, Atmospheric Conditions, Air Pollution, and the Isolation of Respiratory Viruses. *Clin. Infect. Dis.* **22**, 100–106 (1996).
19. Murdoch D.R. & Jennings L.C. Association of respiratory virus activity and environmental factors with the incidence of invasive pneumococcal disease. *J. Infect.* **58**, 37 EP – 46 (2009).
20. Beentjes, D., Shears, R. K., French, N., Neill, D. R. & Kadioglu, A. Mechanistic Insights into the Impact of Air Pollution on Pneumococcal Pathogenesis and Transmission. *Am. J. Respir. Crit. Care Med.* **206**, 1070–1080 (2022).
21. Dherani, M. K. *et al.* Association between household air pollution and nasopharyngeal pneumococcal carriage in Malawian infants (MSCAPE): a nested, prospective, observational study. *Lancet Glob. Health* **10**, e246–e256 (2022).
22. Li, C. *et al.* Investigating the Distribution and Antibiotic Resistance of Bacterial Pathogens in Clinical Specimens from a Chinese Maternal and Child Hospital: The Role of Environmental Factors. *Infect. Drug Resist.* **17**, 2261–2272 (2024).
23. Yang, T. *et al.* Epidemiological characteristics and meteorological factors of acute respiratory infections (ARIs) in hospitalized children in eastern Guangdong, China. *Sci. Rep.* **14**, 25518 (2024).
24. Zhou, Z., Fang, C., Li, J., Zhou, M. & Chen, X. Ambient NO(2) is associated with Streptococcus pneumoniae-induced pneumonia in children and increases the minimum inhibitory concentration of penicillin. *Int. J. Biometeorol.* **66**, 87–96 (2022).

Discussion:

Lines 465-470 (tracked changes version):

"Our study corroborates some previous findings regarding the effects of meteorological factors and IPD, such as increased risk at lower values for humidity, temperature and precipitation, contributing to a growing pool of heterogeneous evidence across different sociodemographic and climatic regions[13]. It is likely that these effects depend on additional factors such as the climatic region, age group, and other underlying coinfections or immunological factors."

13. Belman, S. *et al.* The Influence of Climate and Air Pollution on Pneumococcal Dynamics: A Scoping Review. SSRN Scholarly Paper at https://doi.org/10.2139/ssrn.6400926 (2026).

Query 3.3 Often the text lacks clarity and uses jargon that really makes it harder to follow the meaning that it could be if rewritten slightly in places. There are multiple examples of this hence I am not stating each one in this review.

Response 3.3 Thank you for this comment, we have thoroughly revised the manuscript and made changes throughout to reduce jargon and improve clarity.

Query 3.4 Some discussion of the potential mechanisms underpinning differences between serotypes/GPSC, age groups, and disease in the effects of environmental factors on disease incidence would be useful, and important to indicate there is biological plausibility for some of these differences which otherwise are pretty confusing (why are older subjects more susceptible? how can pollution levels have different effects on individual pneumococcal serotypes / strains or disease type? With serotype in particualr it is odd that NVT and PCV7 serotypes cluster together and have the opposite effect on timing of their effect compared to PCV13)

Response 3.4 Thank you for this comment. The differences in effect between serotypes and GPSCs is biologically plausible given the massive variability in virulence, carriage duration and burden, genomic content, age group affected, and disease type across the diversity of the pneumococcus. We have added a section to the Discussion regarding possible drivers of differences including genomic variability, cumulative exposure across sex, and particular serotypes with distinct life histories. We have now added several sections to the discussion highlighting these hypotheses.

Regarding the clustering of PCV7 and NVT types compared to PCV13 types, it is possible that this merely reflects the life histories of the serotypes they comprise rather than being a direct result of vaccination. For example, there is previous evidence that PCV13 serotypes had a larger decreased population fitness after vaccination than PCV7 types (Belman et al., 2023) and that the reductions for PCV13 serotypes were greater than for PCV7 (Dagan et al., 2013). Therefore, the lagged response to $PM_{2.5}$ may reflect the increased protection against carriage for PCV13 types compared to for PCV7 or NVTs, such that there is a more immediate effect for the types already colonizing (PCV7 or NVTs). We have added a section in the main results (lines 363-371).

Lines 363-371 (tracked changes version):

"The similarly acute risk for PCV7 and NVT types, compared to the lagged risk for PCV13 types, may reflect different colonization burdens in the population. For example, there is previous evidence that PCV13 serotypes had a larger decreased fitness after vaccination than PCV7 types and that there were greater reductions in PCV13 serotype carriage than for PCV7 serotypes[36]. A possible mechanism for the lagged response to $PM_{2.5}$ among PCV13 types may be that they have lower colonization rates in the population leading to a delayed IPD risk from $PM_{2.5}$[37] (Figure 4D). These effects persisted when we stratified by pre- and post-PCV periods implying a difference in

mechanism not just changing serotype proportions (Figure S16A). When further stratified by specific serotypes variable underlying dynamics emerged."

36. Dagan, R. et al. Comparative Immunogenicity and Efficacy of 13-Valent and 7-Valent Pneumococcal Conjugate Vaccines in Reducing Nasopharyngeal Colonization: A Randomized Double-Blind Trial. Clin. Infect. Dis. 57, 952–962 (2013).
37. Javaid, N. et al. Population genomics of pneumococcal carriage in South Africa following the introduction of the 13-valent pneumococcal conjugate vaccine (PCV13) immunization. Microb. Genomics 8, 000831 (2022).

Query 3.5 Gauteng and Cape Town had RR of 10+ which I presume is for rates of IPD per 1000 population; this must be due to different ascertainment rates for IPD (plausible if there are large differences in the level of medical provision) as such a large variation in RR seems unlikely just due to geography / different demographics unless the vast majority of HIV +ve people live at these two locations? How much of the data is driven by the massively higher burden of infection in Gauteng and to a lesser extent Cape Town (what % of all cases comes from this province +/- Cape Town? would be good to state in the results), and is this increase in IPD in that province a real thing or artificial due to reduced ascertainment in other provinces? This may need reassessing the results with or without Gauteng's contribution?

Response 3.5 Thank you for this comment. We have now included the percentage of disease from each province in the results. It includes 41% of total IPD from Gauteng, 18% from Western Cape, as well as 11 and 9% from KwaZulu-Natal and Eastern Cape. These provinces include the major South African cities of Johannesburg, Cape Town, Durban, and Port Elizabeth respectively. We have also included additional sensitivity analyses comparing models for Gauteng alone, South Africa as a whole (Figure S9-S11) and for South Africa excluding Gauteng province (Figure S11). Interestingly, we find similar cumulative risks from 50μg/m^3 of $PM_{2.5}$ for all three models. However, there is evidence for 3-week lagged effects from $PM_{2.5}$ for Gauteng alone whereas for all of South Africa and models excluding Gauteng the risk is more immediate (Figure S11B). This may reflect different patterns in $PM_{2.5}$ effects in regions with chronic rather than acute exposure.

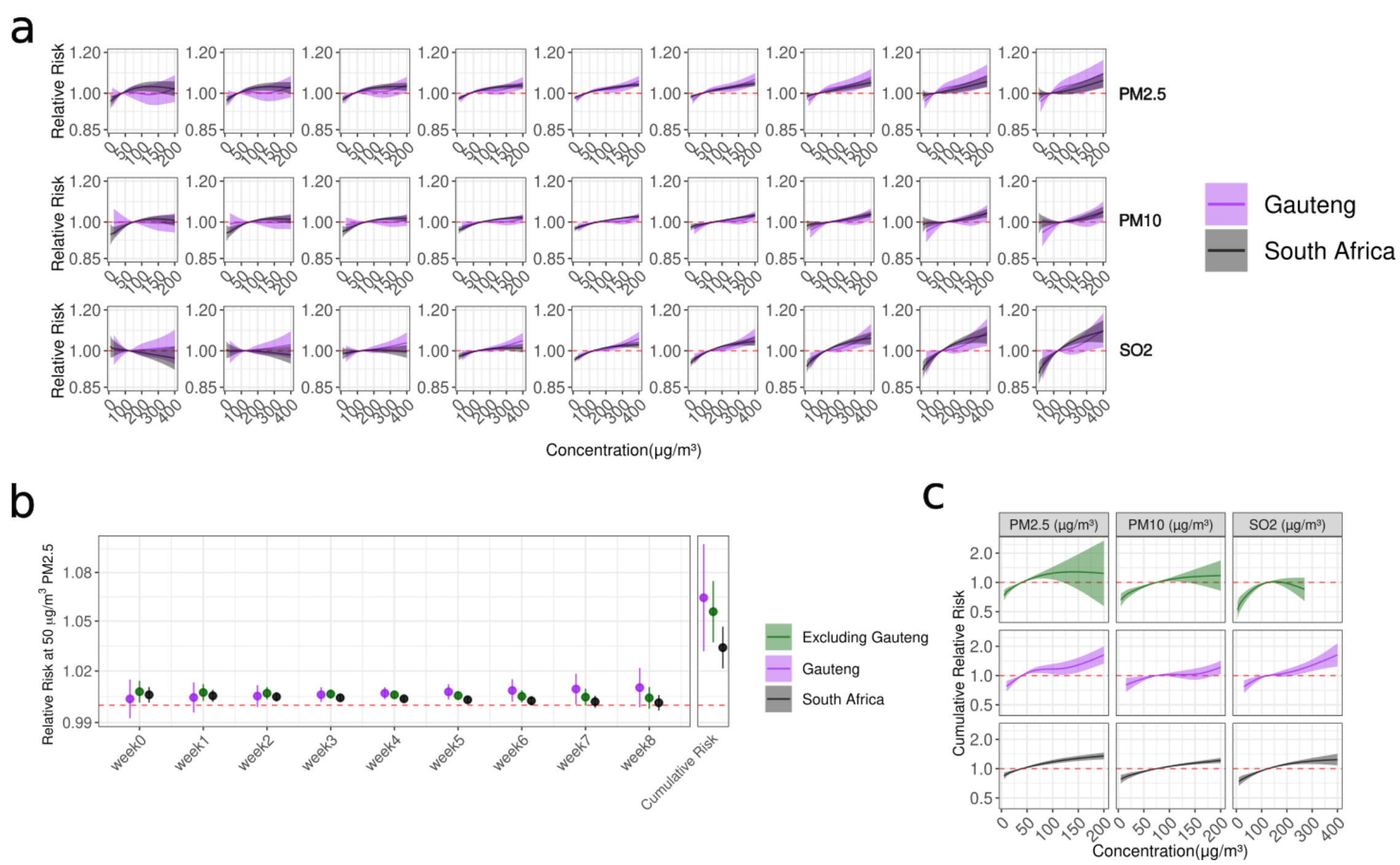

Figure S11. Comparison of models run on all of South Africa (black), Gauteng alone (purple) and for b-c excluding Gauteng (green). *These models are fit to weekly district level case data with the sociodemographic base model, temperature and humidity confounders and the DLNM crossbasis for each pollutant**.** (a) exposure-response curves across 8 weeks of lags for relative risk from air pollutant exposure. for Gauteng alone (purple) and South Africa overall (black) for $PM_{2.5}$ (top), PM_{10} (middle), and SO_2 (bottom). (b) Relative risk at 50μg/m^3 of $PM_{2.5}$ for each of the lag weeks from 0-8 and the cumulative risk. (c) The cumulative relative risk for each of $PM_{2.5}$ (left), PM_{10} (middle), and SO_2 (right) excluding Gauteng (green), Gauteng alone (purple), and for South Africa overall (black).*

Query 3.6 The effects of humidity (25%) were a lot stronger than those for PM2.5(4%) - this probably should be brought up in the discussion as it is important for the reader to be aware of large differences in strength of effect between different environmental conditions.

Response 3.6 We note that the magnitude of estimated effects depends on the units of each covariate, and therefore direct comparison between relative humidity (%) and PM2.5 (µg/m³) can be misleading. To address this, we have reported effects for meaningful increments (such as 50 µg/m³ of weekly $PM_{2.5}$) which allows a more appropriate comparison of effect sizes across environmental variables. We have clarified the paragraph discussing these effects to make the risks and the comparability clearer (lines 223-252 & lines 468-470).

Lines 223-252 (tracked changes version):

*"Relative humidity provided a better model fit (difference in Watanabe–Akaike Information Criterion; ΔWAIC: 77.7) than absolute humidity (the absolute amount of vapor in air g/m^3) which in contrast to relative humidity does not account for the temperature-dependent variation in the air's capacity to retain water. Absolute humidity had a stronger association at low values (4 g/m^3 cumulative RR 1.23: 95% CI 1.16-1.30) with an acute increase in disease risk until a 2-week lag time. For relative humidity the cumulative risk was non-linear. The highest risk occurred at moderate humidity levels (34%- 47%) with a maximum cumulative RR of 1.05 (95% CI 1.03–1.07) at 39.8% relative to the median (Figure 3B). There was a strong protective effect of high relative humidity with RR decreasing to 0.77 (95% CI 0.73-0.88) at 74.8% relative humidity **(Figure 3B)**. These differences in apparent effect magnitude between absolute and relative humidity should be interpreted cautiously as they depend on the distribution of each variable.*

The weaker relative effects observed for relative humidity likely reflect its dependence on temperature, suggesting that temperature partially mediates humidity-related effects ***(Figure 3B)(Figure S5)(Figure S21)(Table S5)****."*

Lines 468-470 (tracked changes version):
"We find that the protective effect of high humidity is the strongest cumulative 8-week meteorological effect size we identified, providing around a 25% risk reduction for IPD. Effect sizes for temperature variables and air pollution are more modest (below 10%)."

Minor issues (this is not an exhaustive list of areas of text that would benefit from some revision for clarity):

Q3.7 abstract lines 31-32 - it is not clear what this sentence means (the technical jargon is hard to follow)
R3.7 We agree that this is unclear. We have edited the abstract to increase clarity.
Lines 32-33 (tracked changes version):
"Weekly $PM_{2.5}$ exposure at 50 μg/m³ was associated with a 4% increase in cumulative risk. The timing of effects differed across bacterial serotypes and lineages."

Q3.8 line 35 Streptococcus pneumoniae not italicised
R3.8 We have now italicized *S. pneumoniae* throughout.

Q3.9 line 38 I think of colonisation as being the same as asymptomatic carriage so delete one or other of these
R3.9 We have clarified the intended difference here.
Lines 42-43 (tracked changes version):
"The progression from acquisition, to asymptomatic carriage, to invasive disease is mediated by a combination of environmental drivers, microbial virulence, and host immunity."

Q3.10 lines 46 to 47 not sure that '...in part due to the extensive diversity...' bit of the sentence is needed and it is not that clear how this follows the first part of the sentence in anycase
R3.10 Thanks for this comment We have removed the second part of the sentence.

Q3.11 line 57 effects of lineages on pneumococcal biology really needs reference(s) (could be a review given the breadth covered by this statement)
R3.11 Thank you for this comment We have now included citations to Lees et al., 2017 which demonstrates different carriage durations across lineages, Gladstone et al., 2019 which shows different invasiveness across lineages, and Lekhuleni et al., 2023 which shows variable distribution of lineages across invasive disease.

Q3.12 Fig 1C and D: mark when PCVs were introduced on the graphs
R3.12 We have now marked Figures 1C and D with vertical dashed black lines in 2009 and 2011.

Q3.13 Discussion lines 286-94 although i agree with the sentiment expressed here (about cuts to ART programmes), i also think it is tangential to the main data and messages from the paper and distracts from these
R3.13 Thank you for this comment, We agree and have now removed this paragraph. Especially alongside the new additions to the Discussion in response to the above comments it was extraneous.

Q3.14 lines 104 are these risks presented in a table somewhere for each geographical site and also given as rates are per 1000/100,000 of the population / year? if not please make one
R3.14 Yes, both the risks and rates per 100,000 are presented per province/district and month for each model structure in Table S3.

Q3.15 lines 110 to 113 i think these two sentences are connected ie the second sentence is used as evidence to suggest that travel to bigger hospitals increases the RR for large population centres? In which case the sentences best written the other way round and merged into a single sentence
R3.15 Thank you for this comment. We agree and have switched the order of these sentences.

Q3.16 lines 119-120 i can not see how ART would not reduce NVT IPD as well as VT; so was the lack of effect on NVT simply a numbers situation with too few NVT cases to show the effect of ART?
R3.16 We agree that this result is unexpected. We found that ART did not have a strong effect on NVTs in models from 2005-2020 however when we include data up to 2023 we do see an effect from ART (Figure S2). Post-COVID there was an increase in IPD cases among adults between 18-64 years old as compared to other age groups and this ART effect in this period might imply that the biggest impact of ART is on these adults. They were underrepresented among NVT invasive pneumococcal disease in the pre-COVID time period. The demographic shift in NVT may have provided the statistical power necessary to observe ART's protective benefit at a population level. The age group distributions are presented in Figure S2E. We have added a paragraph to highlight the possibility that the effect might be masked by lower numbers in the pre-COVID years (lines 152-163).

Lines 152-163 (tracked changes version):
"We found no effect from ART or PCV on NVT serotypes alone in the 2005-2019 period. A lack of PCV effect on NVTs is expected, as the vaccine does not protect against these serotypes. In contrast, ART is expected to confer protection against all pneumococcal serotypes by improving immune function in PLHIV, particularly among adults, who represent the group most likely to be receiving ART in this high HIV-burden setting[30]. In the 2005-2019 period, NVT IPD cases were skewed toward children and older adults but extending the analysis to 2020-2023 allowed an ART effect on NVTs to emerge. In this time period there were a higher number of IPD cases among adults aged 18-64 years who in this setting are the primary recipients of ART. This increase in adult cases may result from the resurgence of IPD after the COVID-19 pandemic and a gradual decline in indirect vaccine protection over time (Figure S2C-E)[29–33]."

26. Brueggemann, A. B. *et al.* Changes in the incidence of invasive disease due to Streptococcus pneumoniae, Haemophilus influenzae, and Neisseria meningitidis during the COVID-19 pandemic in 26 countries and territories in the Invasive Respiratory Infection Surveillance Initiative: a prospective analysis of surveillance data. *Lancet Digit. Health* **3**, e360–e370 (2021).
27. Heinsbroek, E. *et al.* Persisting high prevalence of pneumococcal carriage among HIV-infected adults receiving antiretroviral therapy in Malawi: a cohort study. *AIDS Lond. Engl.* **29**, 1837–1844 (2015).
28. Bertran, M. *et al.* Invasive pneumococcal disease 3 years after introduction of a reduced 1 + 1 infant 13-valent pneumococcal conjugate vaccine immunisation schedule in England: a prospective national observational surveillance study. *Lancet Infect. Dis.* **24**, 546–556 (2024).
29. Bennett, J. C. *et al.* Global impact of ten-valent and 13-valent pneumococcal conjugate vaccines on invasive pneumococcal disease in all ages (the PSERENADE project): a global surveillance analysis. *Lancet Infect. Dis.* **25**, 457–470 (2025).
30. Meiring, S. *et al.* HIV Infection and the Epidemiology of Invasive Pneumococcal Disease (IPD) in South African Adults and Older Children Prior to the Introduction of a Pneumococcal Conjugate Vaccine (PCV). *PLOS ONE* **11**, e0149104 (2016).

Q3.17 Fig 2D I am not at all sure what this is showing - it needs a better description presumably the insert is a blown up picture of the population density effect?
R3.17 We have edited the figure legend to make this more clear *"(e) the same population density fixed effect shown in d but with y-axis reduced (-8×10^{-4},0) to highlight the effect"*

Q3.18 Line 142 NAAQs first use here - the acronym needs defining
R3.18 We have now defined National Ambient Air Quality Standards (NAAQS)

Q3.19 Figure 3 and elsewhere with similar figures; the coloured shading represents the confidence intervals for these data??
R3.19 The following has been added to the figure legends "The shading and error bars represent 95% credible intervals."
